# Effects of Psychological Interventions to Enhance Athletic Performance: A Systematic Review and Meta-Analysis

**DOI:** 10.1007/s40279-023-01931-z

**Published:** 2023-10-09

**Authors:** Gustaf Reinebo, Sven Alfonsson, Markus Jansson-Fröjmark, Alexander Rozental, Tobias Lundgren

**Affiliations:** 1 Centre for Psychiatry Research, Department of Clinical Neuroscience, Karolinska Institutet, and Stockholm Health Care Services, Region Stockholm, Norra Stationsgatan 69, 113 64 Stockholm, Sweden; 2 Department of Psychology, Uppsala University, von Kramers allé 1A and 1C, Uppsala, Sweden

## Abstract

**Background:**

Psychological interventions are commonly applied in sports to help athletes enhance their performance, but the effect psychological interventions have on actual performance is unclear despite decades of research.

**Objective:**

We conducted a systematic review with meta-analyses to investigate the effects of a wide range of psychological interventions on performance in competitive athletes.

**Methods:**

A study protocol was preregistered in PROSPERO, and a literary search was performed in the MEDLINE, PsycINFO, Web of Science, and SPORTDiscus databases. Psychological intervention studies were eligible by using a group design and a quantitative performance outcome with athletes competing at a regional or university level or higher. Included studies were assessed regarding intervention characteristics, research methodology, and risk of bias. A multi-level meta-analysis framework with cluster robust variance estimation was used to quantitatively synthesize the results.

**Results:**

A total of 111 studies met the inclusion criteria, and 25 of these studies (37 effects) could be synthesized into five meta-analyses in which there were similarities in the type of psychological intervention, comparator, and experimental design. Meta-analyses I (multimodal psychological skills training vs control), II (mindfulness- and acceptance-based approaches vs control), and III (imagery vs control) consisted of parallel-group studies, and random-effects models were used to calculate the standardized mean difference. Meta-analyses IV (attentional focus strategies, external vs internal) and V (regulatory focus performance instructions, prevention vs promotion) consisted of counterbalanced crossover design studies, and random-effects models were used to calculate the standardized mean change using change score standardization. Significant results were found in three of the meta-analyses (I, II, and III). Psychological skills training (*g* = 0.83, 95% confidence interval 0.21–1.45), mindfulness- and acceptance-based approaches (*g* = 0.67, 95% confidence interval 0.01–1.32), and imagery (*g* = 0.75, 95% confidence interval 0.14–1.36) outperformed controls with moderate effects. However, when non-randomized trials and subjective performance outcomes were removed in sensitivity analyses, the overall estimates of the effect size were no longer significant in any of the syntheses.

**Conclusions:**

The significant moderate effects for psychological skills training, mindfulness- and acceptance-based approaches, and imagery are not stable, and further trials with robust research methodology, such as randomized controlled trials, are requested for all types of psychological interventions aiming to enhance performance in athletes. Moreover, improved reporting standards and the provision of datasets in open science repositories are important to consider in future trials in sport psychology.

**Clinical Trial Registration:**

PROSPERO CRD42017056677.

**Supplementary Information:**

The online version contains supplementary material available at 10.1007/s40279-023-01931-z.

## Key Points

**Table:** 

Psychological skills training, mindfulness- and acceptance-based approaches, and imagery interventions showed significant moderate effects on performance in athletes compared with controls.
Effects for psychological skills training, mindfulness- and acceptance-based approaches, and imagery were no longer significant when non-randomized studies and subjective performance outcomes were removed, which suggests that the effects are unstable and that further research with robust research methodology is needed.
Improved reporting standards and the provision of datasets in open science repositories should be considered by researchers in sport psychology in the future to increase transparency and aid interpretations of results.

## Introduction

As sport psychology moved into applied field research during the 1970s [1, 2], an interest in evaluating intervention effects naturally followed. Simultaneously, advancements of meta-analytic procedures in psychological sciences enabled ways to investigate aggregated effects [3]. Feltz and Landers' [4] meta-analysis of the mental practice of motor skills to enhance skill learning and performance was among the first systematic effect evaluations in applied sport psychology, and several have followed since. Two overviews of reviews were recently conducted. Lochbaum et al. [5] identified 30 meta-analyses of both correlational and intervention studies related to the sport psychology literature. Lange-Smith et al. [6] also identified 30 reviews but included various types of review methodologies in addition to meta-analyses (e.g., narrative), only including reviews of intervention studies. Thus, scrutiny of the evidence relating to psychological intervention techniques in sport psychology has proceeded for approximately 40 years. Although evaluation methods have varied over the years focusing on various sport-related outcomes, populations, and interventions, some vital research questions are still unanswered. Specifically, one such research question is whether psychological intervention techniques impact actual sport performance outcomes in clearly defined athlete samples. If this question is true, what are these interventions? To put this research question into context, we need to consider some of the characteristics of prior evaluations. Prior reviews have either focused on a single type of psychological intervention technique [4, 7–16] or several types of psychological interventions but contrasted between intervention types in the analysis [17–19], or mixed interventions without contrasting between different types of interventions in the synthesis [20]. Another core characteristic of prior reviews is to either investigate only athlete samples [18, 19] or include samples with a wide range of performance levels, including non-athletes [7–10, 12–16]. Another characteristic has been to either put the emphasis on actual sport performance outcomes [13, 14, 17–19] or instead include a wide range of outcome types that are not directly related to performance behavior, such as cognitive performance tasks [4, 7–9]. When reviews synthesize different types of psychological intervention techniques, samples, and outcome categories without dividing eligibility criteria (depending on the research question at hand) or by conducting proper subgroup analyses, only vague conclusions can be drawn. In general, the prior reviews mentioned above answer many important research questions for some of the intertwined fields of sport science, such as motor learning, performance enhancement, and sport and exercise psychology. However, the research question about whether and which psychological interventions have an effect on actual sport performance in samples verified as athletes still needs to be addressed with further clarity. This systematic review therefore investigates a wide range of psychological interventions with potential effects on performance in athlete samples with a verified competitive level at a regional or university level and higher. Furthermore, we examine the characteristics and quality of this research.

## Methods

### Data Availability and Transparency Statement

A study protocol [21] was developed using the guidelines of the Preferred Reporting Items for Systematic review and Meta-Analysis Protocols (PRISMA-P) [22, 23] and preregistered in the PROSPERO database. The search strategy (Online resource file 1) and a log of rejected studies (excluded full-text decisions according to PICO [Participants, Interventions, Comparator, and Outcome]; Online resource file 2) are available as supplementary information. Meta-analytic datasets and code are available online in the figshare repository [24]. We followed the PRISMA reporting guidelines for systematic reviews and meta-analyses for the final report [25, 26].

### Literature Search Strategy

A literature search was conducted in February 2017 and replicated twice, in February 2019 and April 2022. The searches were performed in the following databases: MEDLINE (OVID), SPORTDiscus, PsycInfo, and Web of Science. The MeSH terms identified for searching Medline (OVID) were adopted in accordance with corresponding vocabularies in PsycINFO and SPORTDiscus. As SPORTDiscus is a database specialized in sports, it includes controlled terms for several rare sports. Those sports were included in the controlled vocabulary searches because of their exploding functionality. They were not specified in the free-text searches. Each search concept was complemented with relevant free-text terms. The free-text terms were, if appropriate, truncated and/or combined with proximity operators. No language restriction was applied at the search stage. The searches were performed by librarians at the Karolinska Institutet University Library in collaboration with GR. The search strategies are available inOnline resource file 1. Additional hand-search procedures were applied. Reference lists of included studies and references citing included studies (in Web of Science) were screened for eligibility. Furthermore, other reviews and meta-analyses related to sport psychology were also screened for additional studies.

### Eligibility Assessment and Data Extraction Procedures

**Fig. 1:**
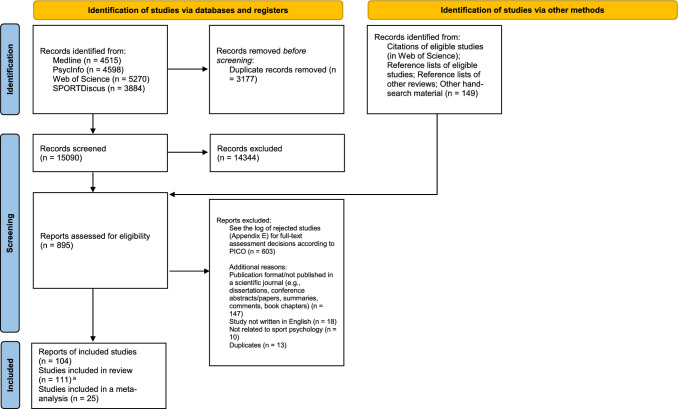
PRISMA (Preferred Reporting Items for Systematic reviews and Meta-Analyses) flow chart of the eligibility process [25]. ^a^Some reports (publications) included more than one study, therefore the number of studies exceeds the number of reports. *PICO* Participants, Interventions, Comparator, and Outcome

Through the screening process, two authors conducted all assessments independently. There were three pairs of assessors: GR-SA, GR-MJF, and GR-TL. Disagreements at the title/abstract level were decided by a third assessor in another pair of assessors. Eligibility coding, risk of bias ratings, and data extraction were conducted by two authors independently with two pairs of assessors: GR-SA and GR-MJF. Disagreements over the full-text eligibility coding, data extraction, or risk of bias ratings were solved through discussions with the third assessor in the other pair of assessors (SA or MJF). See Fig. 1 for the PRISMA flow diagram of the eligibility process. A log of rejected studies with decisions on excluded full-text assessments can be found in Online resource file 2.

### Criteria for Inclusion in the Systematic Review

Only studies written in English and published in scientific journals were considered for inclusion. Gray literature, conference papers, and dissertations were not considered. Eligibility criteria were defined using the PICO format [27] and described below.

#### Participants

Athletes eligible for inclusion were currently competing at a regional or university level or higher. Sufficient information regarding the performance context had to be stated in the report to verify the current level of performance. All sports were considered. Recreational, novice, junior/youth level athletes, and athletes competing on a local level were excluded. If samples were recruited from a competition (e.g., regional level) in which ineligible participants could take part (e.g., recreational participants in a marathon), additional information regarding the sample had to be described to be considered.

#### Designs

All group designs, both within-group and between-group (non-randomized and randomized) studies, were eligible for inclusion in the systematic review. For a study to be included in a meta-analysis, additional criteria were used (see Sect. 2.6). Single-case research designs were not included.

#### Psychological Interventions

Several psychological intervention techniques were included. However, for an intervention to be considered psychological, it had to be a method in which the independent variable primarily consisted of a psychological or psychotherapeutic focus. Methods in which the independent variable primarily consists of or directly focuses on technical, instructional, tactical, and biological target factors were not considered. The eligibility criterion was defined in this way to ensure that the investigated intervention effects were not due to, for example, the technical implication of a movement execution instruction. Therefore, interventions such as biofeedback/neurofeedback, attentional strategies and instructional self-talk with a sole technical focus on movement execution, quiet eye training, and the restricted environmental stimulation technique were not considered in this systematic review.

#### Outcomes

Only studies using a quantitative measure of sport performance were eligible. Both objective performance and subjective ratings of performance were considered. Objective outcomes had to be a result or a direct measure of performance of the studied sport. Indirect performance was not considered. Examples of indirect performance are performance-related measures (e.g., *V*O_2max_ for an endurance athlete) and other performance correlated measures (e.g., psychological constructs) that are not the performance itself (which for the endurance athlete could, e.g., be the completion time of an endurance activity). Additionally, for measures to be considered a direct measure of performance, they had to be a primary concern for the studied sport (e.g., endurance could be considered direct performance only in endurance sports). Subjective ratings of performance were either self-rated or by an external person (e.g., coach or expert rated). Subjective ratings had to originate from performances. Ratings of general athletic abilities (e.g., concentration skills, handling of emotions) were not considered. Other outcome measures in the included studies in addition to performance were not extracted to avoid a skewed representation of such measures as studies containing only non-performance or indirect performance outcomes were excluded.

### Risk of Bias Assessment

A risk of bias instrument adapted to psychological intervention research was developed. The primary aim for the item selection was the assessment of internal validity aspects rather than a full quality of trial assessment. Furthermore, the instrument was composed to enable the assessment of a range of group designs. Initially, other risk of bias or quality assessment tools, both general or traditionally used in medical research [28–31] and adapted versions to psychology/psychotherapy research [32–35], were screened and served as an item pool. GR compiled and selected items referring to internal validity issues. Additional items were considered and discussed among the study authors. A final selection of 12 items was chosen. The score range is zero to − 13, and a lower value indicates a higher risk of bias. All items of the instrument with the risk of bias assessments of the meta-analysed studies are presented in Table 1. The risk of bias assessments for the other studies in the systematic review can be found in Online resource file 3.

**Table 1:**
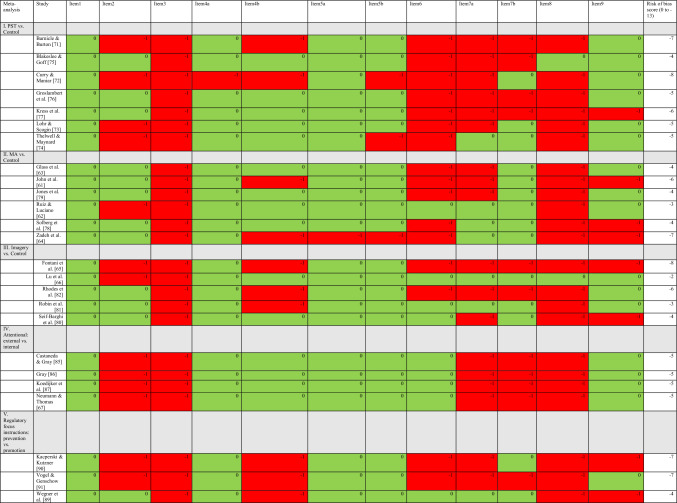
Risk of bias assessment of meta-analysed studies

Item 1: Is there a control group? (yes = 0, no =  − 2); Item 2: Is randomization conducted? (yes = 0, no =  − 1); Item 3: Study protocol registered a priori? (yes = 0, no =  − 1); Item 4a: Is the intervention protocol and procedure clearly described? (yes = 0, no =  − 1); Item 4b: Is intervention adherence investigated or a manipulation check conducted? (yes = 0, no =  − 1); Item 5a: An adequate primary outcome measure with acceptable psychometric qualities? (yes = 0, no =  − 1, if other measure than a psychometric instrument is adequately used as primary outcome = 0); Item 5b: Blinded outcome assessors? (yes = 0, no =  − 1, if not applicable to design = 0); Item 6: Clearly defined pre-specified primary outcome OR adjusted *p*-values for multiple testing? (yes = 0, no =  − 1); Item 7a: Intention-to-treat analysis? (yes = 0, no =  − 1); Item 7b: Reporting drop-outs? (yes = 0, no =  − 1); Item 8: Conducted interim analysis to detect deterioration OR investigated side effects? (yes = 0, no =  − 1); Item 9: Are effect sizes calculated OR a measure of clinical significance used for the primary outcome? (yes = 0, no =  − 1); *green* = yes/criterion fulfilled; *red* = no/criterion not fulfilled

*MA* mindfulness- and acceptance-based approaches, *PST* psychological skills training

### Inclusion Criteria in a Meta-analysis

All eligible studies in the systematic review were assessed for inclusion in a meta-analysis. For studies to be included in the same meta-analysis, the following characteristics of studies were considered:

- Similarity of psychological interventions and techniques.
- Similarity of comparator across studies.
- Comparable experimental design across studies (between-group comparisons vs counterbalanced within-group comparisons of two active experimental conditions).
- Sufficient study data were available to enable effect size calculation. Attempts were made to contact study authors for additional data if necessary to enable inclusion in a meta-analysis. Estimates of missing standard deviations were calculated when sufficient information was provided [36].

### Statistical Analysis

Analyses were conducted in the statistical software R (version 4.1.1) using the *metafor* (version 3.8–1) [37] and *clubSandwich* [38] packages. A multilevel meta-analysis (MLMA) framework [39] with cluster robust variance estimation (RVE) was used when studies included more than one performance outcome to handle dependency among effects, with an adjustment for small samples [40, 41]. Effect sizes were clustered within studies, and there was no information about sampling correlation; therefore, constant sampling correlation was assumed [40]. *r* = 0.5 was used for all MLMA, and sensitivity analyses were conducted with varying levels of *r*. A meta-analysis either included only parallel-group designs (inter-individual comparisons at post-experiment) or counterbalanced crossover designs (intra-individual comparisons between active experimental conditions that assume no carry-over effects between conditions). In meta-analyses I (psychological skills training [PST] vs control), II (mindfulness- and acceptance-based [MA] approaches vs control), and III (imagery vs control) consisting of parallel-group studies only, random-effects models were used to calculate the standardized mean difference (SMD) [42]. In meta-analyses IV (attentional strategies, external vs internal) and V (regulatory focus instructions, promotion vs prevention) consisting of counterbalanced crossover designs, random-effects models were used to calculate the standardized mean change (SMC) using change score standardization [43]. When the repeated-measures correlation was not known in the SMC analyses, *r* = 0.5 was used with accompanying sensitivity analyses. All primary studies in meta-analysis IV (attentional strategies, external vs internal) had one performance outcome measure; therefore, a univariate random-effects model was conducted. An MLMA framework was used for all other syntheses (I, II, III, and V). If primary studies included several related experimental conditions (e.g., two variants of external attention conditions) that were eligible for the same synthesis, then conditions were combined [36].

Statistical heterogeneity was examined with *I*^2^ statistics. In the MLMA models, the variance of both within-study and between-study heterogeneity was calculated. Publication bias was investigated through a test of excess statistical significance (TESS) and the proportion of statistical significance test (PSST). *Z* scores larger than 1.654 in either the TESS or PSST tests were interpreted as evidence for excess significance bias in the model [44]. An adapted version of Egger’s regression was also conducted to test for funnel plot asymmetry [45, 46]. A modified measure of precision was used as a predictor to remove artifactual correlations between effect sizes and their related standard errors [46, 47]. A visual inspection of funnel plots was also conducted.

Cohen’s [48] rule of thumb for effect-size magnitude categorization was used (small, 0.20–0.50; moderate, 0.50–0.80; large, > 0.80). Moderators were not investigated in any meta-analysis because of the risk of misleading results with fewer than ten studies [49]. Each sensitivity analysis commenced from the main results of the meta-analysis; studies were never subsequently removed. Datasets and code can be found online in the figshare repository [24].

## Results

First, the overall findings of the systematic review are described. Then, each meta-analysis is presented separately. Thirty-seven effects from 25 studies (*k*_studies_ = 25, *j*_effects_ = 37) distributed over five meta-analyses were synthesizable. For the meta-analysed studies, risk of bias ratings are found in Table 1, and study information is presented in Table 2.

**Table 2:** Study information for meta-analysed studies

Meta-analysis	Study	Sport and sample	Level of performance	Psychological intervention	Intervention duration	Design	Performance outcome	Other outcomes
I. PST vs control								
	Barnicle and Burton [71]	Soccer; female; *N* = 19; age nr	University	PST program with focus on sources of enjoyment (goal setting, relaxation, self-talk)	Twelve 45-min sessions (total time approximately 9 h)	Controlled trial	Soccer goals and assists (O)	Sources of Enjoyment in Youth Sport Questionnaire (SEYSQ); Competitive Motivational Style Questionnaire (CMSQ); Sport Anxiety Scale (SAS-2); Sport Confidence Inventory (SCI), Test of Performance Strategies-2 (TOPS-2P)
	Blakeslee and Goff [75]	Equestrian sport; female; *N* = 17; mean age 19.41 years	University	PST program (relaxation, imagery, goal setting, self-talk)	One initial 2-h session and then weekly 30-min sessions for three weeks (total time 3.5 h)	RCT	Flat performance scores by competition judges (O)^a^; fences performance scores by competition judges (O)^b^	Credibility questions (including asking for adverse effects)
	Curry and Maniar [72]	Various sports (type of sports not specified); sex nr; *N* = 168; age nr^c^	University	Cognitive and behavioral academic course in performance enhancement (applied strategies for: arousal/affect control, identifying purpose, goal setting, imagery, sport confidence, trust, flow, sport nutrition, on-/off-field problem solving, self-esteem, life skill education on eating disorders and drug/alcohol abuse)	A 15-week peak performance course with a homework workbook of eight assignments (total time 30 h)	Controlled trial	Coach-rated performance (S)	Other coach-rated parameters; homework data; hope scale; self-esteem scale; sport confidence inventory
	Groslambert et al. [76]	Biathlon; male and female; *N* = 16; mean age 21.5 years	International	Imagery + autogenic training	Two 30-min sessions/week during 6 weeks (total time 12 h)	RCT	Biathlon shooting performance (O)^a^; shooting tremor (O)^b^	Heart rate
	Kress et al. [77]	Cycling; male; *N* = 11; mean age 28.8 years	Regional	Stress inoculation training	1-h seminar of stress inoculation training followed by a week of participants practicing the skills on their own	RCT	Cycling performance times (ergometer cycle) (O)	Blood lactate level
	Lohr and Scogin [73]	Cross-country, basketball, track and field, golf, gymnastics, tennis, diving; male and female; *N* = 47; mean age 20.35 years	University	VMBR (self-administered)	An 18-day training program encouraged to be completed in 4 weeks: athletes received material in the form of a 42-page training manual, relaxation audiotape, and a visualization videotape	Controlled trial	Objective performance: times in competition (cross-country), shooting performance in practice (basketball), team statistics of “nine hole average” (golf), statistics from practice (track and field), serve performance (tennis), statistics from competition (gymnastics and diving). Subjective performance: coach-rated performance^a^, self-rated performance^b^	Sport Competition Anxiety Test (SCAT); visual imagery questionnaire (imagery ability)
	Thelwell and Maynard [74]	Cricket; male; *N* = 16; mean age 20.9 years	County/regional	PST program (goal setting, activation regulation strategies, self-talk, relaxation, imagery, concentration)	Twelve weekly sessions of 1 h (total time 12 h)	Controlled trial	For batters, runs scored, for bowlers, number of wickets taken in an innings (O)^a^; coach-rated performance (S)^b^	Credibility questions; qualitative data (interviews); modified version of the Mental Skills Questionnaire (mental skills use)
II. MA vs control								
	Glass et al. [63]	Various sports; male and female; *N* = 57; mean age 19.32 years	University	Mindfulness sport performance enhancement	Six weekly sessions of 75 min (total time 7.5 h)	RCT	Coach rating of overall performance (S)^a^; self-rating of overall performance (S)^b^	Depression, Anxiety, Stress Scale (DASS-21); Satisfaction with Life Scale (SWLS); Five Facet of Mindfulness Questionnaire (FFMQ); Acceptance and Action Questionnaire-II (AAQ-II); Dispositional Flow Scale-2 (DFS-2); Sport Anxiety Scale; Sport Rating Form (SRF); Coach’s Rating Form (CRF); mindfulness practice log and enjoyment rating
	John et al. [61]	Shooting; male; *N* = 110; mean age 29.5 years	National	Mindfulness meditation therapy	20-min sessions six times/week for 4 weeks (total of 24 sessions; total time 8 h)	RCT	Shooting performance (O)	Salivary cortisol as a measure of pre-competition anxiety
	Jones et al. [79]	Rowing; female; *N* = 27; age range 18–23 years	University	Mindfulness-based stress reduction	Six 75-min sessions over 6 weeks (intervention continued for 2 more sessions after post-assessment of ergometer performance)	RCT	Ergometer rowing time (O)	Athletic Coping Skills Inventory-28; Beck Anxiety Inventory; Beck Depression Inventory-II; Epworth Sleepiness Scale; Five Facet Mindfulness Questionnaire; Perceived Stress Scale; Pittsburg Sleep Quality Index; Ruminative Responses Scale; Scales of Psychological Well-Being; objective sleep measures via actigraph
	Ruiz and Luciano [62]	Chess; male; *N* = 10; age range 23–50 years	International	Acceptance and commitment therapy	2–3 sessions (4 h total for all)	Controlled trial	Elo rating of chess performance (O)	Acceptance and Action Questionnaire–II (AAQ–II); Chess Counterproductive reactions Questionnaire (CCRQ); rating of believability and interference of private events
	Solberg et al. [78]	Shooting; male and female; *N* = 25; age range 18–46 years	Regional	Meditation (ACEM meditation)	Seven weekly sessions, and also encouraged to home practice for 30 min daily during the intervention	RCT	Shooting performance scores, in competition (O)^a^ and in test shooting (O)^b^	Correlation between self-reported tension and test shooting performance
	Zadeh et al. [64]	Soccer; male; *N* = 44; mean age 24.15 years	Provincial	Mindfulness acceptance commitment approach	Seven weekly 45 min sessions (total time 5 h 15 min) + audio mindfulness exercises 3 days a week	RCT	Coach and expert ratings of individual performance (S)^a^ and team performance (S)^b^	Injury occurrence and severity; Mindful Sport Performance Questionnaire
III. Imagery vs control								
	Fontani et al. [65]	Karate; male; *N* = 30; mean age 35 years	National	Imagery (motor)	Daily imagery training for 16 min (120 trials) during 30 days	Controlled trial (imagery vs action trained control vs non-trained control)	Start reaction time (O)^a^; impact reaction time (O)^b^	Electromyography muscle activation; punch power and strength; movement-related brain macropotentials
	Lu et al. [66]	Basketball; male and female; *N* = 49; mean age 20.5 years	University	Imagery (PETTLEP, external^e^ vs internal^e^)	Prior to performance	Controlled trial (two experimental conditions and one control)	Basketball 3-point shooting (O)	The revised Chinese version of the Movement Imagery Questionnaire-Revised (MIQ-R); credibility questions
	Rhodes et al. [82]	Soccer; male; *N* = 30; mean age 24.3 years	National	Imagery (PETTLEP)^e^ vs functional imagery training^e^	3 sessions during 1 week (PETTLEP); 1 group session followed by daily implementation cues during 1 week (functional imagery training)	RCT (two experimental conditions and one control)	Soccer penalty kick (O)	Visual imagery vividness
	Robin et al. [81]	Basketball; sex nr; *N* = 36; mean age 19.53 years	Regional	Dynamic motor imagery^e^ vs dynamic motor imagery + video model observation^e^	30-min weekly session for five weeks (for both experimental conditions)	RCT (two experimental conditions and one control)	Basketball free throw shooting (O)^a^; free throw shooting including sprints [Evan Fournier Test] (O)^b^	Imagery vividness
	Seif-Barghi et al. [80]	Soccer; male; *N* = 17^c^; mean age 25.57 years	National	Imagery	Eight weekly sessions of 10–15 min (total time 80–120 min), participants were also encouraged to practice on a daily basis	RCT	Soccer passing performance (O)	None
IV. Attentional: external vs internal								
	Castaneda and Gray [85]	Baseball; male; *N* = 8^c^; mean age 19.5 years	University	Attentional focus intervention (skill/internal focus^e1^; skill/external focus^e1^; environmental/external focus^e2^; environmental/irrelevant focus^e2^)	Instructions prior to performance	Latin square counterbalanced crossover design with four experimental and one control condition	Mean temporal swing error in a baseball simulator (O)	Response accuracy of directing attention to the intended location
	Gray [86]	Baseball; sex nr; *N* = 10^c^; age nr	University	Attentional (internal/skill focus vs external/extraneous)	Instructions prior to performance	Crossover design with baseline followed by two counterbalanced experimental conditions	Mean temporal swing error in a baseball simulator (O)	Batting kinematics; secondary task (attentional) performance
	Koedijker et al. [87]	Table tennis, sex nr;* N* = 15^c^; mean age 19.1 years	Regional	Attentional (skill-focused/internal vs dual-task/extraneous-external)	Instructions prior to performance	Crossover design with three^a^ counterbalanced conditions (two experimental, one control)	Table tennis forehand performance (O)	Secondary task (attentional) performance; Slowed and speeded serve conditions (not related to attentional manipulations)
	Neumann and Thomas [67]	Golf; male and female; *N* = 16^c^; age nr^c^	State, national	Attentional (internal)^d^; attentional (external)^d^; goal setting (internal)^d^; goal setting (external)^d^	Instructions prior to performance	Crossover design with baseline followed by four counterbalanced experimental conditions	Golf putting performance (O)	Psychophysiological measures (heart rate; heart rate variability; respiratory frequency)
IV. Regulatory focus instructions: prevention vs promotion								
	Kacperski and Kutzner [90]	Table tennis; male and female; *N* = 39; mean age 30.07 years	Regional	Regulatory focus instructions (promotion vs prevention)	Instructions prior to performance	Crossover design with baseline followed by two counterbalanced experimental conditions	Winner of the match (O)^a^; winner of the first point after intervention instructions (O)^b^	Chronic regulatory focus orientation (adapted version of the Lockwood Scale); playing style (offensive/defensive)
	Vogel and Genschow [91]	Soccer; male; *N* = 20; mean age 22.7 years	Regional	Regulatory focus instructions (promotion vs prevention)	Instructions prior to performance	Counterbalanced crossover design with two experimental conditions	Scored soccer goals, penalties (11 m)^a^ and free-kicks (17 m)^b^ [O]	Chronic regulatory focus orientation (adapted version of the Lockwood Scale); coach-rated penalty and free-kick shooting ability (rated prior to performance)
	Wegner et al. [89]	Volleyball; male; *N* = 40; mean age 30 years	Regional	Regulatory focus instructions (promotion vs prevention)	Instructions prior to performance	Randomized crossover design with baseline followed by three counterbalanced conditions (two experimental, one control)	Volleyball service return (O)^a^; volleyball smash return (O)^b^	Chronic regulatory focus orientation (the Lockwood Scale)

*hr* hours, *MA* mindfulness- and acceptance-based approaches, *min* minutes, *O* objective performance measure, *nr* not reported, *PETTLEP* [imagery model] physical, environment, task, timing, learning, emotion, perspective, *PST* psychological skills training, *S* subjective performance measure, *RCT* randomized controlled trial, *VMBR* visuo-motor behavior rehearsal

^a^Outcome a in the meta-analysis

^b^Outcome b in the meta-analysis

^c^Additional study characteristics exist that did not meet eligibility criteria

^d^Conditions had other names in the article but are renamed to make the intervention content and comparison between studies clearer

^e^Groups were combined in the meta-analysis

### Systematic Review Results

A total of 111 studies from 104 reports met the eligibility criteria to be included in the systematic review (see Fig. 1). The performance levels of the samples were university athletes (54 of 111, 48.65%), regional athletes (15 of 111, 13.51%), county athletes (4 of 111, 3.6%), state athletes (2 of 91, 2.2%), provincial athletes (1 of 111, 0.9%), national athletes (17 of 111, 15.32%), international athletes (3 of 111, 2.7%), and samples with mixed performance levels (14 of 111, 12.61%). Research designs were uncontrolled trials (12 of 111, 10.81%), within-group time series designs (4 of 111, 3.6%), counterbalanced crossover designs (32 of 111, 28.83%), non-randomized controlled parallel-group designs (25 of 111, 22.52%), and randomized controlled parallel-group designs [RCTs] (37 of 111, 33.33%). Regarding performance outcomes, studies utilized only objective performance (88 of 111, 79.28%), only subjective performance (11 of 111, 9.91%), or a combination of subjective and objective performance outcomes (12 of 111, 10.81%). The mean risk of bias rating score was − 5.69 (range − 2 to − 11). For further details, study information of studies included in the systematic review is presented in Online resource file 4 (except for the meta-analysed studies that are presented in Table 2).

### Meta-Analysis I: PST (Multimodal) Versus Control

#### Included Primary Studies and Comparator

Studies that compared multimodal PST interventions with no-intervention controls were synthesized. Psychological skills training is an umbrella term for psychological techniques that aim to control or change mental states to enhance performance [50]. Psychological skills training is rooted in the 1950–1970s cognitive and behavioral methods, with interventions such as imagery/visualization, goal setting, attentional/concentration techniques, self-talk, and relaxation, to target mental preparation, arousal, or self-regulation to enhance performance [51]. In the current meta-analysis, multimodal PST packages consisted of two or more PST techniques, for example, imagery, relaxation, goal setting, self-talk, and attentional strategies. All included studies were parallel-group designs (*k* = 7, *j* = 11), and a random-effects MLMA with RVE was conducted.

#### Results

**Fig. 2:**
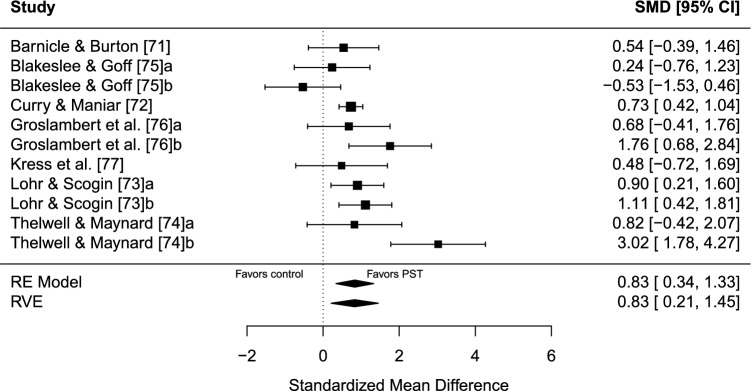
Forest plot for meta-analysis I illustrating the effect of multimodal psychological skills training on sport performance outcomes in athletes in comparison to controls. Effect sizes are the standardized mean difference (SMD [Hedges’ g]) with a 95% confidence interval (CI). Black squares are individual effect sizes and the square size represents the relative weight. Black rhombuses are the summary effect size estimate for the random-effect (RE) model with model-based variance estimates and with robust variance estimates (RVE). **a** and **b** are separate performance outcomes in the same study

**Fig. 3:**
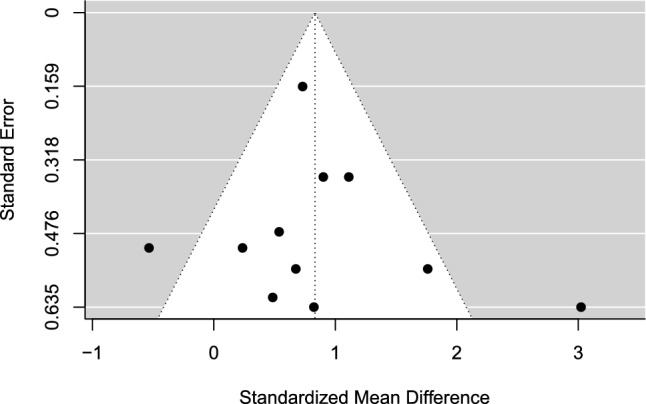
Funnel plot for meta-analysis I: multimodal psychological skills training versus control. *Black dots* are individual effect sizes with the standardized mean difference (Hedges’ *g*) on the *x-*axis and the standard error of the effect sizes on the *y-*axis. The dashed vertical line represents the summary effect size estimate in the meta-analysis model. Positive standardized mean difference values favor psychological skills training and negative standardized mean differences favor controls for the performance outcome. The plot does not account for the fact that some effect sizes are dependent because of being clustered within the same study

The estimated overall effect size for PST interventions compared with controls was significant, Hedges’ *g* = 0.83 (95% confidence interval [CI] 0.21–1.45, standard error [SE] = 0.25, *t* = 3.36, *p* = 0.017), in favor of PST. A forest plot is presented in Fig. 2. Overall heterogeneity was substantial (*I*^2^ = 70.39%), of which all 70.39% of the variance was estimated to be due to within-study heterogeneity and none due to between-study heterogeneity. No indication of publication bias was found. Both the TESS (− 0.37) and the PSST (0.17) were non-significant. Furthermore, the funnel plot was visually inspected for asymmetry (Fig. 3), and the relationship between the effect size estimate and precision was not significant (*t* =  − 0.19, *p* = 0.853).

#### Sensitivity Analyses

Removing non-randomized trials: when removing non-randomized trials, the remaining RCTs (*k* = 3, *j* = 5) demonstrated an aggregated non-significant effect of *g* = 0.51 (95% CI − 1.58 to 2.59, SE = 0.46, *t* = 1.09, *p* = 0.393), *I*^2^ = 60.64% (34.27% between-study heterogeneity; 26.36% within-study heterogeneity).

Only objective performance: subjective performance outcomes were removed. The remaining studies (*k* = 5, *j* = 7) demonstrated an aggregated non-significant effect of *g* = 0.56 (95% CI − 0.26 to 1.37, SE = 0.29, *t* = 1.95, *p* = 0.128), *I*^2^ = 39.99% (36.75% between-study heterogeneity; 3,24% within-study heterogeneity).

Sampling correlation: the average effect size and other model parameters were quite stable when sampling correlations were set to *r* = 0.2 and *r* = 0.8, respectively.

### Meta-analysis II: MA Approaches Versus Control

#### Included Primary Studies and Comparator

Mindfulness- and acceptance-based interventions that were compared to no-intervention controls were synthesized. Mindfulness- and acceptance-based interventions are inspired by developments that occurred in clinical psychology during the 1990s and early 2000s. These interventions (sometimes referred to as the “third wave” of cognitive and behavior therapy) target processes such as acceptance, mindfulness, values, metacognition, and attention [52]. A key theoretical implication of MA approaches was the accumulating evidence of the paradoxical effects in efforts to control, reduce, or suppress distressing thoughts and emotions [53–56], which was central to traditional PST to achieve optimal performance states through self-regulatory processes [50, 51]. Interventions represented in the current meta-analysis were acceptance and commitment therapy/training [57], mindfulness-based stress reduction [58], mindfulness-acceptance-commitment approach [59], mindfulness sport performance enhancement [60], meditation, and mindfulness meditation training. All included studies were parallel-group designs (*k* = 6, *j* = 9), and a random-effects MLMA with RVE was conducted.

#### Results

**Fig. 4:**
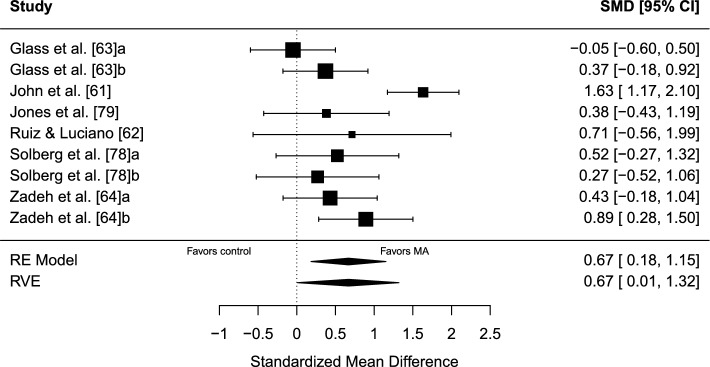
Forest plot for meta-analysis II illustrating the effect of mindfulness- and acceptance-based interventions on sport performance outcomes in athletes in comparison to controls. Effect sizes are the standardized mean difference (SMD [Hedges’ *g*]) with a 95% confidence interval (CI). Black squares are individual effect sizes and the square size represents the relative weight. Black rhombuses are the summary effect size estimate for the random-effect (RE) model with model-based variance estimates and with robust variance estimates (RVE). **a** and **b** are separate performance outcomes in the same study

**Fig. 5:**
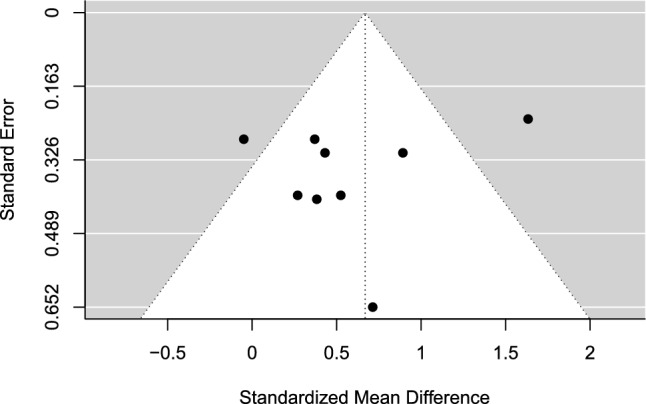
Funnel plot for meta-analysis II: mindfulness- and acceptance-based interventions versus control. *Black dots* are individual effect sizes with the standardized mean difference (Hedges’ *g*) on the *x-*axis and the standard error of the effect sizes on the *y*-axis. The dashed vertical line represents the summary effect size estimate in the meta-analysis model. Positive standardized mean difference values favor mindfulness- and acceptance-based interventions and negative standardized mean differences favor controls for the performance outcome. The plot does not account for the fact that some effects sizes are dependent because of being clustered within the same study

The estimated overall effect size was significant, *g* = 0.67 (95% CI 0.01–1.32, SE = 0.25, *t* = 2.67, *p* = 0.047), in favor of MA over control. A forest plot is presented in Fig. 4. Overall heterogeneity was substantial (*I*^2^ = 70.11%), of which 59.39% of the variance was attributed to between-study heterogeneity and 10.72% was attributed to within-study heterogeneity. No indication of publication bias was found. The TESS (− 4.24) and the PSST (− 1.55) were non-significant. The funnel plot was visually inspected for asymmetry (Fig. 5), and there was no significant relationship between the effect size estimate and precision (*t* =  − 0.84, *p* = 0.430).

#### Sensitivity Analyses

Removing potential outliers: John et al. [61] was removed as a potential statistical outlier. When removing John et al. [61], the remaining studies (*k* = 5, *j* = 8) demonstrated a non-significant aggregated effect, *g* = 0.41 (95% CI − 0.04 to 0.85, SE = 0.14, *t* = 2.94, *p* = 0.062), and heterogeneity was lower, *I*^2^ = 15.51% (0% between-study *I*^2^; 15.51% within-study *I*^2^). After removing John et al. [61], there was no indication of further outliers in the context of this model.

Removing non-randomized trials: when removing Ruiz and Luciano [62] as the only non-randomized trial, the remaining RCTs (*k* = 5, *j* = 8) demonstrated a non-significant aggregated effect, *g* = 0.66 (95% CI − 0.11 to 1.43, SE = 0.28, *t* = 2.4, *p* = 0.075), I^2^ = 74.92% (65.07% between-study *I*^2^; 9.85% within-study *I*^2^).

Removing subjective performance outcomes: Glass et al. [63] and Zadeh et al. [64] were removed as the only studies using subjective outcomes. The remaining studies (*k* = 4, *j* = 5) demonstrated a non-significant aggregated effect, *g* = 0.84 (95% CI − 0.36 to 2.03, SE = 0.36, *t* = 2.32, *p* = 0.109), *I*^2^ = 69.27% (69.27% between-study *I*^2^; 0% within-study *I*^2^).

Sampling correlation: the average effect size and other model parameters were quite stable when sampling correlations were set to *r* = 0.2 and *r* = 0.8, respectively.

### Meta-Analysis III: Imagery Versus Control

#### Included Studies and Comparator

Studies that compared unimodal imagery interventions with a no-intervention control condition were synthesized. Imagery comprises mental constructions of athletic scenarios for preparatory and practice purposes. Imagery can be used for motor skill acquisition, cultivation, or to mentally practice/prepare for competitive circumstances (e.g., high-pressure situations) [51]. If there was more than one imagery condition per study, then the conditions were combined. All included studies were parallel-group designs (*k* = 5, *j* = 7), and a random-effects MLMA with RVE was conducted.

#### Results

**Fig. 6:**
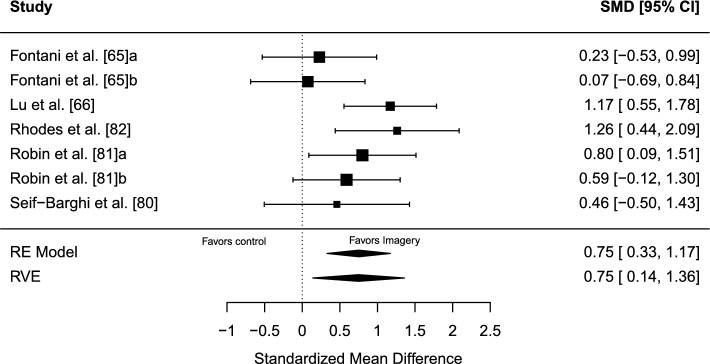
Forest plot for meta-analysis III illustrating the effect of imagery on sport performance outcomes in athletes in comparison to controls. Effect sizes are the standardized mean difference (SMD [Hedges’ *g*]) with a 95% confidence interval (CI). Black squares are individual effect sizes and the square size represent the relative weight. Black rhombuses are the summary effect size estimate for the random-effect (RE) model with model-based variance estimates and with robust variance estimates (RVE). **a** and **b** are separate performance outcomes in the same study

**Fig. 7:**
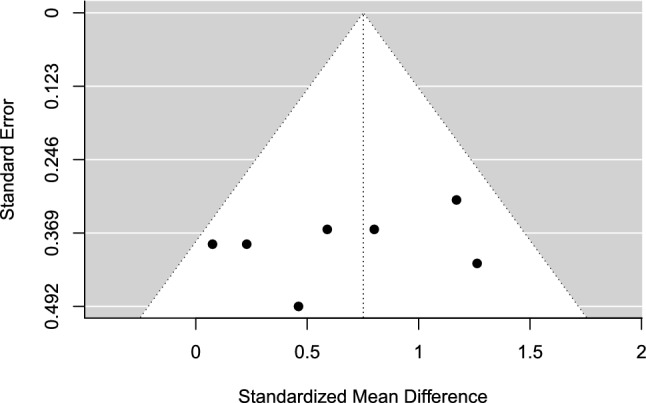
Funnel plot for meta-analysis III: imagery versus control. Black dots are individual effect sizes with the standardized mean difference (Hedges’ *g*) on the *x-*axis and the standard error of the effect sizes on the *y-*axis. The dashed vertical line represents the summary effect size estimate in the meta-analysis model. Positive standardized mean difference values favor imagery and negative standardized mean differences favor controls for the performance outcome. The plot does not account for the fact that some effects sizes are dependent because of being clustered within the same study

The estimated overall effect size was significant, *g* = 0.75 (95% CI 0.14–1.36, SE = 0.22, *t* = 3.49, *p* = 0.027), in favor of imagery over control. A forest plot is presented in Fig. 6. Overall heterogeneity was moderate (*I*^2^ = 39.77%), of which all 39.77% of the variance was attributed to between-study heterogeneity and none was due to within-study heterogeneity. No indication of publication bias was found. The TESS (− 0.86) and the PSST (PSST =  − 0.11) were non-significant. The funnel plot was visually inspected for asymmetry (Fig. 7), and there was no significant relationship between the effect size estimate and precision (*t* =  − 0.92, *p* = 0.398).

#### Sensitivity Analyses

Removing non-randomized trials: when removing non-randomized trials [65, 66], the remaining three RCTs demonstrated an aggregated non-significant effect of *g* = 0.81 (95% CI − 0.136 to 1.75, SE = 0.19, *t* = 4.23, *p* = 0.065), total *I*^2^ = 0%.

Sampling correlation: the average effect size and other model parameters were quite stable when sampling correlations were set to *r* = 0.2 and *r* = 0.8, respectively.

### Meta-Analysis IV: Attentional Focus, External Versus Internal

#### Included Primary Studies and Comparator

Attentional focus strategies are applied to enhance concentration and optimize performance by intentionally directing focus toward certain stimuli during performance. A common distinction is to either adopt an internal (e.g., focus on body or skill movements) or an external focus (e.g., skill-related external stimuli such as the ball leaving the bat or skill-unrelated stimuli such as sounds in the environment) of attention [11]. Counterbalanced crossover designs comparing internal and external attentional strategies’ effects on sport performance were synthesized (*k* = 4). If there was more than one internal (or external) condition in a study, conditions were combined [36]. As no study had more than one performance outcome, a univariate random-effects model was conducted to calculate the SMC. The repeated measure correlation was set to *r* = 0.5 except for one study [67], for which the correlation was known (*r* = 0.651).

#### Results

**Fig. 8:**
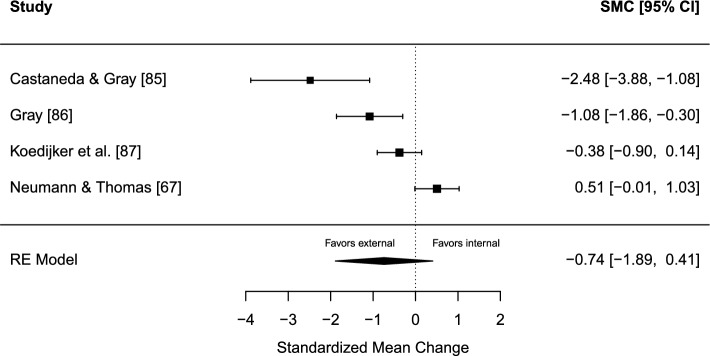
Forest plot for meta-analysis IV illustrating the effect of applying either an external or internal attentional focus during sport performance in athletes. Effect sizes are the standardized mean change (SMC) using change score standardization with a 95% confidence interval (CI). Positive SMC values favor an internal focus and negative SMCs favor an external focus on the performance outcome. Black squares are individual effect sizes and the square size represent the relative weight. The black rhombus is the summary effect size estimate for the random-effect (RE) model

**Fig. 9:**
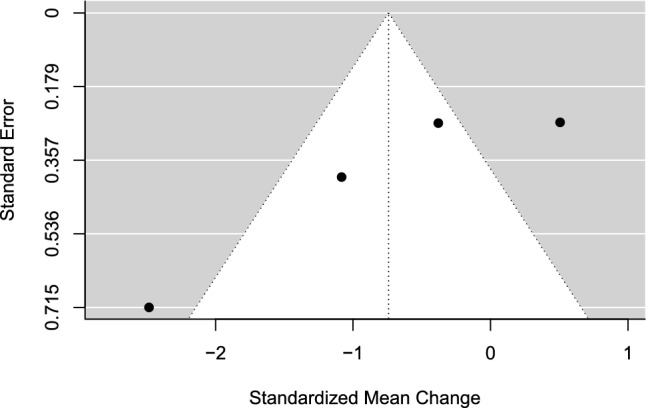
Funnel plot for meta-analysis IV: attentional focus, external versus internal. Black dots are individual effect sizes with the standardized mean change on the *x*-axis and the standard error of the effect sizes on the *y*-axis. The dashed vertical line represents the summary effect size estimate in the meta-analysis model. Positive standardized mean change values favor an internal focus and negative standardized mean changes favor an external focus on the performance outcome

The estimated overall effect size was not significant for any of the attentional focuses, SMC =  − 0.74 (95% CI − 1.89 to 0.41, SE = 0.59, *z* =  − 1.27, *p* = 0.205). A forest plot is presented in Fig. 8. Heterogeneity was considerable, *Q*(3) = 22.48, *p* < 0.001, *I*^2^ = 90.61% (95% CI 66.22 − 99.44). The TESS and the PSST were non-significant in both directions of the model, for external (TESS = 1.04; PSST = 0.69) and internal attentional strategies (TESS =  − 2.21; PSST =  − 0.97). The funnel plot was visually inspected for asymmetry (Fig. 9), and there was no significant relationship between the effect size estimate and precision (*t* =  − 3.36, *p* = 0.078).

#### Sensitivity Analyses

Repeated-measure correlation: correlation was set to *r* = 0.25 and *r* = 0.75, respectively, except for Neumann and Thomas [67], for which the correlation was known. Neither *r* = 0.25 nor *r* = 0.75 altered the results in a significant way.

### Meta-Analysis V: Regulatory Focus Instructions, Prevention Versus Promotion

#### Included Primary Studies and Comparator

Regulatory focus theory was developed by Higgins and proposes two different modes in self-regulation, a focus on accomplishment and aspirations (a *promotion* focus) or on safety and responsibility (a *prevention* focus) [68, 69]. Regulatory focus moves beyond classic motivational theories that suggest pleasure seeking and pain avoidance as the fundamental guide for behavior. Regulatory focus theory also emphasizes the fit between individual-level orientation (promotion or prevention chronic orientation) and performance instructions (promotion or prevention focus instructions), which may moderate the behavioral effect. Investigations on athletic samples and the regulatory focus instructions’ effect on performance began approximately a decade ago [70]. Counterbalanced crossover designs comparing the effects of prevention and promotion regulatory focus instructions on sport performance were synthesized (*k* = 3; *j* = 6). A random-effects MLMA with RVE was conducted to calculate the SMC. The repeated-measure correlation was not known and set to *r* = 0.5 across studies.

#### Results

**Fig. 10:**
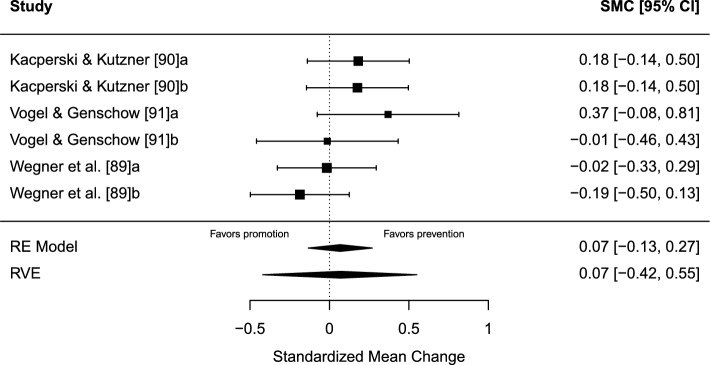
Forest plot for meta-analysis V illustrating the effect of applying either a prevention- or promotion-oriented regulatory focus instruction on sport performance in athletes. Effect sizes are the standardized mean change (SMC) using change score standardization with a 95% confidence interval (CI). Positive SMC values favor a prevention instruction and negative SMCs favor a promotion instruction on the performance outcome. Black squares are individual effect sizes and the square size represent the relative weight. Black rhombuses are the summary effect size estimate for the random-effect (RE) model with model-based variance estimates and with robust variance estimates (RVE).** a** and **b** are separate performance outcomes in the same study

**Fig. 11:**
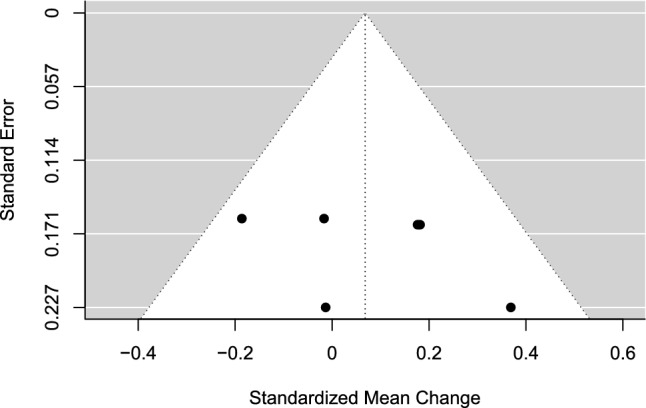
Funnel plot for meta-analysis V: regulatory focus instructions, prevention vs promotion. Black dots are individual effect sizes with the standardized mean change on the *x*-axis and the standard error of the effect sizes on the *y*-axis. The dashed vertical line represents the summary effect size estimate in the meta-analysis model. Positive standardized mean change values favor a prevention instruction and negative standardized mean changes favor a promotion instruction on the performance outcome. The plot does not account for the fact that some effect sizes are dependent because of being clustered within the same study

There was no significant difference in effect between prevention and promotion focus performance instructions, SMC = 0.07 (95% CI − 0.42 to 0.55, SE = 0.11, *t* = 0.65, *p* = 0.588). A forest plot is presented in Fig. 10. Heterogeneity was low, *I*^2^ = 20.64%, of which 18.6% was due to between-study heterogeneity and 2.04% was due to within-study heterogeneity. The test of excess statistical significance and the proportion of statistical significance test were non-significant in both directions of the model, for prevention (TESS =  − 1.42; PSST =  − 0.70) and promotion performance instructions (TESS =  − 0.79; PSST =  − 0.35). A visual inspection for funnel plot asymmetry was conducted (Fig. 11), and there was no significant relationship between the effect size estimate and precision (*t* = 0.58, *p* = 0.594).

#### Sensitivity Analysis

Repeated-measure correlation: varying repeated-measure correlations were tested (*r* = 0.75; *r* = 0.25) but did not alter the main results in a significant way.

Sampling correlation: the average effect size and other model parameters were quite stable when sampling correlations were set to *r* = 0.2 and *r* = 0.8, respectively.

## Discussion

### Meta-Analysis I: PST (Multimodal) Versus Control

Multimodal PST interventions significantly outperformed the no-intervention control on sport performance outcomes in athlete samples with moderate effects. However, sensitivity analyses showed a decline in effects and a non-significant result when non-randomized studies were removed from the synthesis [71–74]. Additionally, when removing subjective performance outcomes [72, 73], the effect estimate declined and was non-significant. Taken together, the sensitivity analyses cast doubt on the robustness of the effect estimate, emphasizing the importance of interpreting the results cautiously.

The mean risk of bias rating score was − 5.71 (range − 4 to − 8); see Table 1. The consistent weaknesses in research design across studies were that no single study had a preregistered study protocol, and did not prespecify a primary outcome or adjust for multiple testing. Only Thelwell and Maynard [74] conducted an intention-to-treat analysis, and only Blakeslee and Goff [75] investigated deterioration or side effects.

In future research, several concerns should be addressed to further strengthen the scientific robustness of PST research. Seven studies met the eligibility criteria to be included in the synthesis, and only three of these were RCTs. This suggests that additional trials are needed in general and randomized studies in particular. Furthermore, the sports included were soccer [71], equestrian sports [75], biathlon [76], cycling [77], cricket [74], and two studies with athletes from various sports [72, 73]. This implies a variation of sports represented in the synthesis, although the total number of sports is still limited. Future research would also benefit from comparing PST with other active psychological interventions. Direct comparisons would provide better insight into what type of intervention may be most beneficial in certain sports or performance contexts.

### Meta-Analysis II: MA Approaches Versus Control

Mindfulness- and acceptance-based approaches significantly outperformed no-intervention control on sport performance outcomes in athlete samples with moderate effects. However, the significant effect did not remain through the sensitivity analyses. The removal of a potential statistical outlier [61], the only non-randomized trial [62], and the use of subjective performance outcomes [63, 64] altered the average effect into a non-significant result in all sensitivity analyses. This calls for cautious interpretation of the robustness of the results. The limited number of studies (*k* = 6, *j* = 9) is another reason to make cautious interpretations.

The mean risk of bias rating was − 4.67 (range − 3 to − 7); see Table 1. Consistent weaknesses in research design were the lack of a prespecified study protocol and not investigating side effects. Except for Ruiz and Luciano [62], studies did not clearly predefine a primary outcome or adjust for multiple testing. Studies were randomized except for Ruiz and Luciano [62] and investigated intervention adherence except for John et al. [61] and Zadeh et al. [64].

Sports represented in the synthesis were shooting [61, 78], chess [62], rowing [79], soccer [64], and a sample with various sports (cross-country/track; swimming; tennis; lacrosse; field hockey; soccer; baseball; American Football; volleyball) in Glass et al. [63].

The limited number of studies (*k* = 6, *j* = 9) in this meta-analysis precluded extensive conclusions; however, MA interventions showed significant moderate effects to enhance performance in athlete samples. Additional trials investigating the effect of MA interventions on athlete performance are needed, preferably with robust research methodology designs such as RCTs.

### Meta-Analysis III: Imagery Versus Control

Imagery significantly outperformed control with an overall moderate effect. However, the effect was no longer significant when the non-randomized trials were removed in a sensitivity analysis, which also improved heterogeneity. This suggests that the results should be interpreted cautiously and that additional trials with robust methodologies, such as RCTs, are needed to further establish imagery intervention effects on the performance of athletes. Additional trials would also enable investigations of moderating effects.

The mean risk of bias rating was − 4.6 (range − 2 to − 8); see Table 1. A consistent weakness in research design was the lack of a preregistered study protocol. Only Lu et al. [66] investigated side effects. Another common weakness was the lack of investigating intervention adherence/manipulation checks, except for Lu et al. [66] and Seif-Barghi et al. [80]. Additionally, only Lu et al. [66] and Robin et al. [81] conducted intention-to-treat analyses. Of the total five studies, three were randomized [80–82], and the other two were non-randomized [65, 66].

Sports represented in the analysis were karate [65], soccer [80, 82], and basketball [66, 81], making team ball sports the main context of study. Additionally, only one study included female athletes [66]. This is a limitation for the generalizability of the results.

### Meta-Analysis IV: Attentional Focus, External Versus Internal

There was no significant difference associated with applying an external or internal attentional focus on sport performance in athlete samples. The number of studies in the analysis was limited (*k* = 4), which resulted in a poor basis for funnel plot inspection. Additionally, the statistical heterogeneity was considerable. It cannot be ruled out that the obtained non-significant results were due to low power, which is affected by the number of studies, sample size, and heterogeneity in the analysis [83, 84].

The risk of bias score was − 5 across all studies with the exact same item ratings (Table 1). Consistent design weaknesses were the lack of a preregistered study protocol, no randomization of order of experimental conditions, dropouts were not reported, and no side effects were investigated.

Sports and outcomes included were baseball batting performance [85, 86], table tennis forehand performance [87], and golf putting performance [67]. Although there are many differences between these sports, there are some similarities regarding the motor skill of hitting a ball in all performance outcomes. The internal focus conditions were skill-focused across studies. However, the external focus conditions varied across studies with both skill-irrelevant/extraneous external focus [85–87] and skill-relevant external focus [67, 85]. Hence, stimuli foci can differ between two external focus conditions, although both are still considered external focus strategies. This can also be the case for different types of internal focus strategies. However, this meta-analysis can only draw conclusions based on the broad distinction between internal and external attentional focus strategies, which is another limitation of the current synthesis.

Prior reviews have concluded that an external focus is preferred over an internal focus for sport and motor performance [11, 88]. Such conclusions could not be drawn based on the current synthesis. However, the results of the current synthesis should be interpreted with caution. In future research, further studies with robust research designs comparing internal and external focus strategies in athlete performance are warranted for further evaluation of potential effects. If sufficient experiments are conducted across different sports and motor skills in athletes, more nuanced conclusions can be made regarding the most effective strategy for a given skill in a specific sport. Counterbalanced crossover designs such as the studies in the current meta-analysis are a feasible research design alternative, especially if carryover effects are assessed and controlled for with manipulation checks. This also has the potential to increase sample sizes. However, randomized parallel-group studies are another useful option if the prerequisites are sufficient.

### Meta-Analysis V: Regulatory Focus Instructions, Prevention Versus Promotion

There was no significant difference in the effect of applying prevention- or promotion-oriented regulatory focus instructions in athletes on sport performance outcomes. The limited number of studies in the synthesis (*k* = 3; *j* = 6) suggests that the results should be interpreted cautiously.

The mean risk of bias rating score was − 6 (range − 4 to − 7); see Table 1. Consistent weaknesses in research design were the lack of a prespecified study protocol and not investigating intervention adherence with, for example, manipulation checks. No forms of side effects were investigated. Randomization of order for the experimental manipulations was only utilized in Wegner et al. [89]. Sports represented in the synthesis were table tennis [90], soccer [91], and volleyball [89].

The non-significant results were expected and in line with the core hypothesis of the regulatory focus literature, namely, that the regulatory focus instruction effect is moderated by the fit of the chronic regulatory focus orientation to the individual [89–91]. The current meta-analysis showed no significant differences in effect and was limited to regulatory focus instructions only and does not contain data on chronic regulatory focus orientation. In future research, an individual participant data meta-analysis that considers chronic regulatory focus orientation may further elucidate the effect of regulatory focus instructions on athlete performance.

### General Discussion

Scrutiny of the evidence relating to psychological interventions’ effect in sport has proceeded over four decades investigating different types of interventions, sport populations, and performance outcomes [6]. This systematic review investigated a wide range of intervention types in studies focusing on only athlete samples and direct performance outcomes to bring further clarity to the understanding of psychological intervention effects in competitive athletes. Furthermore, the characteristics of such research were examined. In total, 111 studies met eligibility for the systematic review, and 25 (*j* = 37) of these were quantitatively synthesized in five separate meta-analyses (I, PST multimodal vs control; II, MA vs control; III, imagery vs control; IV, attentional, external vs internal; V, regulatory focus instructions, prevention vs promotion). Moderate significant effects were found in meta-analyses I, II, and III, in which multimodal PST, MA interventions, and imagery outperformed no-intervention controls for sport performance outcomes in athletes. However, when non-randomized trials were removed in sensitivity analyses (in meta-analyses I, II, and III), the effects were non-significant. Additionally, when subjective performance outcomes were removed in sensitivity analyses (in meta-analyses I and II), the effects were no longer significant. Therefore, the moderate effects in meta-analyses I, II, and III cannot be considered stable and should be interpreted with caution. Meta-analyses IV and V differ from I, II, and III as active experimental conditions (psychological techniques) were compared in counterbalanced crossover design studies. Such experimental designs (a.k.a. Latin-square designs) are based on paired data and assume no carry-over effects between conditions. Furthermore, the purpose of the counterbalancing procedure is to remove bias due to order and periodic effects [92]. In meta-analysis IV, no significant differences were found when comparing an external or internal focus of attention on athlete performance, although the results should be interpreted with caution owing to the limited number of studies in the synthesis. Finally, in meta-analysis V, there were no significant differences in applying prevention- or promotion-oriented regulatory focus performance instructions. Taken together, additional trials with robust research methodology, such as RCTs, are required for all types of psychological interventions to further investigate their effect on athlete performance.

The considered outcomes in this systematic review had to be a direct measure of sport performance, and similar criteria have been used in prior meta-analyses in sport psychology [93, 94]. For a meaningful understanding of whether the effect is relevant or not, its real-world meaning should be contextualized when possible [95], and effect size interpretation in sport psychology is more straightforward when studies use outcomes that incorporate performance-relevant behavior [96]. As the meta-analyses in this systematic review included a variety of sports and performance outcomes, a nuanced interpretation of the real-world meaning of the performance change is difficult to make and not suggestible based on the overall effect estimates. Furthermore, the starting point of an effect size interpretation is preferably its CI [97]. This should be considered for the significant results in meta-analyses I (Fig. 2), II (Fig. 4), and III (Fig. 6). All CIs are comparatively wide, which is another reason for interpreting the results carefully.

An advantage of including a wide range of psychological interventions is to enable comparisons across interventions. Prior reviews have commonly focused on one type of psychological intervention [4, 7–15], with some exceptions [17, 19], including the current review. Unfortunately, only two studies in the systematic review directly compared PST and an MA intervention [98, 99], which was not enough to conduct a meta-analysis. However, further direct comparisons of PST and MA may provide better insight into what type of psychological intervention may be beneficial in various sport contexts.

Many risk of bias assessment tools assess only one type of study design, for example, RCTs, such as the Cochrane Collaboration’s tool for assessing risk of bias [30]. As this systematic review contains a wide range of group designs, an instrument was developed to enable assessments across varying designs (uncontrolled, non-randomized, randomized, and with either parallel-group or counterbalanced crossover designs) but with criteria relevant to the risk of bias in experimental trials of psychological interventions in general. This was the reason for the deviation from the study protocol for which the initial plan was to use CONSORT items in full [21]. Because group designs varied, an item could formally assess different design features, although it pertained to an adequate rating of bias for that type of study design. For instance, Item 2 assesses whether randomization is conducted. For parallel-group designs, this refers to traditional randomization procedures. However, for counterbalanced crossover designs, the item assesses whether the order of experimental conditions was randomized to account for bias due to periodic effects [92]. Therefore, comparisons of risk of bias ratings should only be performed between studies with comparable experimental designs, as in meta-analyses I (PST, mean risk of bias score =  − 5.71, range − 4 to − 8), II (MA, mean =  − 4.67, range − 3 to − 7), and III (imagery, mean =  − 4.6, range − 2 to − 8) which contained only parallel-group studies (see Table 1).

### Limitations

The results of a systematic review and meta-analysis may be influenced by study reporting (reporting bias), as some excluded studies may in fact be eligible, although underreporting makes this difficult to verify [100]. The use of performance levels as an eligibility criterion was to enable answers to the research question regarding the performance context of competitive athletes and not recreational, novice, or junior participants. Nor were local level athletes considered in this systematic review. Similar approaches for defining and assessing sample eligibility criteria have been used in prior reviews, for example, [93]. This can distinguish between competitive athletes and samples that, for example, may still learn/acquire basic skills in sports, which was not the focus of this review. It may also account for other differences in the performance contexts for competitive and non-competitive athletes that the psychological intervention targets to a larger extent as a function of competitive level (e.g., handling high-pressure situations). The log of rejected studies (Online resource file 2) shows that among the 603 excluded reports in the full-text assessments, 417 experiments/studies were excluded on the participant criterion. We naturally do not know how many were excluded because of underreporting. However, there may be a problematic low consensus of how/which sample characteristics should be reported in sport psychology [101]. Therefore, we will extend our reasoning and declare our decision process regarding this criterion and the use of regional- and university-level athletes as the minimum of performance expertise. University athletes had to be on a university team and/or compete in intercollegiate competitions. University “sports clubs” and intramural participation were thus excluded. High school athletes were considered equal to junior level participants and were therefore excluded. When sample ages were reasonably young but not explicitly reported as competing on a junior or senior level, age standards of senior level participation of international sport federations were used when such guidelines existed (e.g., gymnasts under 15 years of age do not compete in senior level competitions according to the International Gymnastics Federation). The comparison of performance level between sports and countries is not clear-cut. This is aggravated by the available levels of expertise in different sports within a country, further complicated by international differences owing to the type of sport. However, performance level, referring to the geographical level of competition standards (e.g., local, regional, county, state, provincial, national, and international), has been used as a central criterion in taxonomy classification systems of athletic expertise [101, 102] and therefore was used as a key criterion in this study. Other reviews in sport psychology have used similar criteria [20]. As geographical definitions vary between countries, terms such as county, provincial, sectional, and state were considered comparable. Eligibility assessments were based on available sample information to verify the current performance level. However, it did not solely rely on terms such as “elite” because such terms have been shown to represent immense variation and inconsistency of actual performance level [101] and therefore are not possible to verify without additional information. For the same reason, the sole use of related terms, such as “semiprofessional” or “professional” was not considered without additional information (e.g., type of league or competitions that the athletes participated in) that could verify the competitive level. This also implies that “amateur” athletes could be included when competitive level was reported and eligible. The verification of performance level is necessary for feasible comparisons of study samples with athletes from different sports and countries and crucial in making valid conclusions about existing evidence. Hence, this is important for future research to consider as part of the methodological reporting of trials. Underreporting of study sample characteristics cannot be ruled out as a potential source of lost data for the current review.

This review emphasizes that eligible psychological interventions primarily consist of a psychological or psychotherapeutic focus. Psychologically related methods in which the independent variable primarily consisted of or directly focused on technical, instructional, tactical, and biological target factors were not considered. Naturally, psychological interventions and techniques commonly target sport behaviors as the very purpose of a sport psychology intervention may be to aid such behaviors. However, some techniques are so technically oriented around how to execute a certain motor behavior (e.g., “lift your knees high”) that it becomes unclear whether the technique is still to be defined as a psychological or technical/instructional technique per se. There are, for instance, both self-talk techniques and attentional focus techniques that primarily focus on the technical execution of behavior. However, as the aim of the current article was to review the effect of psychological interventions and techniques, clarity of independent variables becomes a crucial issue to ensure that investigated effects are not due to, for example, the technical implication of a movement execution instruction. Although self-talk and attentional techniques that focus on technical execution may have a logical place in aiding certain sport behaviors, they did not meet the criteria for psychological intervention techniques in this review. As such, the current review does not contain full account of attentional and self-talk techniques. There are other reviews that use a wider framework for the inclusion of samples and interventions with both psychological and technically oriented independent variables for attentional focus interventions [11, 88] and self-talk interventions [103].

A power analysis is an important part of planning a meta-analysis [84] but was not conducted prior to this systematic review. Power refers to the ability of an analysis to correctly reject the null hypothesis of no effect when a true effect exists. For random-effects meta-analyses, power can be affected by factors such as the number of included studies, sample size and the statistical heterogeneity of included effects [83, 84]. Power is a likely issue for the interpretation of the results found in this study for a few reasons. The significant effects found in meta-analyses I–III were accompanied by sensitivity analyses to investigate whether the removal of studies with certain characteristics, such as using an inferior study design (non-randomized trials) or subjective performance measures, affected the results. As studies were removed in the sensitivity analysis, power was probably reduced compared with the full meta-analyses that could be considered small to begin with. This weakens the interpretations of whether the non-significant results in the sensitivity analyses suggest a non-significant overall effect when considering only superior research designs (RCTs) or if it reflects a loss of power that fails to detect a true intervention effect that exists. Additionally, meta-analysis IV that compared attentional strategies (external vs internal focus) only had four effect sizes. Thus, the non-significant trend of favoring an external focus could be a result of low power. The number of studies in the meta-analyses is a limitation to the interpretation of the results for other reasons in addition to the issue of power. All five meta-analyses had too few studies (PST, *k* = 7, *j* = 11; MA, *k* = 6, *j* = 9; imagery, *k* = 5, *j* = 7; attentional, *k* = 4; regulatory focus instructions, *k* = 3, *j* = 6) to conduct meta-regression analyses to investigate moderating effects because of the risk of misleading results with fewer than ten studies [49]. This makes it difficult to conclude to what extent certain study design features, risk of bias implications, or other experimental differences influence intervention effects. Furthermore, meta-analyses I (PST) and II (MA) consist of psychological intervention packages considered similar enough to synthesize as they share many common intervention procedures; however, there are still differences in the interventions between studies. Both PST and MA meta-analyses have different combinations of intervention procedures and protocols (see Table 2). Ideally, with additional trials, subgroups of intervention protocols could have been investigated or to what extent different intervention protocols moderated the average effect. Another limitation associated with small-numbered meta-analyses is that the basis for inspection of funnel plot asymmetry is rather poor, which weakens the interpretation of potential publication bias [104].

Another related limitation is that only 25 studies (*j* = 37) from the systematic review were quantitatively synthesized, which means that 86 studies (77.48%) were not. To be included in a meta-analysis, primary studies had to be similar across psychological intervention techniques, comparators, and experimental designs and present sufficient data to enable effect size calculations (and attempts to contact study authors were made when needed). These factors are the reasons why no further studies in the systematic review were included in a meta-analysis. However, some potentially useful data remain in the systematic review that together with a few additional studies could be included in further separate meta-analyses in the future for interventions such as hypnosis or some of the remaining PST single modal interventions (e.g., relaxation, self-talk, or goal setting), or for active comparisons between interventions (e.g., PST vs MA). The full list of studies in the systematic review not included in any of the meta-analyses can be found in Online resource file 4.

## Conclusions

Significant moderate effects on athlete performance were found for PST, MA, and imagery interventions compared with controls. However, the results were no longer significant when non-randomized studies and subjective performance outcomes were removed from the syntheses. This suggests that the effects were not stable and that conclusions should be drawn with caution. Another two meta-analyses were also conducted. Attentional interventions comparing external and internal focus strategies showed no significant differences in athlete performance. Finally, prevention- and promotion-oriented regulatory focus performance instructions were compared and showed no significant differences. In general, improved research methodology, reporting standards, and the provision of datasets in open science repositories are important to consider in future trials investigating the effect of psychological interventions on performance in athletes. Because of the limited number of studies in all five current meta-analyses, additional trials with robust methodology are needed for all types of psychological interventions with the aim of enhancing performance in athletes to further establish the effects found in this review and to enable additional investigations of moderating effects.

### Supplementary Information

Below is the link to the electronic supplementary material.

Supplementary file1 (DOCX 81 KB)

Supplementary file2 (DOCX 230 KB)

Supplementary file3 (DOCX 41 KB)

Supplementary file4 (DOCX 50 KB)

## Declarations

### Availability of data and material

The datasets are available online in the figshare repository [24].

### Code availability

The code is available online in the figshare repository [24].
