# Supplementary material for: Effects of Psychological Interventions to Enhance Athletic Performance: A Systematic Review and Meta-Analysis
Source: Sports Med. 2023 Oct 9;54(2):347–73. doi: 10.1007/s40279-023-01931-z (PMC10933186; doi:10.1007/s40279-023-01931-z)
Supplement: Supplementary file 1 — Supplementary file1 (DOCX 81 KB) [file 40279_2023_1931_MOESM1_ESM.docx]

**Supplementary information. Online Resource 1.**

*Article:* Effects of psychological interventions to enhance athletic performance: A systematic review and meta-analysis

*Journal:* Sports Medicine

*Authors:* Gustaf Reinebo, Sven Alfonsson, Markus Jansson-Fröjmark, Alexander Rozental, Tobias Lundgren

*Corresponding author:* Gustaf Reinebo, email [gustaf.reinebo@ki.se](mailto:gustaf.reinebo@ki.se) , Centre for Psychiatry Research, Department of Clinical Neuroscience, Karolinska Institutet, & Stockholm Health Care Services, Region Stockholm, Norra stationsgatan 69, SE-113 64, Stockholm, Sweden

Documentation of search strategies

University Library search consultation group

Date: January-February 2017, last update April 2022

Topic/research question: What is the evidence for psychological methods (I, C) used to enhance the performance (O) of athletes (P)?

Name of researcher(s): Gustaf Reinebo

Librarian(s): Susanne Gustafsson, Magdalena Svanberg, & Narcisa Hannerz

Databases:

1. Medline (Ovid)
2. PsycInfo (Ebsco)
3. Web of Science
4. SPORTDiscus (Ebsco)

Total number of hits:

- Before deduplication: 18,267^a^
- After deduplication: 15,090^b^ (9,365^c^)

Comments:

a = total from last search in April 2022; b = after deduplication from searches in February 2017, February 2019 and April 2022 combined; c = after deduplication in original search in February 2017.

A replicated update of the search was conducted in February 2019, with the following result: Medline (n = 440), PsycInfo (n = 312), Web of Science (n = 1,081), SPORTDiscus (n = 371). After duplicate records were removed (n = 540), the total number of records from search two was n = 1,664.

A second replicated update of the search was conducted in April 2022, using a variant of the method describe in Bramer W, Bain P. Updating search strategies for systematic reviews using EndNote. J Med Libr Assoc. 2017 Jul;105(3):285-289. doi: 10.5195/jmla.2017.183. Epub 2017 Jul 1. PMID: 28670219; PMCID: PMC5490709. After duplicate records were removed, the total number of records from search 3 was n = 4,061 (Medline = 1,195; Psycinfo = 762; Web of Science = 1,364; SportDiscus = 740).

1. Medline (Ovid)

| Date of Search: 202-04-13  Number of hits: 4,515  Comments: | Field labels: .ti,ab,kf. = title, abstract, keyword exp/ = MeSH, exploded / = MeSH, not exploded adjx = within x number of words |
| --- | --- |
| 1. Athletes/  2. Sports/  3. Baseball/  4. Basketball/  5. Bicycling/  6. Boxing/  7. Dancing/  8. Football/  9. Golf/  10. Gymnastics/  11. Hockey/  12. exp Martial Arts/  13. Mountaineering/  14. exp Racquet Sports/  15. Running/  16. Skating/  17. exp Snow Sports/  18. Soccer/  19. Sports for Persons with Disabilities/  20. exp Swimming/  21. exp "Track and Field"/  22. Volleyball/  23. Walking/  24. Weight Lifting/  25. Wrestling/  26. Youth Sports/  27. (aikido or alpinis* or archer* or athlete* or athletics or badminton or ballgame* or ball game* or bandy or baseball or basketball or biathl* or bicycle or bicycling or billiard or bobsleigh* or bowling or bowler* or boxing or boxer* or canoe* or cheerlead* or cheer lead* or cricket or curling or cycling or cyclist* or danc* or dart or darts or decathl* or discus or diver or divers or diving or dressage* or duathl* or eventing or equestrian polo or fencer* or fencing or floorball or floor ball or football or golf* or gymnast* or hammer throw* or handball* or hepathl* or hockey or hurdler* or javelin throw* or judo or jujitsu or karate or kayak* or kung fu or lacrosse or luge or olympic* or orienteer* or marathon* or martial art* or motorbike* or motorsport* or mountain climb* or mountaineer* or parathlet* or para-athlet* or para athlet* or parasport* or pentathl* or pole vault* or racewalk* or race walk* or racquetball or racketball or racket ball or rally or rider* or rodel or rower* or rowing or rugby or running or runner* or sail* or shot put* or shooter* or shooting* or skateboard* or skater* or skating or skeleton or skier* or ski or skiing or snooker* or snowboard* or soccer or softball or squash or speedskat* or sport* or steeplechase* or surfer* or surfing or swim* or tae kwon do or taekwondo or tai or thai or tai ji or taiji or taijiquan or taichi or team game* or tennis or "track and field*" or triathl* or trampoline or volleyball or water polo or weight* lifting or lifting weight* or weightlifting or windsurf* or wrestl* or wushu).ti,ab,kf.  28. ((high or long or triple or equestrian or ski) adj1 jump*).ti,ab,kf.  29. or/1-27  30. Goals/  31. Relaxation/  32. Relaxation Therapy/  33. Self Concept/  34. Self Efficacy/  35. Motivation/  36. exp Psychotherapy/  37. exp Cognitive Therapy/  38. (acceptance-commitment or "acceptance and commitment therap*" or achievement* or active listening or activation regulation or applied sport psycholog* or applied behavior analys* or biofeedback or bibliotherap* or breathing exercise* or cognitive remediation or counsel?ing or cotherap* or desensitization or directed reverie therap* or dream analy* or flow or goal motivation* or goal set* or goal striv* or guidance or hypnotherap* or hypnosis or imagery or logotherap* or mac or meditation or meditating or mindful* or mirroring or motivational interview* or mutual storytelling or paradoxical technique* or pettlep or psychoanaly* or psychodrama* or psychodynam* or psychological performance enhancement techniq* or pet or preperformance routine* or pre performance routine* or psychotherap* or qigong or qi gong or relaxation or therapeutic alliance* or therapeutic communit* or thought stopping or transactional analy* or visualisation or visualization or yoga).ti,ab,kf.  39. (self belief* or self concept or self confidence* or self determination or self doubt* or self efficac* or self esteem or self instructional or self monitoring or self perception* or self talk*).ti,ab,kf.  40. (cognitive program* or cognitive psychotherap* or cognitive restructuring* or cognitive strateg* or cognitive therap* or cognitive train*or cognitive behavio* program* or cognitive behavio* psychotherap* or cognitive behavio* restructuring* or cognitive behavio* strateg* or cognitive behavio* therap* or cognitive behavio* train* or cognitive skill* training or cognitive skill* therap*).ti,ab,kf.  41. ((acceptance or adlerian or anger management or aversive or aversion or autogenic or behavio* or brief or client centered or commitment or conversion or emotion* or emotion-focused or existential or exposure or gestalt or implosive or insight or morita or multisystemic or music or narrative or network or person-centered or persuasion or psycholog* or psychoanalytic or psychotherapeutic processes or primal or reciprocal inhibition or reality or relationship or solution focused or virtual reality exposure) adj2 (activation or intervention* or method* or therap* or treatment* or training*)).ti,ab,kf.  42. ((acceptance or arousal or attention* or coping or incentive* or mental or motivation* or psycholog* or stimulus or volitional*) adj2 (control* or effort* or enhance* or exertion* or performance* or preparation* or reinforcement* or reward* or routin* or skill* or strateg* or techniq* or training*)).ti,ab,kf.  43. or/30-42  44. exp Athletic Performance/  45. successful performance*.ti,ab,kf.  46. ((performance or success) adj2 (athletic or competiti* or enhance* or endurance or game* or good or improve* or match* or peak or player* or physical* or outcome* or sport*)).ti,ab,kf.  47. or/44-46  48. 29 and 43 and 47  49. limit 48 to english language  50. remove duplicates from 48 | |

2. PsycInfo (EBSCO)

| Date of Search: 2022-04-13  Number of hits: 4,598  Comments: | Field labels   - DE = controlled term - / = non exploded controlled term - TI AB KW = title, abstract and author keywords - Nx = within x words, regardless of order - Wx= within x words, fixed word order   * = truncation of word for alternate endings |
| --- | --- |
| \| # \| Query \| \| --- \| --- \| \| S1 \| DE "Sports" OR DE "Adaptive Sports" OR DE "Athletic Participation" OR DE "Athletic Performance" OR DE "Baseball" OR DE "Basketball" OR DE "Cycling" OR DE "Extreme Sports" OR DE "Football" OR DE "Judo" OR DE "Martial Arts" OR DE "Professional Sports" OR DE "Soccer" OR DE "Swimming" OR DE "Tennis" OR DE "Weightlifting" \| \| S2 \| DE "Athletes" OR DE "College Athletes" OR DE "Professional Athletes" \| \| S3 \| DE "Walking" \| \| S4 \| DE "Dance" \| \| S5 \| TI ( (aikido OR alpinis* OR archer* OR athlete* OR athletics OR badminton OR ballgame* OR "ball game*" OR bandy OR baseball OR basketball OR biathl* OR bicycle OR bicycling OR billiard OR bobsleigh* OR bowling OR bowler* OR boxing OR boxer* OR canoe* OR cheerlead* OR "cheer lead*" OR cricket OR curling OR cycling OR cyclist* OR danc* OR dart OR darts OR decathl* OR discus OR diver OR divers OR diving OR dressage* OR duathl* OR eventing OR "equestrian polo" OR fencer* OR fencing OR floorball OR "floor ball" OR football OR golf* OR gymnast* OR "hammer throw*" OR handball* OR hepathl* OR hockey OR hurdler* OR "javelin throw*" OR judo OR jujitsu OR karate OR kayak* OR "kung fu" OR lacrosse OR luge OR olympic* OR orienteer* OR marathon* OR "martial art*" OR motorbike* OR motorsport* OR "mountain climb*" OR mountaineer* OR parathlet* OR "para-athlet*" OR "para athlet*" OR parasport* OR pentathl* OR "pole vault*" OR racewalk* OR "race walk*" OR racquetball OR racketball OR "racket ball" OR rally OR rider* OR rodel OR rower* OR rowing OR rugby OR running OR runner* OR sail* OR "shot put*" OR shooter* OR shooting* OR skateboard* OR skater* OR skating OR skeleton OR skier* OR ski OR skiing OR snooker* OR snowboard* OR soccer OR softball OR squash OR speedskat* OR sport* OR steeplechase* OR surfer* OR surfing OR swim* OR "tae kwon do" OR taekwondo OR tai OR thai OR taiji OR taijiquan OR taichi OR "team game*" OR tennis OR "track and field*" OR triathl* OR trampoline OR volleyball OR "water polo" OR "weight* lifting" OR "lifting weight*" OR weightlifting OR windsurf* OR wrestl* OR wushu ) ) OR AB ( (aikido OR alpinis* OR archer* OR athlete* OR athletics OR badminton OR ballgame* OR "ball game*" OR bandy OR baseball OR basketball OR biathl* OR bicycle OR bicycling OR billiard OR bobsleigh* OR bowling OR bowler* OR boxing OR boxer* OR canoe* OR cheerlead* OR "cheer lead*" OR cricket OR curling OR cycling OR cyclist* OR danc* OR dart OR darts OR decathl* OR discus OR diver OR divers OR diving OR dressage* OR duathl* OR eventing OR "equestrian polo" OR fencer* OR fencing OR floorball OR "floor ball" OR football OR golf* OR gymnast* OR "hammer throw*" OR handball* OR hepathl* OR hockey OR hurdler* OR "javelin throw*" OR judo OR jujitsu OR karate OR kayak* OR "kung fu" OR lacrosse OR luge OR olympic* OR orienteer* OR marathon* OR "martial art*" OR motorbike* OR motorsport* OR "mountain climb*" OR mountaineer* OR parathlet* OR "para-athlet*" OR "para athlet*" OR parasport* OR pentathl* OR "pole vault*" OR racewalk* OR "race walk*" OR racquetball OR racketball OR "racket ball" OR rally OR rider* OR rodel OR rower* OR rowing OR rugby OR running OR runner* OR sail* OR "shot put*" OR shooter* OR shooting* OR skateboard* OR skater* OR skating OR skeleton OR skier* OR ski OR skiing OR snooker* OR snowboard* OR soccer OR softball OR squash OR speedskat* OR sport* OR steeplechase* OR surfer* OR surfing OR swim* OR "tae kwon do" OR taekwondo OR tai OR thai OR taiji OR taijiquan OR taichi OR "team game*" OR tennis OR "track and field*" OR triathl* OR trampoline OR volleyball OR "water polo" OR "weight* lifting" OR "lifting weight*" OR weightlifting OR windsurf* OR wrestl* OR wushu ) ) OR KW ( (aikido OR alpinis* OR archer* OR athlete* OR athletics OR badminton OR ballgame* OR "ball game*" OR bandy OR baseball OR basketball OR biathl* OR bicycle OR bicycling OR billiard OR bobsleigh* OR bowling OR bowler* OR boxing OR boxer* OR canoe* OR cheerlead* OR "cheer lead*" OR cricket OR curling OR cycling OR cyclist* OR danc* OR dart OR darts OR decathl* OR discus OR diver OR divers OR diving OR dressage* OR duathl* OR eventing OR "equestrian polo" OR fencer* OR fencing OR floorball OR "floor ball" OR football OR golf* OR gymnast* OR "hammer throw*" OR handball* OR hepathl* OR hockey OR hurdler* OR "javelin throw*" OR judo OR jujitsu OR karate OR kayak* OR "kung fu" OR lacrosse OR luge OR olympic* OR orienteer* OR marathon* OR "martial art*" OR motorbike* OR motorsport* OR "mountain climb*" OR mountaineer* OR parathlet* OR "para-athlet*" OR "para athlet*" OR parasport* OR pentathl* OR "pole vault*" OR racewalk* OR "race walk*" OR racquetball OR racketball OR "racket ball" OR rally OR rider* OR rodel OR rower* OR rowing OR rugby OR running OR runner* OR sail* OR "shot put*" OR shooter* OR shooting* OR skateboard* OR skater* OR skating OR skeleton OR skier* OR ski OR skiing OR snooker* OR snowboard* OR soccer OR softball OR squash OR speedskat* OR sport* OR steeplechase* OR surfer* OR surfing OR swim* OR "tae kwon do" OR taekwondo OR tai OR thai OR taiji OR taijiquan OR taichi OR "team game*" OR tennis OR "track and field*" OR triathl* OR trampoline OR volleyball OR "water polo" OR "weight* lifting" OR "lifting weight*" OR weightlifting OR windsurf* OR wrestl* OR wushu ) ) \| \| S6 \| TI ( ((high OR long OR triple OR equestrian OR ski ) N1 jump* ) ) OR AB ( ((high OR long OR triple OR equestrian OR ski ) N1 jump* ) ) OR KW ( ((high OR long OR triple OR equestrian OR ski ) N1 jump* ) ) \| \| S7 \| S1 OR S2 OR S3 OR S4 OR S5 OR S6 \| \| S8 \| DE "Goals" OR DE "Educational Objectives" OR DE "Goal Orientation" OR DE "Goal Setting" OR DE "Organizational Objectives" \| \| S9 \| DE "Goal Setting" \| \| S10 \| DE "Goal Orientation" \| \| S11 \| DE "Relaxation" \| \| S12 \| DE "Relaxation Therapy" OR DE "Progressive Relaxation Therapy" \| \| S13 \| DE "Self-Concept" OR DE "Academic Self Concept" OR DE "Athletic Identity" OR DE "Entitlement (Psychological)" OR DE "Self-Confidence" OR DE "Self-Congruence" OR DE "Self-Esteem" OR DE "Self-Forgiveness" OR DE "Self-Regard" OR DE "Self-Worth" OR DE "Sense of Coherence" \| \| S14 \| DE "Self-Efficacy" \| \| S15 \| DE "Self-Talk" \| \| S16 \| DE "Motivation" \| \| S17 \| DE "Achievement Motivation" \| \| S18 \| DE "Extrinsic Motivation" \| \| S19 \| DE "Intrinsic Motivation" \| \| S20 \| DE "Self-Expansion" \| \| S21 \| DE "Imagery" OR DE "Conceptual Imagery" OR DE "Spatial Imagery" \| \| S22 \| DE "Guided Imagery" \| \| S23 \| DE "Cognitive Behavior Therapy" OR DE "Acceptance and Commitment Therapy" OR DE "Cognitive Processing Therapy" OR DE "Prolonged Exposure Therapy" \| \| S24 \| DE "Cognitive Techniques" OR DE "Cognitive Restructuring" OR DE "Cognitive Therapy" OR DE "Self-Instructional Training" \| \| S25 \| DE "Cognitive Restructuring" \| \| S26 \| DE "Self-Monitoring" \| \| S27 \| (DE "Self-Help Techniques" OR DE "Self-Management" OR DE "Self-Instructional Training") \| \| S28 \| (DE "Psychotherapy" OR DE "Active Listening" OR DE "Adlerian Psychotherapy" OR DE "Adolescent Psychotherapy" OR DE "Affirmative Therapy" OR DE "Age Regression (Hypnotic)" OR DE "Analytical Psychotherapy" OR DE "Animal Assisted Therapy" OR DE "Autogenic Training" OR DE "Autogenic Training" OR DE "Brief Psychotherapy" OR DE "Brief Relational Therapy" OR DE "Centering" OR DE "Cotherapy" OR DE "Child Psychotherapy" OR DE "Client Centered Therapy" OR DE "Conjoint Therapy" OR DE "Conversion Therapy" OR DE "Couples Therapy" OR DE "Dream Analysis" OR DE "Eclectic Psychotherapy" OR DE "Emotion Focused Therapy" OR DE "Empty Chair Technique" OR DE "Encounter Group Therapy" OR DE "Ericksonian Psychotherapy" OR DE "Existential Therapy" OR DE "Experiential Psychotherapy" OR DE "Expressive Psychotherapy" OR DE "Eye Movement Desensitization Therapy" OR DE "Family Therapy" OR DE "Feminist Therapy" OR DE "Geriatric Psychotherapy" OR DE "Free Association" OR DE "Gestalt Therapy" OR DE "Group Psychotherapy" OR DE "Guided Imagery" OR DE "Guided Imagery" OR DE "Integrative Psychotherapy" OR DE "Humanistic Psychotherapy" OR DE "Hypnotherapy" OR DE "Individual Psychotherapy" OR DE "Insight Therapy" OR DE "Interpersonal Psychotherapy" OR DE "Life Review" OR DE "Logotherapy" OR DE "Marathon Group Therapy" OR DE "Mirroring" OR DE "Morita Therapy" OR DE "Motivational Interviewing" OR DE "Mutual Storytelling Technique" OR DE "Multisystemic Therapy" OR DE "Narrative Therapy" OR DE "Network Therapy" OR DE "Paradoxical Techniques" OR DE "Persuasion Therapy" OR DE "Play Therapy" OR DE "Posthypnotic Suggestions" OR DE "Primal Therapy" OR DE "Psychoanalysis" OR DE "Psychodrama" OR DE "Psychodynamic Psychotherapy" OR DE "Psychotherapeutic Counseling" OR DE "Psychotherapeutic Techniques" OR DE "Rational Emotive Behavior Therapy" OR DE "Reality Therapy" OR DE "Relationship Therapy" OR DE "Schema Therapy" OR DE "Self-Analysis" OR DE "Solution Focused Therapy" OR DE "Strategic Family Therapy" OR DE "Strategic Therapy" OR DE "Structural Family Therapy" OR DE "Supportive Psychotherapy" OR DE "Therapeutic Community" OR DE "Transactional Analysis") \| \| S29 \| DE "Psychotherapeutic Processes" OR DE "Contemporaneity" OR DE "Countertransference" OR DE "Enactments" OR DE "Insight (Psychotherapeutic Process)" OR DE "Negative Therapeutic Reaction" OR DE "Psychotherapeutic Breakthrough" OR DE "Psychotherapeutic Neutrality" OR DE "Psychotherapeutic Resistance" OR DE "Psychotherapeutic Transference" OR DE "Therapeutic Alliance" \| \| S30 \| DE "Psychotherapeutic Techniques" OR DE "Active Listening" OR DE "Animal Assisted Therapy" OR DE "Autogenic Training" OR DE "Brief Relational Therapy" OR DE "Centering" OR DE "Cotherapy" OR DE "Dream Analysis" OR DE "Empty Chair Technique" OR DE "Ericksonian Psychotherapy" OR DE "Free Association" OR DE "Guided Imagery" OR DE "Life Review" OR DE "Mirroring" OR DE "Morita Therapy" OR DE "Motivational Interviewing" OR DE "Mutual Storytelling Technique" OR DE "Network Therapy" OR DE "Paradoxical Techniques" OR DE "Psychodrama" \| \| S31 \| (DE "Behavior Therapy" OR DE "Aversion Therapy" OR DE "Conversion Therapy" OR DE "Dialectical Behavior Therapy" OR DE "Exposure Therapy" OR DE "Implosive Therapy" OR DE "Reciprocal Inhibition Therapy" OR DE "Response Cost" OR DE "Systematic Desensitization Therapy" OR DE "Covert Sensitization" OR DE "Imaginal Exposure" OR DE "Implosive Therapy" OR DE "In Vivo Exposure" OR DE "Prolonged Exposure Therapy" OR DE "Systematic Desensitization Therapy" OR DE "Virtual Reality Exposure Therapy") \| \| S32 \| DE "Behavior Analysis" \| \| S33 \| DE "Biofeedback" OR DE "Biofeedback Training" OR DE "Neurotherapy" \| \| S34 \| DE "Assertiveness Training" \| \| S35 \| DE "Bibliotherapy" \| \| S36 \| (DE "Counseling" OR DE "Community Counseling" OR DE "Cross Cultural Counseling" OR DE "Educational Counseling" OR DE "Genetic Counseling" OR DE "Gerontological Counseling" OR DE "Grief Counseling" OR DE "Group Counseling" OR DE "Marriage Counseling" OR DE "Microcounseling" OR DE "Multicultural Counseling" OR DE "Occupational Guidance" OR DE "Pastoral Counseling" OR DE "Peer Counseling" OR DE "Premarital Counseling" OR DE "Psychotherapeutic Counseling" OR DE "Rehabilitation Counseling" OR DE "School Counseling" OR DE "Conjoint Therapy" OR DE "Family Therapy" OR DE "Conjoint Therapy" OR DE "Strategic Family Therapy" OR DE "Structural Family Therapy") \| \| S37 \| (DE "Hypnosis" OR DE "Age Regression (Hypnotic)" OR DE "Autohypnosis" OR DE "Hypnotic Susceptibility" OR DE "Hypnotists" OR DE "Posthypnotic Suggestions" OR DE "Hypnotherapists") \| \| S38 \| DE "Meditation" \| \| S39 \| DE "Mindfulness" \| \| S40 \| DE "Yoga" \| \| S41 \| DE "Music Therapy" \| \| S42 \| TI ( ("acceptance-commitment" OR "acceptance and commitment therap*" OR achievement* OR "active listening" OR "activation regulation" OR "applied sport psycholog*" OR "applied behavior analys*" OR biofeedback OR bibliotherap* OR "breathing exercise*" OR "cognitive remediation" OR counsel?ing OR cotherap* OR desensitization OR "directed reverie therap*" OR "dream analy*" OR flow OR "goal motivation*" OR "goal set*" OR "goal striv*" OR guidance OR hypnotherap* OR hypnosis OR imagery OR logotherap* OR mac OR meditation OR meditating OR mindful* OR mirroring OR "motivational interview*" OR "mutual storytelling" OR "paradoxical technique*" OR pettlep OR psychoanaly* OR psychodrama* OR psychodynam* OR "psychological performance enhancement techniq*" OR pet OR "preperformance routine*" OR "pre performance routine*" OR psychotherap* OR qigong OR "qi gong" OR relaxation OR "tai ji" OR "therapeutic alliance*" OR "therapeutic communit*" OR "thought stopping" OR "transactional analy*" OR visualisation OR visualization OR yoga ) ) OR AB ( ("acceptance-commitment" OR "acceptance and commitment therap*" OR achievement* OR "active listening" OR "activation regulation" OR "applied sport psycholog*" OR "applied behavior analys*" OR biofeedback OR bibliotherap* OR "breathing exercise*" OR "cognitive remediation" OR counsel?ing OR cotherap* OR desensitization OR "directed reverie therap*" OR "dream analy*" OR flow OR "goal motivation*" OR "goal set*" OR "goal striv*" OR guidance OR hypnotherap* OR hypnosis OR imagery OR logotherap* OR mac OR meditation OR meditating OR mindful* OR mirroring OR "motivational interview*" OR "mutual storytelling" OR "paradoxical technique*" OR pettlep OR psychoanaly* OR psychodrama* OR psychodynam* OR "psychological performance enhancement techniq*" OR pet OR "preperformance routine*" OR "pre performance routine*" OR psychotherap* OR qigong OR "qi gong" OR relaxation OR "tai ji" OR "therapeutic alliance*" OR "therapeutic communit*" OR "thought stopping" OR "transactional analy*" OR visualisation OR visualization OR yoga ) ) OR KW ( ("acceptance-commitment" OR "acceptance and commitment therap*" OR achievement* OR "active listening" OR "activation regulation" OR "applied sport psycholog*" OR "applied behavior analys*" OR biofeedback OR bibliotherap* OR "breathing exercise*" OR "cognitive remediation" OR counsel?ing OR cotherap* OR desensitization OR "directed reverie therap*" OR "dream analy*" OR flow OR "goal motivation*" OR "goal set*" OR "goal striv*" OR guidance OR hypnotherap* OR hypnosis OR imagery OR logotherap* OR mac OR meditation OR meditating OR mindful* OR mirroring OR "motivational interview*" OR "mutual storytelling" OR "paradoxical technique*" OR pettlep OR psychoanaly* OR psychodrama* OR psychodynam* OR "psychological performance enhancement techniq*" OR pet OR "preperformance routine*" OR "pre performance routine*" OR psychotherap* OR qigong OR "qi gong" OR relaxation OR "tai ji" OR "therapeutic alliance*" OR "therapeutic communit*" OR "thought stopping" OR "transactional analy*" OR visualisation OR visualization OR yoga ) ) \| \| S43 \| TI ( ("self belief*" OR "self concept" OR "self confidence*" OR "self determination" OR "self doubt*" OR "self efficac*" OR "self esteem" OR "self instructional" OR "self monitoring" OR "self perception*" OR "self talk*" ) ) OR AB ( ("self belief*" OR "self concept" OR "self confidence*" OR "self determination" OR "self doubt*" OR "self efficac*" OR "self esteem" OR "self instructional" OR "self monitoring" OR "self perception*" OR "self talk*" ) ) OR KW ( ("self belief*" OR "self concept" OR "self confidence*" OR "self determination" OR "self doubt*" OR "self efficac*" OR "self esteem" OR "self instructional" OR "self monitoring" OR "self perception*" OR "self talk*" ) ) \| \| S44 \| TI ( ("cognitive program*" OR "cognitive psychotherap*" OR "cognitive restructuring*" OR "cognitive strateg*" OR "cognitive therap*" OR "cognitive train*" OR "cognitive behavio* program*" OR "cognitive behavio* psychotherap*" OR "cognitive behavio* restructuring*" OR "cognitive behavio* strateg*" OR "cognitive behavio* train*" OR "cognitive skill* training" OR "cognitive skill* therap*" ) ) OR AB ( ("cognitive program*" OR "cognitive psychotherap*" OR "cognitive restructuring*" OR "cognitive strateg*" OR "cognitive therap*" OR "cognitive train*" OR "cognitive behavio* program*" OR "cognitive behavio* psychotherap*" OR "cognitive behavio* restructuring*" OR "cognitive behavio* strateg*" OR "cognitive behavio* train*" OR "cognitive skill* training" OR "cognitive skill* therap*" ) ) OR KW ( ("cognitive program*" OR "cognitive psychotherap*" OR "cognitive restructuring*" OR "cognitive strateg*" OR "cognitive therap*" OR "cognitive train*" OR "cognitive behavio* program*" OR "cognitive behavio* psychotherap*" OR "cognitive behavio* restructuring*" OR "cognitive behavio* strateg*" OR "cognitive behavio* train*" OR "cognitive skill* training" OR "cognitive skill* therap*" ) ) \| \| S45 \| TI ( ((acceptance OR adlerian OR "anger management" OR aversive OR aversion OR autogenic OR behavio* OR brief OR "client centered" OR commitment OR conversion OR emotion* OR "emotion-focused" OR existential OR exposure OR gestalt OR implosive OR insight OR morita OR multisystemic OR music OR narrative OR network OR "person-centered" OR persuasion OR psycholog* OR psychoanalytic OR "psychotherapeutic processes" OR primal OR "reciprocal inhibition" OR reality OR relationship OR "solution focused" OR "virtual reality exposure" ) N1 (activation OR intervention* OR method* OR therap* OR treatment* OR training* )) ) OR AB ( ((acceptance OR adlerian OR "anger management" OR aversive OR aversion OR autogenic OR behavio* OR brief OR "client centered" OR commitment OR conversion OR emotion* OR "emotion-focused" OR existential OR exposure OR gestalt OR implosive OR insight OR morita OR multisystemic OR music OR narrative OR network OR "person-centered" OR persuasion OR psycholog* OR psychoanalytic OR "psychotherapeutic processes" OR primal OR "reciprocal inhibition" OR reality OR relationship OR "solution focused" OR "virtual reality exposure" ) N1 (activation OR intervention* OR method* OR therap* OR treatment* OR training* )) ) OR KW ( ((acceptance OR adlerian OR "anger management" OR aversive OR aversion OR autogenic OR behavio* OR brief OR "client centered" OR commitment OR conversion OR emotion* OR "emotion-focused" OR existential OR exposure OR gestalt OR implosive OR insight OR morita OR multisystemic OR music OR narrative OR network OR "person-centered" OR persuasion OR psycholog* OR psychoanalytic OR "psychotherapeutic processes" OR primal OR "reciprocal inhibition" OR reality OR relationship OR "solution focused" OR "virtual reality exposure" ) N1 (activation OR intervention* OR method* OR therap* OR treatment* OR training* )) ) \| \| S46 \| TI ( ((acceptance OR arousal OR attention* OR coping OR incentive* OR mental OR motivation* OR psycholog* OR stimulus OR volitional* ) N1 (control* OR effort* OR enhance* OR exertion* OR performance* OR preparation* OR reinforcement* OR reward* OR routin* OR skill* OR strateg* OR techniq* OR training* )) ) OR AB ( ((acceptance OR arousal OR attention* OR coping OR incentive* OR mental OR motivation* OR psycholog* OR stimulus OR volitional* ) N1 (control* OR effort* OR enhance* OR exertion* OR performance* OR preparation* OR reinforcement* OR reward* OR routin* OR skill* OR strateg* OR techniq* OR training* )) ) OR KW ( ((acceptance OR arousal OR attention* OR coping OR incentive* OR mental OR motivation* OR psycholog* OR stimulus OR volitional* ) N1 (control* OR effort* OR enhance* OR exertion* OR performance* OR preparation* OR reinforcement* OR reward* OR routin* OR skill* OR strateg* OR techniq* OR training* )) ) \| \| S47 \| S8 OR S9 OR S10 OR S11 OR S12 OR S13 OR S14 OR S15 OR S16 OR S17 OR S18 OR S19 OR S20 OR S21 OR S22 OR S23 OR S24 OR S25 OR S26 OR S27 OR S28 OR S29 OR S30 OR S31 OR S32 OR S33 OR S34 OR S35 OR S36 OR S37 OR S38 OR S39 OR S40 OR S41 OR S42 OR S43 OR S44 OR S45 OR S46 \| \| S48 \| DE "Performance" \| \| S49 \| DE "Athletic Performance" \| \| S50 \| DE "Group Performance" \| \| S51 \| TI "successful performance*" OR AB "successful performance*" OR KW "successful performance*" \| \| S52 \| TI ( ((performance OR success ) N1 (athletic OR competiti* OR enhance* OR endurance OR game* OR good OR improve* OR match* OR peak OR player* OR physical* OR outcome* OR sport* )) ) OR AB ( ((performance OR success ) N1 (athletic OR competiti* OR enhance* OR endurance OR game* OR good OR improve* OR match* OR peak OR player* OR physical* OR outcome* OR sport* )) ) OR KW ( ((performance OR success ) N1 (athletic OR competiti* OR enhance* OR endurance OR game* OR good OR improve* OR match* OR peak OR player* OR physical* OR outcome* OR sport* )) ) \| \| S53 \| S48 OR S49 OR S50 OR S51 OR S52 \| \| S54 \| S7 AND S47 AND S53 \| \| S55 \| S7 AND S47 AND S53 - Narrow by Language: - english \| | |

3. Web of Science

| Date of Search: 2022-04-13  Number of hits: 5,270  Comments: Editions = A&HCI , ESCI , SCI-EXPANDED , SSCI | Field labels: TOPIC = title, abstract, keyword NEAR/x = within x number of words |
| --- | --- |
| # 1 TOPIC: (aikido or alpinis* or archer* or athlete* or athletics or badminton or ballgame* or "ball game*" or bandy or baseball or basketball or biathl* or bicycle or bicycling or billiard or bobsleigh* or bowling or bowler* or "boxing" or "boxer*" or canoe* or cheerlead* or "cheer lead*" or cricket or "curling" or "cycling" or "cyclist*" or "danc*" or dart or darts or decathl* or "discus" or "diver" or "divers" or "diving" or "dressage*" or "duathl*" or "eventing" or "equestrian polo" or fencer* or fencing or floorball or "floor ball" or football or golf* or "gymnast*" or "hammer throw*" or handball* or hepathl* or hockey or "hurdler*" or "javelin throw*" or judo or jujitsu or karate or kayak* or "kung fu" or lacrosse or "luge" or "olympic*" or "orienteer*" or "marathon*" or "martial art*" or motorbike* or motorsport* or "mountain climb*" or "mountaineer*" or "parathlet*" or "para-athlet*" or "para athlet*" or "parasport*" or pentathl* or "pole vault*" or racewalk* or "race walk*" or racquetball or racketball or "racket ball" or rally or rider* or rodel or "rower*" or "rowing" or rugby or "running" or "runner*" or "sail*" or "shot put*" or "shooter*" or "shooting*" or skateboard* or "skater*" or "skating" or skeleton or "skier*" or "ski" or "skiing" or snooker* or snowboard* or soccer or softball or squash or speedskat* or "sport*" or steeplechase* or surfer* or surfing or "swim*" or "tae kwon do" or taekwondo or tai or thai or taiji or taijiquan or taichi or "team game*" or tennis or "track and field*" or triathl* or trampoline or volleyball or "water polo" or "weight* lifting" or "lifting weight*" or weightlifting or windsurf* or wrestl* or wushu)  OR  TOPIC: (((high or long or triple or equestrian or ski) NEAR/1 jump*))  #2 TOPIC: ("acceptance-commitment" or "acceptance and commitment therap*" or achievement* or "active listening" or "activation regulation" or "applied sport psycholog*" or "applied behavior analys*" or biofeedback or bibliotherap* or "breathing exercise*" or "cognitive remediation" or counsel?ing or cotherap* or desensitization or "directed reverie therap*" or "dream analy*" or flow or "goal motivation*" or "goal set*" or "goal striv*" or guidance or hypnotherap* or hypnosis or imagery or logotherap* or mac or meditation or meditating or mindful* or mirroring or "motivational interview*" or "mutual storytelling" or "paradoxical technique*" or pettlep or psychoanaly* or psychodrama* or psychodynam* or "psychological performance enhancement techniq*" or pet or "preperformance routine*" or "pre performance routine*" or psychotherap* or qigong or "qi gong" or relaxation or "therapeutic alliance*" or "therapeutic communit*" or "thought stopping" or "transactional analy*" or visualisation or visualization or yoga or "self belief*" or "self concept*" or "self confidence*" or "self determination" or "self doubt*" or "self efficac*" or "self esteem" or "self instructional" or "self monitoring" or "self perception*" or "self talk*" or "cognitive program*" or "cognitive psychotherap*" or "cognitive restructuring*" or "cognitive strateg*" or "cognitive therap*" or "cognitive train*"or "cognitive behavio* program*" or "cognitive behavio* psychotherap*" or "cognitive behavio* restructuring*" or "cognitive behavio* strateg*" or "cognitive behavio* therap*" or "cognitive behavio* train*" or "cognitive skill* training" or "cognitive skill* therap*")  OR  TOPIC: ((acceptance or adlerian or "anger management" or aversive or aversion or autogenic or behavio* or brief or "client centered" or commitment or conversion or emotion* or "emotion-focused" or existential or exposure or gestalt or implosive or insight or morita or multisystemic or music or narrative or network or "person-centered" or persuasion or psycholog* or psychoanalytic or "psychotherapeutic processes" or primal or "reciprocal inhibition" or reality or relationship or "solution focused" or "virtual reality exposure") NEAR/2 (activation or intervention* or method* or therap* or treatment* or training*))  OR  TOPIC: ((acceptance or arousal or attention* or coping or incentive* or mental or motivation* or psycholog* or stimulus or volitional*) NEAR/2 (control* or effort* or enhance* or exertion* or performance* or preparation* or reinforcement* or reward* or routin* or skill* or strateg* or techniq* or training*))  # 3 TOPIC=(("successful performance*"))  OR  TOPIC=((performance or success) NEAR/2 ("athletic" or competiti* or enhance* or endurance or game* or "good" or improve* or match* or "peak" or player* or physical* or outcome* or sport*))  #4 #1 AND #2 AND #3  #4 AND LANGUAGE: (English) | |

4. SPORTDiscus (Ebsco)

| Date of Search: 2022-04-13  Number of hits: 3,884  Comments: | Field labels: DE = controlled term TI = title AB = abstract Nx = within x words |
| --- | --- |
| S1 DE "SPORTS"  S2 DE "AQUATIC sports" OR DE "AQUATIC sports competitions" OR DE "BOAT jousting" OR DE "CANOE polo" OR DE "CANOES & canoeing" OR DE "DEEP diving" OR DE "DIVING" OR DE "DRAGON boat racing" OR DE "ICE diving" OR DE "KNEEBOARDING" OR DE "LOGROLLING (Aquatic sports)" OR DE "MIDDLE distance swimming" OR DE "MONO water skiing" OR DE "PADDLEBOARDING" OR DE "PARASKIING" OR DE "RAFTING (Sports)" OR DE "REGATTAS" OR DE "ROWING" OR DE "SAILBOAT racing" OR DE "SAILING)" OR DE "SURFING" OR DE "SWIMMING" OR DE "TUBING (Aquatic sports)" OR DE "WAKEBOARDING" OR DE "WATER polo" OR DE "WATER skiing" OR DE "WHITEWATER kayaking" OR DE "WHITEWATER rafting"  S3 DE "BALL games" OR DE "ANETSO" OR DE "BALL hockey" OR DE "BALLE au tamis (Game)" OR DE "BASEBALL" OR DE "BASKETBALL" OR DE "BATTLE ball" OR DE "BICYCLE polo" OR DE "BILLIARDS" OR DE "BOWLING games" OR DE "BROOMBALL" OR DE "CAMOGIE (Game)" OR DE "CRICKET (Sport)" OR DE "CROQUET" OR DE "DODGEBALL" OR DE "FIELD hockey" OR DE "FLICKERBALL" OR DE "FOOTBALL" OR DE "GOALBALL" OR DE "GOLF" OR DE "GOLF croquet" OR DE "HANDBALL" OR DE "HURLING (Game)" OR DE "INDOOR hockey" OR DE "JAPANESE polo" OR DE "JIAN zi (Game)" OR DE "KANG (Game)" OR DE "KICKBALL" OR DE "LACROSSE" OR DE "LAPTA (Game)" OR DE "LAWN tempest (Game)" OR DE "MINTON (Game)" OR DE "PARLOR football" OR DE "PARLOR tennis" OR DE "PICKLEBALL (Game)" OR DE "PIZE-ball" OR DE "POLO" OR DE "POLOCROSSE" OR DE "PUSH ball" OR DE "QUIDDITCH (Game)"  S4 DE "RACQUETBALL" OR DE "RAGA (Game)" OR DE "ROLL ball" OR DE "ROUNDERS" OR DE "RUGBALL" OR DE "SCHLAGBALL" OR DE "SHINTY (Game)" OR DE "SOCCER" OR DE "SOFTBALL" OR DE "SPEED-a-way (Game)" OR DE "SPEEDBALL" OR DE "STICKBALL (Urban game)" OR DE "STOOLBALL" OR DE "TABLE tennis" OR DE "TCHOUKBALL" OR DE "TENNIS" OR DE "TETHERBALL" OR DE "TRAPBALL" OR DE "VOLLEYBALL" OR DE "WALLYBALL" OR DE "WATER polo" OR DE "WICKET" OR DE "WIFFLE ball" )  S5 DE "COLLEGE sports" OR DE "BCS National Championship Game (College football)" OR DE "BOWL games (College football)" OR DE "COLLEGE baseball" OR DE "COLLEGE basketball" OR DE "COLLEGE boxing" OR DE "COLLEGE cross-country running" OR DE "COLLEGE field hockey" OR DE "COLLEGE football" OR DE "COLLEGE golf" OR DE "COLLEGE hockey" OR DE "COLLEGE lacrosse" OR DE "COLLEGE rowing" OR DE "COLLEGE rugby football" OR DE "COLLEGE skiing" OR DE "COLLEGE soccer" OR DE "COLLEGE sports competitions" OR DE "COLLEGE sports for women" OR DE "COLLEGE swimming" OR DE "COLLEGE tennis" OR DE "COLLEGE track & field" OR DE "COLLEGE volleyball" OR DE "COLLEGE wrestling" OR DE "COMMUNITY college sports" OR DE "NATIONAL Invitation Tournament (Basketball)" OR DE "VAULTING"  S6 DE "CONTACT sports"  S7 DE "ENDURANCE sports" OR DE "ULTRAENDURANCE sports"  S8 DE "EXTREME sports" OR DE "EXTREME skiing" OR DE "KITE surfing" OR DE "X Games (Extreme sports)"  S9 DE "MILITARY sports"  S10 DE "MOTORSPORTS" OR DE "ALL terrain vehicle racing" OR DE "AUTOMOBILE racing" OR DE "DRIFTING (Motorsport)" OR DE "MOTORCYCLE racing" OR DE "MUD racing" OR DE "OFF-road racing"  S11 DE "GYMNASTICS" OR DE "ACROBATICS" OR DE "ARTISTIC gymnastics" OR DE "CARTWHEELS" OR DE OR DE "GYMNASTICS for men" OR DE "GYMNASTICS for people with disabilities" OR DE "GYMNASTICS for women" OR DE "HANDSPRINGS" OR DE "HANDSTANDS" OR DE "HEADSTANDS" OR DE "PYRAMIDS (Gymnastics)" OR DE "SOMERSAULTS" OR DE "SWEDISH gymnastics" OR DE "SWIMNASTICS" OR DE "TEAM aerobics" OR DE "TRAMPOLINES" OR DE "TUMBLING"  S12 DE "INDIVIDUAL sports  S13 DE "PROFESSIONAL sports" OR DE "PROFESSIONAL baseball" OR DE "PROFESSIONAL basketball" OR DE "PROFESSIONAL boxing" OR DE "PROFESSIONAL football" OR DE "PROFESSIONAL golf" OR DE "PROFESSIONAL hockey" OR DE "PROFESSIONAL rugby football" OR DE "PROFESSIONAL skating" OR DE "PROFESSIONAL soccer" OR DE "PROFESSIONAL sports for women" OR DE "PROFESSIONAL tennis" OR DE "PROFESSIONAL volleyball"  S14 DE "RACKET games" OR DE "BADMINTON (Game)" OR DE "BASQUE pelota (Game)" OR DE "BATTLEDORE & shuttlecock" OR DE "COURT tennis" OR DE "MINTON (Game)" OR DE "PADDLE tennis" OR DE "PICKLEBALL (Game)" OR DE "POLOCROSSE" OR DE "RACKETS (Game)" OR DE "RACQUETBALL" OR DE "SQUASH (Game)" OR DE "SQUASH tennis" OR DE "TABLE tennis" OR DE "TENNIS"  S15 DE "ROLLER skating" OR DE "FREE skating" OR DE "IN-line skating" OR DE "MOUNTAINBOARDING" OR DE "ORIGINAL set pattern dance (Skating)" OR DE "PAIR roller skating" OR DE "ROLLER derby" OR DE "ROLLER hockey" OR DE "ROLLER polo" OR DE "SKATEBOARDING" OR DE "STREET luge racing"  S16 DE "SPORTS competitions" OR DE "AQUATIC sports competitions" OR DE "COLLEGE sports competitions" OR DE "CROSS-country running competitions" OR DE "CYCLING competitions" OR DE "GYMNASTICS competitions" OR DE "SHOOTING competitions" OR DE "TRACK & field competitions" OR DE "WHEELCHAIR sports competitions" OR DE "WINTER sports competitions"  S17 DE "SPORTS teams" OR DE "AUSTRALIAN football teams" OR DE "BASEBALL teams" OR DE "BASKETBALL teams" OR DE "COLLEGE sports teams" OR DE "CURLING teams" OR DE "EXPANSION teams" OR DE "FOOTBALL teams" OR DE "HOCKEY teams" OR DE "NATIONAL sports teams" OR DE "RUGBY football teams" OR DE "SOCCER teams" OR DE "SOFTBALL teams" OR DE "TENNIS teams"  S18 DE "TEAM sports" OR DE "BASEBALL" OR DE "BASKETBALL" OR DE "BATTLE ball" OR DE "CRICKET (Sport)" OR DE "DODGEBALL" OR DE "GOALBALL" OR DE "HOCKEY" OR DE "KICKBALL" OR DE "LACROSSE" OR DE "NATIVE American stickball" OR DE "POLO" OR DE "PUSH ball" OR DE "QUIDDITCH (Game)" OR DE "RUGBALL" OR DE "RUGBY football" OR DE "SCHLAGBALL" OR DE "SOCCER" OR DE "SOFTBALL" OR DE "SPEEDBALL" OR DE "STOOLBALL" OR DE "TCHOUKBALL" OR DE "TETHERBALL" OR DE "VOLLEYBALL" OR DE "WALLYBALL" OR DE "WIFFLE ball”  S19 DE "HORSE sports" OR DE "BUZKASHI" OR DE "CHARRERIAS" OR DE "CHUCKWAGON racing" OR DE "COURSES (Horse sports)" OR DE "CROSS-country (Horsemanship)" OR DE "DRESSAGE tests" OR DE "EVENTING (Horsemanship)" OR DE "FOX hunting" OR DE "GYMKHANAS (Horsemanship)" OR DE "HORSE agility trials" OR DE "HORSE archery" OR DE "HORSE racing" OR DE "HUNT riding" OR DE "HUNTER trials (Horsemanship)" OR DE "JAPANESE polo" OR DE "JOUSTING" OR DE "POLO" OR DE "POLOCROSSE" OR DE "RIDE & tie racing" OR DE "RING jousting" OR DE "RODEOS" OR DE "SHOW driving of horse-drawn vehicles" OR DE "SHOW jumping" OR DE "SHOW riding" OR DE "TRAIL riding competitions" OR DE "TRICK riding" OR DE "VAULTING (Horsemanship)" OR DE "VERSATILITY ranch horse competitions" OR DE "WOMEN in horse sports" OR DE "WORLD Cup (Dressage)" OR DE "WORLD Cup (Show jumping)"  S20 DE "ATHLETES" OR DE "ABORIGINAL Australian athletes" OR DE "AFRICAN athletes" OR DE "AMATEUR athletes" OR DE "ARAB athletes" OR DE "ARCHERS" OR DE "ASIAN athletes" OR DE "ATHLETES as actors" OR DE "ATHLETES in art" OR DE "ATHLETES with disabilities" OR DE "BADMINTON players" OR DE "BASEBALL players" OR DE "BASKETBALL players" OR DE "BLACK athletes" OR DE "BOBSLEDDERS" OR DE "BODYBUILDERS" OR DE "BOWLERS" OR DE "BOXERS (Sports)" OR DE "CANADIAN athletes" OR DE "CANOEISTS" OR DE "CELEBRITY athletes" OR DE "CHRISTIAN athletes" OR DE "COLLEGE athletes" OR DE "CONCENTRATION camp inmates as athletes" OR DE "CRICKET players" OR DE "CROQUET players" OR DE "CURLERS (Athletes)" OR DE "CYCLISTS" OR DE "DEFENSIVE players" OR DE "DIABETIC athletes" OR DE "DIRTBOARDERS" OR DE "ELITE athletes" OR DE "ENDURANCE athletes" OR DE "EUROPEAN athletes" OR DE "FENCERS" OR DE "FOOTBALL players" OR DE "GAY athletes" OR DE "GLADIATORS" OR DE "GOLFERS" OR DE "GYMNASTS" OR DE "HANDBALL players" OR DE "HIGH school athletes" OR DE "HOCKEY players" OR DE "INTERSEX athletes" OR DE "JAI alai players" OR DE "JEWISH athletes" OR DE "JUNIOR high school athletes" OR DE "KABADDI players" OR DE "LACROSSE players" OR DE "LAWN bowlers" OR DE "LGBT athletes" OR DE "LONG-term athlete development" OR DE "MALE athletes" OR DE "MARTIAL artists" OR DE "MEXICAN athletes" OR DE "MIDDLE school athletes" OR DE "MOUNTAINEERS" OR DE "MUSLIM athletes" OR DE "NATIVE American athletes" OR DE "NETBALL players" OR DE "OFFENSIVE players" OR DE "OLDER athletes" OR DE "OLYMPIC athletes" OR DE "ORIENTEERS" OR DE "PACIFIC Islander athletes" OR DE "PROFESSIONAL athletes" OR DE "ROWERS" OR DE "RUGBY football players" OR DE "RUNNERS (Sports)" OR DE "SKATERS" OR DE "SKIERS" OR DE "SKYDIVERS" OR DE "SNOWBOARDERS" OR DE "SOCCER players" OR DE "SOFTBALL players" OR DE "SQUASH players" OR DE "STARTING players" OR DE "SUBSTITUTE players" OR DE "SURFERS" OR DE "SWIMMERS" OR DE "TABLE tennis players" OR DE "TEAM handball players" OR DE "TENNIS players" OR DE "TRACK & field athletes" OR DE "TRIATHLETES" OR DE "VOLLEYBALL players" OR DE "WATER polo players" OR DE "WEIGHT lifters" OR DE "WINDSURFERS (Persons)" OR DE "WOMEN athletes" OR DE "WRESTLERS"  S21 DE "ATHLETICS" OR DE "AMATEUR sports" OR DE "ATHLETIC tryouts" OR DE "BAG punching" OR DE "BOXING" OR DE "COLLEGE sports" OR DE "DUATHLON" OR DE "FENCING" OR DE "GYMNASTICS" OR DE "HIGHLAND games" OR DE "JIU-jitsu" OR DE "MIXED martial arts" OR DE "PANCRATIUM" OR DE "PARKOUR" OR DE "POWERLIFTING" OR DE "SENIOR Olympics" OR DE "SKATING" OR DE "SWIMMING" OR DE "TETRATHLON" OR DE "TRACK & field" OR DE "TRIATHLON" OR DE "WALKING" OR DE "WEIGHT lifting" OR DE "WRESTLING"  S22 DE "WINTER sports" OR DE "AUTOMOBILE racing on ice" OR DE "BANDY (Winter sport)" OR DE "BIATHLON" OR DE "BOBSLEDDING" OR DE "BROOMBALL" OR DE "COASTING (Winter sports)" OR DE "CURLING" OR DE "FREESTYLE skiing competitions" OR DE "HOCKEY" OR DE "ICE boating" OR DE "ICEBOATING" OR DE "MOTORCYCLE racing on ice" OR DE "NORDIC combined" OR DE "OLYMPIC Winter Games" OR DE "PARASKIING" OR DE "POLO on skates" OR DE "SKATE sailing" OR DE "SKATING" OR DE "SKIBOARDING" OR DE "SKIS & skiing" OR DE "SLED dog racing" OR DE "SLEDDING" OR DE "SLEIGHING" OR DE "SNOW golf" OR DE "SNOW skating" OR DE "SNOWBOARDING" OR DE "SNOWMOBILING" OR DE "SNOWSHOES & snowshoeing" OR DE "TOBOGGANING"  S23 DE "DANCE" OR DE "AERIAL dance" OR DE "AEROBIC dancing" OR DE "BALLET" OR DE "BALLROOM dancing" OR DE "BELLY dance" OR DE "BREAK dancing" OR DE "CHA-cha (Dance)" OR DE "COUNTRY dancing" OR DE "DANCE for people with disabilities" OR DE "FLAMENCO" OR DE "FOLK dancing" OR DE "FREE skating" OR DE "HIP-hop dance" OR DE "ICE dancing" OR DE "JAZZ dance" OR DE "LINE dancing" OR DE "LION dance" OR DE "MODERN dance" OR DE "MOVEMENT notation" OR DE "ORIGINAL set pattern dance (Skating)" OR DE "POLE dancing" OR DE "ROUND dancing" OR DE "SALSA (Dance)" OR DE "SHISHIMAI (Dance)" OR DE "STEP dancing" OR DE "TANGO (Dance)" OR DE "TAP dancing"  S24 DE "RUNNING" OR DE "BAREFOOT running" OR DE "CROSS-country running" OR DE "LONG-distance running" OR DE "MIDDLE distance running" OR DE "MINIMALIST running" OR DE "ROAD running" OR DE "RUNNING for people with disabilities" OR DE "RUNNING for women" OR DE "SPRINTING" OR DE "STREAKERS & streaking" OR DE "TRAIL running"  S25 DE "SPORTS tournaments" OR DE "KITE flying competitions" OR DE "SKIING competitions" OR DE "SOCCER tournaments" OR DE "CYCLING competitions" OR DE "GOLF tournaments"  S26 DE "CYCLING" OR DE "BICYCLE racing" OR DE "BICYCLE touring" OR DE "CYCLING competitions" OR DE "CYCLING for people with disabilities" OR DE "CYCLING for women" OR DE "MOTORCYCLING" OR DE "MOUNTAIN biking" OR DE "NIGHT cycling" OR DE "RAILBIKING"  S27 DE "MARTIAL arts" OR DE "ARCHERY" OR DE "BUDO" OR DE "DUELING" OR DE "EAST Asian martial arts" OR DE "ESCRIMA" OR DE "HAND-to-hand fighting" OR DE "JEET Kune Do" OR DE "JU-kenpo" OR DE "KAJUKENBO" OR DE "KALARIPPAYATTU" OR DE "KENJUTSU" OR DE "KENPO" OR DE "KICKBOXING" OR DE "KRAV maga" OR DE "KUN-tao" OR DE "KYUDO (Archery)" OR DE "LION dance" OR DE "MARTIAL arts for people with disabilities" OR DE "MIXED martial arts" OR DE "NINJUTSU" OR DE "PENCAK silat" OR DE "SAN-jitsu" OR DE "SHISHIMAI (Dance)" OR DE "SICKLE fighting" OR DE "SPEAR fighting"  S28 (DE "CHEERLEADING") OR  S29 DE "DARTS (Game)"  S30 DE "MOUNTAINEERING" OR DE "ALPINE scrambling" OR DE "CANYONEERING" OR DE "CRACK climbing" OR DE "FACE climbing" OR DE "MOUNTAIN running" OR DE "MOUNTAINEERING expeditions" OR DE "MOUNTAINEERING for women" OR DE "PRUSIKING" OR DE "RAPPELLING" OR DE "ROCK climbing" OR DE "SKI mountaineering" OR DE "SNOW & ice climbing"  S31 TI ( (aikido or alpinis* or archer* or athlete* or athletics or badminton or ballgame* or "ball game*" or bandy or baseball or basketball or biathl* or bicycle or bicycling or billiard or bobsleigh* or bowling or bowler* or "boxing" or "boxer*" or canoe* or cheerlead* or "cheer lead*" or cricket or "curling" or "cycling" or "cyclist*" or "danc*" or dart or darts or decathl* or "discus" or "diver" or "divers" or "diving" or "dressage*" or "duathl*" or "eventing" or "equestrian polo" or fencer* or fencing or floorball or "floor ball" or football or golf* or "gymnast*" or "hammer throw*" or handball* or hepathl* or hockey or "hurdler*" or "javelin throw*" or judo or jujitsu or karate or kayak* or "kung fu" or lacrosse or "luge" or "olympic*" or "orienteer*" or "marathon*" or "martial art*" or motorbike* or motorsport* or "mountain climb*" or "mountaineer*" or "parathlet*" or "para-athlet*" or "para athlet*" or "parasport*" or pentathl* or "pole vault*" or racewalk* or "race walk*" or racquetball or racketball or "racket ball" or rally or rider* or rodel or "rower*" or "rowing" or rugby or "running" or "runner*" or "sail*" or "shot put*" or "shooter*" or "shooting*" or skateboard* or "skater*" or "skating" or skeleton or "skier*" or "ski" or "skiing" or snooker* or snowboard* or soccer or softball or squash or speedskat* or "sport*" or steeplechase* or surfer* or surfing or "swim*" or "tae kwon do" or taekwondo or tai or thai or taiji or taijiquan or taichi or "team game*" or tennis or "track and field*" or triathl* or trampoline or volleyball or "water polo" or "weight* lifting" or "lifting weight*" or weightlifting or windsurf* or wrestl* or wushu)  S32 AB ( (aikido or alpinis* or archer* or athlete* or athletics or badminton or ballgame* or "ball game*" or bandy or baseball or basketball or biathl* or bicycle or bicycling or billiard or bobsleigh* or bowling or bowler* or "boxing" or "boxer*" or canoe* or cheerlead* or "cheer lead*" or cricket or "curling" or "cycling" or "cyclist*" or "danc*" or dart or darts or decathl* or "discus" or "diver" or "divers" or "diving" or "dressage*" or "duathl*" or "eventing" or "equestrian polo" or fencer* or fencing or floorball or "floor ball" or football or golf* or "gymnast*" or "hammer throw*" or handball* or hepathl* or hockey or "hurdler*" or "javelin throw*" or judo or jujitsu or karate or kayak* or "kung fu" or lacrosse or "luge" or "olympic*" or "orienteer*" or "marathon*" or "martial art*" or motorbike* or motorsport* or "mountain climb*" or "mountaineer*" or "parathlet*" or "para-athlet*" or "para athlet*" or "parasport*" or pentathl* or "pole vault*" or racewalk* or "race walk*" or racquetball or racketball or "racket ball" or rally or rider* or rodel or "rower*" or "rowing" or rugby or "running" or "runner*" or "sail*" or "shot put*" or "shooter*" or "shooting*" or skateboard* or "skater*" or "skating" or skeleton or "skier*" or "ski" or "skiing" or snooker* or snowboard* or soccer or softball or squash or speedskat* or "sport*" or steeplechase* or surfer* or surfing or "swim*" or "tae kwon do" or taekwondo or tai or thai or taiji or taijiquan or taichi or "team game*" or tennis or "track and field*" or triathl* or trampoline or volleyball or "water polo" or "weight* lifting" or "lifting weight*" or weightlifting or windsurf* or wrestl* or wushu) )  S33 TI ( (((high or long or triple or equestrian or ski) N1 jump*)) ) OR AB ( (((high or long or triple or equestrian or ski) N1 jump*)))  S34 S1 OR S2 OR S3 OR S4 Or S5 Or S6 OR S7 OR S8 OR S9 Or S10 OR S11 OR S12 OR S13 OR S14 OR S15 OR S16 OR S17 OR S18 OR S19 OR S20 OR S21 OR S22 OR S23 Or S24 OR S25 OR S26 OR S27 OR S28 OR S29 OR S30 OR S31 OR S32 OR S33  S35 DE "TARGETS (Sports)" OR DE "MOTIVATION (Psychology)" OR DE "ACHIEVEMENT motivation" OR DE "BURNOUT (Psychology)" OR DE "COMPETITION (Psychology)" OR DE "GOAL (Psychology)" OR DE "GROUP facilitation (Psychology)" OR DE "INTRINSIC motivation" OR DE "LEVEL of aspiration" OR DE "MOTIVATION in education" OR DE "MOTIVATIONAL interviewing"   \|  \| S36 DE "RELAXATION therapy" \| \| --- \| --- \|   S37 DE "SELF-perception" OR DE "SELF-esteem" OR DE "CONFIDENCE"  S38 DE "SELF-efficacy"  S39 DE "IMAGERY (Psychology)" OR DE "MOTOR imagery (Cognition)"  S40 DE "MENTAL training" OR DE "VISUALIZATION"  S41 DE "YOGA" OR DE "ASTANGA yoga" OR DE "CHAKRAS" OR DE "HATHA yoga" OR DE "KUNDALINI yoga" OR DE "MUSIC for yoga" OR DE "NAAM yoga" OR DE "SIDDHA yoga (Service mark)" OR DE "YIN yoga" OR DE "YOGA for people with disabilities"  S42 DE "PSYCHOTHERAPY" OR DE "BIOFEEDBACK training" OR DE "COGNITIVE therapy  S43 DE "COUNSELING" OR DE "MENTORING" OR DE "MOTIVATIONAL interviewing"  S44 TI ((acceptance or adlerian or "anger management" or aversive or aversion or autogenic or behavio* or brief or "client centered" or commitment or conversion or emotion* or "emotion-focused" or existential or exposure or gestalt or implosive or insight or morita or multisystemic or music or narrative or network or "person-centered" or persuasion or psycholog* or psychoanalytic or "psychotherapeutic processes" or primal or "reciprocal inhibition" or reality or relationship or "solution focused" or "virtual reality exposure") N2 (activation or intervention* or method* or therap* or treatment* or training*)) OR AB ((acceptance or adlerian or "anger management" or aversive or aversion or autogenic or behavio* or brief or "client centered" or commitment or conversion or emotion* or "emotion-focused" or existential or exposure or gestalt or implosive or insight or morita or multisystemic or music or narrative or network or "person-centered" or persuasion or psycholog* or psychoanalytic or "psychotherapeutic processes" or primal or "reciprocal inhibition" or reality or relationship or "solution focused" or "virtual reality exposure") N2 (activation or intervention* or method* or therap* or treatment* or training*))  S45 TI ("acceptance-commitment" or "acceptance and commitment therap*" or achievement* or "active listening" or "activation regulation" or "applied sport psycholog*" or "applied behavior analys*" or biofeedback or bibliotherap* or "breathing exercise*" or "cognitive remediation" or counsel?ing or cotherap* or desensitization or "directed reverie therap*" or "dream analy*" or flow or "goal motivation*" or "goal set*" or "goal striv*" or guidance or hypnotherap* or hypnosis or imagery or logotherap* or mac or meditation or meditating or mindful* or mirroring or "motivational interview*" or "mutual storytelling" or "paradoxical technique*" or pettlep or psychoanaly* or psychodrama* or psychodynam* or "psychological performance enhancement techniq*" or pet or "preperformance routine*" or "pre performance routine*" or psychotherap* or qigong or "qi gong" or relaxation or "therapeutic alliance*" or "therapeutic communit*" or "thought stopping" or "transactional analy*" or visualisation or visualization or yoga or "self belief*" or "self concept*" or "self confidence*" or "self determination" or "self doubt*" or "self efficac*" or "self esteem" or "self instructional" or "self monitoring" or "self perception*" or "self talk*" or "cognitive program*" or "cognitive psychotherap*" or "cognitive restructuring*" or "cognitive strateg*" or "cognitive therap*" or "cognitive train*"or "cognitive behavio* program*" or "cognitive behavio* psychotherap*" or "cognitive behavio* restructuring*" or "cognitive behavio* strateg*" or "cognitive behavio* therap*" or "cognitive behavio* train*" or "cognitive skill* training" or "cognitive skill* therap*")  S46 AB ("acceptance-commitment" or "acceptance and commitment therap*" or achievement* or "active listening" or "activation regulation" or "applied sport psycholog*" or "applied behavior analys*" or biofeedback or bibliotherap* or "breathing exercise*" or "cognitive remediation" or counsel?ing or cotherap* or desensitization or "directed reverie therap*" or "dream analy*" or flow or "goal motivation*" or "goal set*" or "goal striv*" or guidance or hypnotherap* or hypnosis or imagery or logotherap* or mac or meditation or meditating or mindful* or mirroring or "motivational interview*" or "mutual storytelling" or "paradoxical technique*" or pettlep or psychoanaly* or psychodrama* or psychodynam* or "psychological performance enhancement techniq*" or pet or "preperformance routine*" or "pre performance routine*" or psychotherap* or qigong or "qi gong" or relaxation or "therapeutic alliance*" or "therapeutic communit*" or "thought stopping" or "transactional analy*" or visualisation or visualization or yoga or "self belief*" or "self concept*" or "self confidence*" or "self determination" or "self doubt*" or "self efficac*" or "self esteem" or "self instructional" or "self monitoring" or "self perception*" or "self talk*" or "cognitive program*" or "cognitive psychotherap*" or "cognitive restructuring*" or "cognitive strateg*" or "cognitive thera(p*" or "cognitive train*"or "cognitive behavio* program*" or "cognitive behavio* psychotherap*" or "cognitive behavio* restructuring*" or "cognitive behavio* strateg*" or "cognitive behavio* therap*" or "cognitive behavio* train*" or "cognitive skill* training" or "cognitive skill* therap*")  S47 TI (acceptance or arousal or attention* or coping or incentive* or mental or motivation* or psycholog* or stimulus or volitional*) N2 (control* or effort* or enhance* or exertion* or performance* or preparation* or reinforcement* or reward* or routin* or skill* or strateg* or techniq* or training*)) OR AB ((acceptance or arousal or attention* or coping or incentive* or mental or motivation* or psycholog* or stimulus or volitional*) N2 (control* or effort* or enhance* or exertion* or performance* or preparation* or reinforcement* or reward* or routin* or skill* or strateg* or techniq* or training*))  S48 S35 OR S36 OR S37 OR S38 OR S39 OR S40 OR S41 OR S42 OR S43 OR S44 OR S45 OR S46 OR S47  S49 DE "SUCCESS"  S50 DE "Performance"  S51 TI("successful performance*") OR AB("successful performance*")  S52 TI((performance or success) N2 ("athletic" or competiti* or enhance* or endurance or game* or "good" or improve* or match* or "peak" or player* or physical* or outcome* or sport*)) OR AB((performance or success) N2 ("athletic" or competiti* or enhance* or endurance or game* or "good" or improve* or match* or "peak" or player* or physical* or outcome)  S53 S49 OR S50 OR S51 OR S52  S54 S34 AND S48 AND S53  S55 Limit S53 to Academic journals: Dissertations | |
