# Supplementary material for: Effects of Psychological Interventions to Enhance Athletic Performance: A Systematic Review and Meta-Analysis
Source: Sports Med. 2023 Oct 9;54(2):347–73. doi: 10.1007/s40279-023-01931-z (PMC10933186; doi:10.1007/s40279-023-01931-z)
Supplement: Supplementary file 2 — Supplementary file2 (DOCX 230 KB) [file 40279_2023_1931_MOESM2_ESM.docx]

**Supplementary information. Online Resource 2.**

*Article:* Effects of psychological interventions to enhance athletic performance: A systematic review and meta-analysis

*Journal:* Sports Medicine

*Authors:* Gustaf Reinebo, Sven Alfonsson, Markus Jansson-Fröjmark, Alexander Rozental, Tobias Lundgren

*Corresponding author:* Gustaf Reinebo, email [gustaf.reinebo@ki.se](mailto:gustaf.reinebo@ki.se) , Centre for Psychiatry Research, Department of Clinical Neuroscience, Karolinska Institutet, & Stockholm Health Care Services, Region Stockholm, Norra stationsgatan 69, SE-113 64, Stockholm, Sweden

**Log of rejected studies**

| Article | Published in a scientific journal | Participants | Intervention ^a^ | Performance enhancement | Design | Objective outcome | Subjective outcome | Other outcome | Exclusion reasons |
| --- | --- | --- | --- | --- | --- | --- | --- | --- | --- |
| Abdelbaky (2012) Impacts of mental toughness program on 20 km race walking | 1 | 0 | 1 | 1 | 1 | 1 | 0 | 1 | Participants |
| Abdollahipour et al. (2020) Optimizing bowling performance | 1 | 0 | 1 | 1 | 1 | 1 | 0 | 0 | Participants |
| Abouzekri & Karageorghis (2010) Effects of precompetition state anxiety interventions on performance time and accuracy among amateur soccer players: Revisiting the matching hypothesis | 1 | 0 | 1 | 1 | 1 | 1 | 0 | 1 | Participants |
| Abraham et al. (2016) Motor Imagery Practice for Enhancing Eleve Performance Among Professional Dancers: A Pilot Study | 1 | 0 | 1 | 1 | 1 | 1 | 0 | 1 | Participants |
| Abraham et al. (2017) The Effect of Motor Imagery Practice on Elevé Performance in Adolescent Female Dance Students: | 1 | 0 | 1 | 1 | 1 | 1 | 0 | 1 | Participants |
| Abraham et al. (2019) Dynamic Neuro-Cognitive Imagery (DNITM) Improves Developpe Performance, Kinematics, and Mental Imagery Ability in University-Level Dance Students | 1 | 0 | 1 | 1 | 1 | 1 | 1 | 1 | Participants |
| Afrouzeh et al. (2015) Effectiveness of PETTLEP imager on performance of passing skill in volleyball | 1 | 0 | 1 | 1 | 1 | 1 | 0 | 0 | Participants |
| Aherne et al. (2011) The effect of mindfulness training on athletes' flow: An initial investigation | 1 | 1 | 1 | 1 | 1 | 0 | 0 | 1 | Outcome |
| Alder et al. (2019) The impact of physiological load on anticipation skills in badminton: From testing to training | 1 |  | 0 |  |  |  |  |  | Intervention |
| Alderman (1993) Role of Psychological Skills Training in Increasing Athletic Pain Tolerance | 1 | 0 | 1 | 1 | 1 | 0 | 0 | 1 | Participants + Outcome |
| Allen (1988) The cognitive bases of peak performance: A classroom intervention with student-athletes | 1 | 1 | 1 | 1 | 1 | 0 | 0 | 1 | Outcome |
| Allen (1998) The use of an enhanced simplified habit-reversal procedure to reduce disruptive outbursts during athletic performance | 1 | 0 | 1 | 1 | 0 | 0 | 0 | 1 | Participants + Design + Outcome |
| Allen et al. (2010) The Influence of Positive Reflection on Attributions, Emotions, and Self-Efficacy | 1 | 0 | 1 | 0 | 1 | 0 | 0 | 1 | Participants + Not performance enhancement |
| Altfeld et al. (2017) Measuring the effectiveness of psychologically oriented basketball drills in team practice to improve self-regulation. | 1 | 0 | 1 | 1 | 1 | 0 | 0 | 1 | Participants + Outcome |
| Ampongan & Pieter (2005) Competition Anxiety In Elite Filipino Taekwondo Athletes | 1 |  | 0 |  |  |  |  |  | Intervention |
| Anderson & Campbell (2015) Accelerating skill acquisition in rowing using self-based observational learning and expert modelling during performance | 1 |  | 0 |  |  |  |  |  | Intervention |
| Anderson & Kirkpatrick (2002) Variable effects of a behavioral treatment package on the performance of inline roller speed skaters | 1 | 0 | 1 | 1 | 0 | 1 | 0 | 0 | Participants + Design |
| Andre & Means (1986) Rate of imagery in mental practice: An experimental investigation | 1 | 0 | 1 | 1 | 1 | 1 | 0 | 1 | Participants |
| Annesi (1997) The effects of long-term goal setting, short-term goal setting, and extrinsic reinfrocement on physical conditioning tasks in a tennis practice environment | 1 | 0 | 1 | 1 | 1 | 0 | 0 | 1 | Participants + Outcome |
| Annesi (1998) Applications of the individual zones of optimal functioning model for the multimodal treatment of precompetitive anxiety | 1 | exp2: 0 | exp1: 0; exp2: 1 | exp2: 1 | exp2: 0 | exp2: 0 | exp2: 1 | exp2: 1 | exp1: Intervention; exp2: Participants + Design |
| Arathoon & Malouff (2004) The Effectiveness of a Brief Cognitive Intervention to Help Athletes Cope With Competition Loss | 1 | 0 | 1 | 0 | 1 | 0 | 0 | 1 | Participants + Not performance enhancement + Outcome |
| Asadi et al. (2019) Directing Attention Externally and Self-Controlled Practice Have Similar Effects on Motor Skill Performance | 1 | 0 | 1 | 1 | 1 | 1 | 0 | 1 | Participants |
| Asadi et al. (2018) The effect of task-relevant and task-irrelevant attentional cues and skill level on performance and knee kinematics of standing long jump | 1 | 1 | 1 | 1 | 1 | 0 | 0 | 1 | Outcome |
| Ashbrook et al. (2018) Effects of an Individualized Mental-Skills-Training Program on Golf Performance: A Single-Subject Research Design. | 1 | 1 | 1 | 1 | 0 | 1 | 0 | 1 | Design |
| Atienza & Balaguer (1998) Video modeling and imaging training on performance of tennis service of 9- to 12-year-old children | 1 | 0 | 1 | 1 | 1 | 1 | 1 | 0 | Participants |
| Azita et al. (2019) Effectiveness of Psychological Preparation Program on Sport Performance of Futsal Girl Players: Mediating Role of Personality | 1 | 0 | 1 | 1 | 1 | 1 | 0 | 1 | Participants |
| Badami et al. (2012) Feedback About More Accurate Versus Less Accurate Trials: Differential Effects on Self-Confidence and Activation | 1 | 0 | 1 | 1 | 1 | 1 | 0 | 1 | Participants |
| Baghurst et al. (2004) Evidence for a Relationship Between Attentional Styles and Effective Cognitive Strategies During Performance | 1 | 0 | 1 | 1 | 1 | 1 | 0 | 1 | Participants |
| Baltar & Filgueiras (2018) The Effects of Mindfulness Meditation on Attentional Control During Off-Season Among Football Players. | 1 | 1 | 1 | 1 | 1 | 0 | 0 | 1 | Outcome |
| Baltzell & Akhtar (2014) Mindfulness meditation training for sports (MMTS) intervention: Impact of MMTS with division I female athletes | 1 | 1 | 1 | 1 | 1 | 0 | 0 | 1 | Outcome |
| Bar-Eli & Blumenstein (2004) Performance enhancement in swimming: the effect of mental training with biofeedback | 1 |  | 0 |  |  |  |  |  | Intervention |
| Bar-Eli & Blumenstein (2004) The effect of extra-curricular mental training with biofeedback on short running performance of adolescent physical education pupils | 1 |  | 0 |  |  |  |  |  | Intervention |
| Bar-Eli et al. (1993) Effect of goal difficulty on performance of aerobic, anaerobic and power tasks in laboratory and field settings | 1 | 0 | 1 | 1 | 1 | 1 | 0 | 1 | Participants |
| Bar-Eli et al. (1997) Effect of goal difficulty, goal specificity and duration of practice time intervals on muscular endurance performance | 1 | 0 | 1 | 1 | 1 | 0 | 0 | 1 | Participants + Outcome |
| Bar-Eli et al. (2002) The effect of mental training with biofeedback on the performance of young swimmers | 1 |  | 0 |  |  |  |  |  | Intervention |
| Barker & Jones (2008) The effects of hypnosis on self-efficacy, affect, and soccer performance: A case study | 1 | 1 | 1 | 1 | 0 | 0 | 1 | 1 | Design |
| Barker et al. (2010) Assessing the immediate and maintained effects of hypnosis on self-efficacy and soccer wall-volley performance | 1 | 0 | 1 | 1 | 1 | 1 | 1 | 1 | Participants |
| Barling & Bresgi (1982) Cognitive Factors in Athletic (Swimming) Performance: A Re-Examination | 1 | 0 | 1 | 1 | 1 | 1 | 0 | 0 | Participants |
| Barlow & Bank (2004) Using emotional intelligence in coaching high-performance athletes: A randomised controlled trial | 1 | 1 | 1 | 1 | 1 | 0 | 0 | 1 | Outcome |
| Bartholomew (2003) Psychological states following a maximal exercise test: The impact of manipulated performance feedback in competitive athletes | 1 | 0 | 1 | 0 | 1 | 0 | 0 | 1 | Participants + Not performance enhancement + Outcome |
| Barwood et al. (2008) Psychological skills training improves exercise performance in the heat | 1 | 0 | 1 | 1 | 1 | 1 | 0 | 1 | Participants |
| Barwood et al. (2015) Improvement of 10-km time-trial cycling with motivational self-talk compared with neutral self-talk | 1 | 0 | 1 | 1 | 1 | 1 | 0 | 1 | Participants |
| Basset et al. (2022) Type of self-talk matters: Its effects on perceived exertion, cardiorespiratory, and cortisol responses during an iso-metabolic endurance exercise | 1 | 0 | 1 | 1 | 1 | 0 | 0 | 1 | Participants + Outcome |
| Battaglia et al. (2014) Use of video observation and motor imagery on jumping performance in national rhythmic gymnastics athletes | 1 | 0 | 1 | 1 | 1 | 1 | 0 | 1 | Participants |
| Baudry et al. (2006) The effect of combined self- and expert-modelling on the performance of the double leg circle on the pommel horse | 1 |  | 0 |  |  |  |  |  | Intervention |
| Beattie et al. (2016) The role of performance feedback on the self-efficacy-performance relationship | 1 |  | exp1: 0; exp2: 0; exp3: 0 |  |  |  |  |  | exp1: Intervention; exp2: Intervention; exp3: Intervention |
| Beauchamp et al. (2012) An integrated biofeedback and psychological skills training program for Canada's Olympic Short-Track Speedskating Team | 1 |  | 0 |  |  |  |  |  | Intervention |
| Becker & Wu (2015) Integrating biomechanical and motor control principles in elite high jumpers: A transdisciplinary approach to enhancing sport performance | 1 |  | 0 |  |  |  |  |  | Intervention |
| Beilock & Carr (2001) On the fragility of skilled performance: What governs choking under pressure? | 1 | exp3: 0; exp4: 0 | exp1: 0; exp2: 0; exp3: 1; exp4: 1 | exp3: 1; exp4: 1 | exp3: 1; exp4: 1 | exp3: 1; exp4: 1 | exp3: 0; exp4: 0 | exp3: 1; exp4: 0 | exp1: Intervention; exp2: Intervention; exp3: Participants exp4: Participants |
| Beilock & Gonso (2008) Putting in the mind versus putting on the green: expertise, performance time, and the linking of imagery and action | 1 | 0 | 1 | 1 | 1 | 1 | 0 | 1 | Participants |
| Beilock et al. (2001) "Don't miss!" The debilitating effects of suppressive imagery on golf putting performance | 1 | 0 | 1 | 1 | 1 | 1 | 0 | 1 | Participants |
| Beilock et al. (2002) Expertise, attention, and memory in sensorimotor skill execution: Impact of novel task constraints on dual-task performance and episodic memory | 1 | exp1: 0 exp2: 0 | exp1: 1  exp2: 1 | exp1: 1  exp2: 1 | exp1: 1 exp2: 1 | exp1: 1 exp2: 1 | exp1: 0 exp2: 0 | exp1: 1 exp2: 1 | exp1: Participants  exp2: Participants |
| Beilock et al. (2002) When Paying Attention Becomes Counterproductive: Impact of Divided Versus Skill-Focused Attention on Novice and Experienced Performance of Sensorimotor Skills | 1 | exp1: 0  exp2: 0 | exp1: 1  exp2: 1 | exp1: 1  exp2: 1 | exp1: 1 exp2: 1 | exp1: 1 exp2: 1 | exp1: 0 exp2: 0 | exp1: 1 exp2: 1 | exp1: Participants  exp2: Participants |
| Bell & Hardy (2009) Effects of Attentional Focus on Skilled Performance in Golf | 1 |  | 0 |  |  |  |  |  | Intervention |
| Benz et al. (2016) Coaching Instructions and Cues for Enhancing Sprint Performance | 1 |  | 0 |  |  |  |  |  | Intervention |
| Bernier et al. (2009) A multistudy investigation examining the relationship between mindfulness and acceptance approaches and sport performance | 1 | exp2: 0 | exp1: 0; exp2: 1 | exp2: 1 | exp2: 1 | exp2: 0 | exp2: 0 | exp2: 1 | exp1: Intervention; exp2: Participants + Outcome |
| Bernier et al. (2009) Mindfulness and acceptance approaches in sport performance | 1 | exp2: 0 | exp1: 0; exp2: 1 | exp2: 1 | exp2: 1 | exp2: 0 | exp2: 0 | exp2: 1 | exp1: Intervention; exp2: Participants + Outcome |
| Beuchamp et al. (1996) Effects of cognitive-behavioral psychological skills training on the motivation, preparation, and putting performance of novice golfers | 1 | 0 | 1 | 1 | 1 | 1 | 0 | 1 | Participants |
| Bieleke et al. (2019) Served Well? A Pilot Field Study on the Effects of Conveying Self-control Strategies on Volleyball Service Performance | 1 |  | 0 |  |  |  |  |  | Intervention |
| Binsch et al. (2008) Ironic effects in a simulated penalty shooting task: Is the negative wording in the instruction essential? | 1 | 0 | 1 | 1 | 1 | 1 | 0 | 1 | Participants |
| Binsch et al. (2010) Ironic effects and final target fixation in a penalty shooting task. | 1 | 0 | 1 | 1 | 1 | 1 | 0 | 1 | Participants |
| Bjorkly (2007) Soccer players' perceptions of their coping strategies: A screening evaluation before and after a sport psychology service delivery | 1 | 1 | 1 | 1 | 1 | 0 | 0 | 1 | Outcome |
| Björkstrand & Jern (2013) Evaluation of an imagery intervention to improve penalty taking ability in soccer: A study of two junior girls teams | 1 | 0 | 1 | 1 | 1 | 1 | 0 | 1 | Participants |
| Blanchfield et al. (2014) Talking yourself out of exhaustion: the effects of self-talk on endurance performance | 1 | 0 | 1 | 1 | 1 | 1 | 0 | 1 | Participants |
| Blankert & Hamstra (2017) Imagining success: Multiple achievement goals and the effectiveness of imagery | 1 | 0 | 1 | 1 | 1 | 1 | 0 | 0 | Participants |
| Bliznevsky et al. (2016) Formation of active-effective attitude of 12-13 years' judo athletes to sports functioning in competition period | 1 |  | 0 |  |  |  |  |  | Intervention |
| Blumenstein et al. (1995) The augmenting role of biofeedback: effects of autogenic, imagery and music training on physiological indices and athletic performance | 1 | 0 | 0 | 1 | 1 | 1 | 0 | 1 | Participants |
| Bonnar et al. (2022) Evaluation of a Brief Sleep Intervention Designed to Improve the Sleep, Mood, and Cognitive Performance of Esports Athletes | 1 | 1 | 1 | 1 | 1 | 0 | 0 | 1 | Outcome |
| Borjesson et al. (2018) Flotation REST as a Stress Reduction Method: The Effects on Anxiety, Muscle Tension, and Performance. | 1 |  | 0 |  |  |  |  |  | Intervention |
| Boroujeni et al. (2014) The effects of instructional and motivational self-talk on basketball passing and shooting performance in novice students | 1 | 0 | 1 | 1 | 1 | 1 | 0 | 0 | Participants |
| Bortoli et al. (2012) Striving for excellence: A multi-action plan intervention model for Shooters | 1 | 1 | 1 | 1 | 0 | 1 | 0 | 1 | Design |
| Boyce (1990) The Effect of Instructor-Set Goals Upon Skill Acquisition and Retention of a Selected Shooting Task | 1 | 0 | 1 | 1 | 1 | 1 | 0 | 0 | Participants |
| Boyce (1992) Effects of assigned versus participant-set goals on skill acquisition and retention of a selected shooting task | 1 | 0 | 1 | 1 | 1 | 1 | 0 | 0 | Participants |
| Boyce (1992) The effects of goal proximity on skill acquisition and retention of a shooting task in a field-based setting | 1 | 0 | 1 | 1 | 1 | 1 | 0 | 1 | Participants |
| Boyce (1994) The effects of goal setting on performance and spontaneous goal-setting behavior of experienced pistol shooters | 1 | 0 | 1 | 1 | 1 | 1 | 0 | 1 | Participants |
| Boyce & Bingham (1997) The effects of self-efficacy and goal setting on bowling performance | 1 | 0 | 1 | 1 | 1 | 1 | 0 | 1 | Participants |
| Boyce et al. (2001) The effects of three types of goal setting conditions on tennis performance: A field-based study | 1 | 0 | 1 | 1 | 1 | 1 | 0 | 1 | Participants |
| Bram (1995) Effects of batting performance feedback on motivational factors and batting performance in youth baseball | 1 |  | 0 |  |  |  |  |  | Intervention |
| Brewer et al. (2019) A mental warm-up for athletes | 1 | exp1: 0  exp2: 1 exp3: 0 | exp1: 1; exp2: 1; exp3: 1 | exp1: 1; exp2: 1; exp3: 1 | exp1: 1; exp2: 1; exp3: 1 | exp1: 0; exp2: 0; exp3: 0 | exp1: 0; exp2: 0; exp3: 0 | exp1: 1; exp2: 1; exp3: 1 | exp1: Participants + Outcome  exp2: Outcome exp3: Participants + Outcome |
| Brewer & Shillinglaw (1992) Evaluation of a psychological skills training workshop for male intercollegiate lacrosse players | 1 | 1 | 1 | 1 | 1 | 0 | 0 | 1 | Outcome |
| Briegel-Jones et al. (2013) A Preliminary Investigation into the Effect of Yoga Practice on Mindfulness and Flow in Elite Youth Swimmers | 1 | 0 | 1 | 1 | 1 | 0 | 0 | 1 | Participants + Outcome |
| Brouziyne & Molinaro (2005) Mental imagery combined with physical practice of approach shots for golf beginners | 1 | 0 | 1 | 1 | 1 | 1 | 0 | 0 | Participants |
| Bruton et al. (2019) Who said 'there is no ‘I’ in team'? The effects of observational learning content level on efficacy beliefs in groups | 1 |  | exp1: 0; exp2: 0; exp3: 0 |  |  |  |  |  | exp1: Intervention exp2: Intervention exp3: Intervention |
| Buck et al. (2016) The Effects of Mental Imagery with Video-Modeling on Self-Efficacy and Maximal Front Squat Ability | 1 | 0 | 1 | 1 | 1 | 1 | 0 | 1 | Participants |
| Bueno et al. (2008) Emotional and motivational mechanisms mediating the influence of goal setting on endurance athletes' performance | 1 | 0 | 1 | 1 | 1 | 1 | 0 | 1 | Participants |
| Burgmer & Englich (2013) Bullseye!: How power improves motor performance | 1 | exp1: 0; exp2: 0 | exp1: 1; exp2: 1 | exp1: 1; exp2: 1 | exp1: 1; exp2: 1 | exp1: 1; exp2: 1 | exp1: 0; exp2: 0 | exp1: 1; exp2: 1 | exp1: Participants exp2: Participants |
| Burhans et al. (1988) Mental imagery training: effects on running speed performance. / Entrainement mental: effets sur la performance de vitesse en course | 1 | 0 | 1 | 1 | 1 | 1 | 0 | 1 | Participants |
| Burton (2010) The roadmap: Examining the Impact of a Systematic Goal-Setting Program for Collegiate Womens Tennis Players | 1 | 1 | 1 | 1 | 1 | 0 | 0 | 1 | Outcome |
| Busccombe et al. (2014) Neurophysiological, psychological, sport and health dimensions of three meditation techniques | 1 | 0 | 1 | 1 | 1 | 0 | 0 | 1 | Participants + Outcome |
| Caird et al. (1999) Biofeedback and relaxation techniques improves running economy in sub-elite long distance runners | 1 |  | 0 |  |  |  |  |  | Intervention |
| Callow et al. (2006) Effects of Dynamic and Static Imagery on Vividness of Imagery, Skiing Performance, and Confidence | 1 | 0 | 1 | 1 | 1 | 1 | 0 | 1 | Participants |
| Callow et al. (2013) Performance improvements from imagery: evidence that internal visual imagery is superior to external visual imagery for slalom performance | exp1: 1; exp2: 1; exp3: 1 | exp1: 0; exp2: 0; exp3: 0 | exp1: 1; exp2: 1; exp3: 1 | exp1: 1; exp2: 1; exp3: 1 | exp1: 1; exp2: 1; exp3: 1 | exp1: 1; exp2: 1; exp3: 1 | exp1: 0; exp2: 0; exp3: 1 | exp1: 1; exp2: 1; exp3: 1 | exp1: Participants; exp2: Participants; exp3: Participants |
| Callow et al. (2017) Kinesthetic imagery provides additive benefits to internal visual imagery on slalom task performance | 1 | 0 | 1 | 1 | 1 | 1 | 0 | 1 | Participants |
| Campo et al. (2019) Emotional Intelligence (EI) Training Adapted to the International Preparation Constraints in Rugby: Influence of EI Trainer Status on EI Training Effectiveness | 1 | 0 | 1 | 1 | 1 | 0 | 0 | 1 | Participants + Outcome |
| Canal-Bruland et al. (2016) Can visual illusions be used to facilitate sport skill learning? | 1 |  | 0 |  |  |  |  |  | Intervention |
| Carraca et al. (2019) A pilot study of a mindfulness-based program (MBSOCCERP): The potential role of mindfulness, self-compassion and psychological flexibility on flow and elite performance in soccer athletes | 1 | 1 | 1 | 1 | 1 | 0 | 0 | 1 | Outcome |
| Carraca et al. (2019) Mindfull Compassion Training On Elite Soccer: Effects, Roles and Associations On Flow, Psychological Distress and Thought Supression | 1 | 1 | 1 | 1 | 1 | 0 | 0 | 1 | Outcome |
| Carraça et al. (2018) The Mindfulness- Based Soccer Program (MBSoccerP): Effects on Elite Athletes. Cuadernos de Psicología del Deporte | 1 | 1 | 1 | 1 | 1 | 0 | 0 | 1 | Outcome |
| Carstairs (1991) Psychology of athletic performance. 1970 | 1 |  | 0 |  |  |  |  |  | Intervention |
| Carter & Kelly (1997) Using traditional and paradoxical imagery interventions with reactant intramural athletes | 1 | 0 | 1 | 1 | 1 | 1 | 0 | 1 | Participants |
| Caserta & Singer (2007) The effectiveness of Situational awareness learning in response to video tennis match situations | 1 | 0 | 1 | 1 | 1 | 0 | 0 | 1 | Participants, Outcome |
| Caserta et al. (2007) Old dogs, new tricks: Training the perceptual skills of senior tennis players | 1 | 0 | 1 | 1 | 1 | 1 | 0 | 1 | Participants |
| Caudill & Weinberg (1983) Effects of varying the length of the psych-up interval on motor performance | 1 | 0 | 1 | 1 | 1 | 1 | 1 | 1 | Participants |
| Chang et al. (2014) Self-talk and softball performance: The role of self-talk nature, motor task characteristics, and self-efficacy in novice softball players | 1 | 0 | 1 | 1 | 1 | 1 | 0 | 1 | Participants |
| Chen et al. (2019) Mindfulness training enhances flow state and mental health among baseball players in Taiwan. | 1 | 0 | 1 | 1 | 1 | 1 | 0 | 1 | Participants |
| Chen et al. (2021) The Effects of Attentional Focus and Skill Level on the Performance of Golf Puttingment | 1 | 0 | 1 | 1 | 1 | 1 | 0 | 1 | Participants |
| Chen & Meggs (2021) The effects of Mindful Sport Performance Enhancement (MSPE) training on mindfulness, and flow in national competitive swimmers | 1 | 0 | 1 | 1 | 1 | 0 | 0 | 1 | Participants + Outcome |
| Cheng et al. (2015) Sensorimotor Rhythm Neurofeedback Enhances Golf Putting Performance | 1 |  | 0 |  |  |  |  |  | Intervention |
| Chow et al. (2014) Effects of external and internal attention focus training on foot-strike patterns in running | 1 |  | 0 |  |  |  |  |  | Intervention |
| Chow et al. (2021) Implementation and evaluation of a standardised performance profile intervention with collegiate athletes | 1 | 0 | 1 | 1 | 1 | 0 | 0 | 1 | Participants + Outcome |
| Claver et al. (2017) The Cognitive and Motivation Intervention Program in Youth Female Volleyball Players. | 1 | 0 | 1 | 1 | 1 | 0 | 1 | 1 | Participants |
| Cleary et al. (2006) Training physical education students to self-regulate during basketball free throw practice | 1 | 0 | 1 | 1 | 1 | 1 | 0 | 1 | Participants |
| Clingman & Hilliard (1990) Race walkers quicken their pace by tuning in, not stepping out | 1 | 0 | 1 | 1 | 1 | 1 | 0 | 0 | Particpants |
| Coelho et al. (2007) Imagery intervention in open and closed tennis motor skill performance | 1 | 0 | 1 | 1 | 1 | 1 | 0 | 0 | Participants |
| Cohn et al. (1990) Effects of a Cognitive-Behavioral Intervention on the Preshot Routine and Performance in Golf | 1 | 1 | 1 | 1 | 0 | 1 | 0 | 1 | Design |
| Cook & Crewther (2012) The effects of different pre-game motivational interventions on athlete free hormonal state and subsequent performance in professional rugby union matches | 1 | 0 | 1 | 1 | 1 | 1 | 1 | 1 | Participants |
| Corrêa et al. (2006) Goal setting in acquisition of a volleyball skill late in motor learning | 1 | 0 | 1 | 1 | 1 | 1 | 0 | 0 | Participants |
| Cote et al. (2019) A Qualitative Exploration of Division I Tennis Players Completing the Mindfulness Meditation Training for Sport 2.0 Program | 1 | 1 | 1 | 1 | 1 | 0 | 0 | 1 | Outcome |
| Couture et al. (1998) Can performance in a distance swim be improved by increasing a preferred cognitive thinking strategy? | 1 |  | 0 |  |  |  |  |  | Intervention |
| Crewther & Cook (2012) Effects of different post-match recovery interventions on subsequent athlete hormonal state and game performance | 1 | 0 | 1 | 1 | 1 | 0 | 1 | 1 | Participants |
| Crivelli (2019) Neurocognitive Enhancement Effects of Combined Mindfulness-Neurofeedback Training in Sport | 1 | 0 | 1 | 1 | 1 | 0 | 0 | 1 | Participants + Outcome |
| Crivoi do Carmo et al. (2022) Effects of different goal orientations and virtual opponents performance level on pacing strategy and performance in cycling time trials | 1 | 0 | 1 | 1 | 1 | 1 | 0 | 1 | Participants |
| Crocker (1989) A follow-up of cognitive-affective stress management training | 1 | 0 | 1 | 1 | 1 | 0 | 1 | 1 | Participants |
| Crocker (1989) Evaluating stress management training under competition conditions | 1 | 0 | 1 | 1 | 1 | 0 | 1 | 1 | Participants |
| Crocker et al. (1988) Cognitive-Affective Stress Management Training with high performance youth volleyball players: Effects on affect, cognition, and performance | 1 | 0 | 1 | 1 | 1 | 0 | 1 | 1 | Participants |
| Crombie et al. (2011) Increasing Emotional Intelligence in Cricketers: An Intervention Study | 1 | 1 | 1 | 1 | 1 | 0 | 0 | 1 | Outcome |
| Cumming (2002) Athletes' Use of Imagery in the Off-Season | 1 |  | 0 |  |  |  |  |  | Intervention |
| Cumming et al. (2006) Examining the Direction of Imagery and Self-Talk on Dart-Throwing Performance and Self Efficacy | 1 | 0 | 1 | 1 | 1 | 1 | 0 | 1 | Participants |
| Curry & Maniar (2004) Academic Course for Enhancing Student-Athlete Performance in Sport | 1 |  | 0 |  |  |  |  |  | Intervention |
| Cusack et al. (2015) Direct and Conceptual Replications of Burgmer & Englich (2012): Power May Have Little to No Effect on Motor Performance | 1 | exp1: 0; exp2: 0 | exp1: 1; exp2: 1 | exp1: 1; exp2: 1 | exp1: 1; exp2: 1 | exp1: 1; exp2: 1 | exp1: 0; exp2: 0 | exp1: 0; exp2: 0 | exp1: Participants; exp2: Participants; exp3 + exp4 + exp5: Not considered sport related enough in this review |
| Cutton & Landin (1999) The comparative effects of two cognitive learning strategies on learning the tennis forehand | 1 |  | 0 |  |  |  |  |  | Intervention |
| Cutton & Landin (2007) The effects of self-talk and augmented feedback on learning the tennis forehand | 1 | 0 | 1 | 1 | 1 | 1 | 0 | 1 | Participants |
| Czech et al. (2004) An Examination of the Maintenance of Preshot Routines in Basketball Free Throw Shooting | 1 |  | 0 |  |  |  |  |  | Intervention |
| Dallmann et al. (2016) Evaluation of a stress prevention program for young high-performance athletes | 1 | 1 | 1 | 0 | 1 | 0 | 0 | 1 | Not performance enhancement + Outcome |
| Dania (2021) Chess training for improving executive functions and invasion game tactical behavior of college student athletes: a preliminary investigation | 1 |  | 0 |  |  |  |  |  | Intervention |
| Daniels & Landers (1981) Biofeedback and shooting performance: A test of disregulation and systems theory | 1 |  | 0 |  |  |  |  |  | Intervention |
| Dan-Iulian & Anisoara (2018) Dimensions of the Psychological Training Program Assessment in Professional Sports | 1 | 0 | 1 | 1 | 1 | 0 | 0 | 1 | Participants + Outcome |
| Davidson & Edwards (2014) Evaluation of a mental skills training programme for high school rugby players | 1 | 0 | 1 | 1 | 1 | 0 | 1 | 1 | Participants |
| Davis (1991) Performance enhancement program for a college tennis player | 1 | 1 | 1 | 1 | 0 | 1 | 0 | 1 | Design |
| de la Pena (2009) The beneficial effects of anticipating anxiety-related symptoms: An investigation of paradoxical-success imagery in the laboratory | 1 | 0 | 1 | 1 | 1 | 1 | 0 | 1 | Participants |
| de la Pena et al. (2008) Implicit overcompensation: The influence of negative self-instructions on performance of a self-paced motor task | 1 |  | exp1: 0; exp2: 0 |  |  |  |  |  | exp1: Intervention exp2: Intervention |
| Dehghani et al (2018). Effectiveness of the mindfulness-acceptance-commitment-based approach on athletic performance and sports competition anxiety: a randomized clinical trial | 1 | 1 | 1 | 1 | 1 | 0 | 0 | 1 | Outcome |
| Dello Iacono et al. (2021) Ain't Just Imagination! Effects of Motor Imagery Training on Strength and Power Performance of Athletes during Detraining | 1 | 0 | 1 | 1 | 1 | 1 | 0 | 1 | Participants |
| De Muynck et al. (2017) The Effects of Feedback Valence and Style on Need Satisfaction, Self-Talk, and Perseverance Among Tennis Players: An Experimental Study. | 1 | 0 | 1 | 1 | 1 | 1 | 0 | 1 | Participants |
| DePaolo et al. (2019) Using a Behavioral Intervention to Improve Performance of a Women's College Lacrosse Team | 1 |  | 0 |  |  |  |  |  | Intervention |
| De Petrillo et al. (2009) Mindfulness for long-distance runners: An open trial using Mindful Sport Performance Enhancement (MSPE) | 1 | 0 | 1 | 1 | 1 | 1 | 0 | 1 | Participants |
| Deshayes et al. (2019) "Men are Better Than Women!" The Positive Effect of a Negative Stereotype Toward Women on a Self-Paced Cycling Exercise | 1 | 0 | 1 | 1 | 1 | 1 | 0 | 1 | Participants |
| De Sousa Fortes et al (2019) Effect of motor imagery training on tennis service performance in young tennis athletes | 1 | 0 | 1 | 1 | 1 | 1 | 0 | 0 | Participants |
| de Witt (1980) Cognitive and biofeedback training for stress reduction with university athletes | 1 |  | exp1: 0; exp2: 0 |  |  |  |  |  | exp1: Intervention exp2: Intervention |
| DeRenne & Morgan (2013) Multimodal modeling increases performance and biomechanical indicators in intercollegiate softball players: A longitudinal study | 1 | 0 | 1 | 1 | 1 | 1 | 0 | 0 | Participants |
| Deuskar et al. (2006) Effect of Yoga Nidra and Applied Relaxation Technique on Steadiness and Performance of Archers | 1 | 0 | 1 | 1 | 1 | 1 | 0 | 1 | Participants |
| Devonport et al. (2016) Introducing sport psychology interventions: Self-control implications | 1 | 0 | 1 | 1 | 1 | 0 | 1 | 1 | Participants |
| DeWolfe et al. (2020) Embrace the challenge: Acknowledging a challenge following negative self-talk improves performance | 1 | 0 | 1 | 1 | 1 | 1 | 0 | 1 | Participants |
| di Fronso et al. (2018) Focusing Attention on Muscle Exertion Increases EEG Coherence in an Endurance Cycling Task. | 1 | 0 | 1 | 1 | 1 | 1 | 0 | 1 | Participants |
| Didymus & Fletcher (2017) Effects of a cognitive-behavioral intervention on field hockey players' appraisals of organizational stressors. | 1 | 1 | 1 | 1 | 0 | 0 | 1 | 1 | Design |
| Donachie & Hill (2022)  Helping soccer players help themselves: Effectiveness of a psychoeducational book in reducing perfectionism | 1 | 0 | 1 | 1 | 1 | 0 | 0 | 1 | Participants + Outcome |
| Donohue et al. (2006) Effects of brief yoga exercises and motivational preparatory interventions in distance runners: results of a controlled trial | 1 | 0 | 1 | 1 | 1 | 1 | 1 | 0 | Participants |
| Donohue et al. (2018) Controlled Evaluation of an Optimization Approach to Mental Health and Sport Performance. | 1 | 0 | 1 | 1 | 1 | 0 | 0 | 1 | Participants + Outcome |
| Doron et al. (2020) Integrated mindfulness-based intervention: Effects on mindfulness skills, cognitive interference and performance satisfaction of young elite badminton players | 1 | 0 | 1 | 1 | 1 | 0 | 1 | 1 | Participants |
| Ducrocq et al. (2016) Training Attentional Control Improves Cognitive and Motor Task Performance | 1 | exp2: 0; exp3: 0 | exp2: 1 exp3: 1 | exp2: 1; exp3: 1 | exp2: 1; exp3: 1 | exp2: 0 exp3: 1 | exp2: 0; exp3: 0 | exp2: 1; exp3: 1 | exp1: Not considered sport related enough in this review; exp2: Participants + Outcome; exp3: Participants |
| Ducrocq et al. (2017) Adaptive Working Memory Training Reduces the Negative Impact of Anxiety on Competitive Motor Performance. | 1 |  | 0 |  |  |  |  |  | Intervention |
| Dutra et al. (2017) Difficult group goal improves serve reception of experienced volleyball players. | 1 | 0 | 1 | 1 | 1 | 1 | 0 | 1 | Participants |
| Edens (1994) Enhance performance through imagery | 1 |  | 0 |  |  |  |  |  | Intervention |
| Edwards & Steyn (2008) Sport Psychological Skills Training and Pychological Well-Being | 1 | exp1: 0; exp2: 0; exp3: 1 | exp1: 1; exp2: 1; exp3: 1 | exp1: 1; exp2: 1; exp3: 1 | exp1: 1; exp2: 1; exp3: 0 | exp1: 1; exp2: 0; exp3: 0 | exp1: 0; exp2: 0; exp3: 0 | exp1: 1; exp2: 1; exp3: 0 | exp1: Participants; exp2: Participants + Outcome; exp3: Design + Outcome |
| Edwards et al. (2008) Self-talk influences vertical jump performance and kinematics in male rugby union players | 1 | 1 | 1 | 1 | 1 | 0 | 0 | 1 | Outcome |
| Elko & Ostrow (1991) Effects of a rational-emotive education program on heightened anxiety levels of female collegiate gymnasts | 1 | 1 | 1 | 1 | 0 | 1 | 0 | 1 | Design |
| Englert & Bertrams (2015) Autonomy as a protective factor against the detrimental effects of ego depletion on tennis serve accuracy under pressure | 1 | 0 | 1 | 1 | 1 | 1 | 0 | 1 | Participants |
| Epstein (1980) The relationship of mental imagery and mental rehearsal to performance of a motor task | 1 | 0 | 1 | 1 | 1 | 1 | 0 | 1 | Participants |
| EunKyung & JungTaek (2016) The Effects of Performance Routine on Competitive State Anxiety, Psychological Skills, and Perceived Performance of Taekwondo Poomsae Players | 1 | 1 | 1 | 1 | 0 | 0 | 1 | 1 | Design |
| Evans et al. (2013) Using Personal-Disclosure Mutual-Sharing to Enhance Group Functioning in a Professional Soccer Academy | 1 | 0 | 1 | 1 | 1 | 1 | 0 | 1 | Participants |
| Eyal et al. (1995) Manipulated outcome expectations and competitive performance in motor-tasks with gradually increasing difficulty | 1 | 0 | 1 | 1 | 1 | 1 | 0 | 1 | Participants |
| Fairall & Rodgers (1997) The effects of goal-setting method on goal attributes in athletes: A field experiment | 1 | 1 | 1 | 1 | 1 | 0 | 0 | 1 | Outcome |
| Fairweather & Potgieter (1993) The effect of pre-shot strategies on golf putting | 1 | 0 | 1 | 1 | 1 | 1 | 0 | 0 | Participants |
| Fallon et al. (2014) A Brief Mindfulness and Yoga Intervention with an Entire NCAA Division I Athletic Team: An Initial Investigation | 1 | 1 | 1 | 1 | 1 | 0 | 0 | 1 | Outcome |
| Faulkner et al. (2010) Effect of accurate and inaccurate distance feedback on performance markers and pacing strategies during running | 1 | 1 | 1 | 1 | 1 | 0 | 0 | 1 | Outcome |
| Fazel et al. (2018) The effects of different types of imagery delivery on basketball free-throw shooting performance and self-efficacy. | 1 | 0 | 1 | 1 | 1 | 1 | 0 | 1 | Participants |
| Fekih et al. (2020) Effects of Motor Mental Imagery Training on Tennis Service Performance during the Ramadan Fasting: a Randomized, Controlled Trial | 1 | 0 | 1 | 1 | 1 | 1 | 0 | 0 | Participants |
| Fekih et al. (2020) The Impact of a Motor Imagery-Based Training Program on Agility, Speed, and Reaction Time in a Sample of Young Tennis Athletes during Ramadan Fasting: Insights and Implications from a Randomized, Controlled Experimental Trial | 1 | 0 | 1 | 1 | 1 | 1 | 0 | 1 | Participants |
| Fenker & Lambiotte (1987) A performance enhancement program for a college football team: One incredible season | 1 | 1 | 1 | 1 | 1 | 0 | 0 | 1 | Outcome |
| Fensterheim (1980) Behavioral method for improving sport performance | 1 |  | 0 |  |  |  |  |  | Intervention |
| Fereira-Vorkapic et al. (2017) The impact of yoga-based interventions on jiu-jitsu wrestlers' psychological profile: a pilot study. | 1 | 0 | 1 | 1 | 1 | 0 | 0 | 1 | Participants + Outcome |
| Filby et al. (1999) The effect of multiple-goal strategies on performance outcomes in training and competition | 1 | 0 | 1 | 1 | 1 | 1 | 0 | 1 | Participants |
| Fisher & Etnier (2014) Examining the time course of attention during golf putts of two different lengths in experienced golfers | 1 |  | 0 |  |  |  |  |  | Intervention |
| Fisher et al. (1986) Subliminal oedipal stimuli and competitive performance: An investigation of between-groups effects and mediating subject variables | 1 | 0 | 1 | 1 | 1 | 1 | 0 | 1 | Participants |
| Fitzimmons et al. (1991) Does self-efficacy predict performance in experienced weightlifters? | 1 | 0 | 1 | 1 | 1 | 1 | 0 | 1 | Participants |
| Fogaca (2021) Combining mental health and performance interventions: Coping and social support for student-athletes | 1 | 1 | 1 | 1 | 1 | 0 | 0 | 1 | Outcome |
| Fontani & Migliorini (2012) A cognitive approach to physical exercise and sport | 1 |  | 0 |  |  |  |  |  | Intervention |
| Ford et al. (2005) Online Attentional-Focus Manipulations in a Soccer-Dribbling Task: Implications for the Proceduralization of Motor Skills | 1 | 0 | 1 | 1 | 1 | 1 | 0 | 1 | Participants |
| Ford et al. (2007) Examining action effects in the execution of a skilled soccer kick by using erroneous feedback | 1 | 0 | 1 | 1 | 1 | 1 | 0 | 0 | Participants |
| Forlenza et al. (2013) Imagery Speed and Self-Efficacy: How Fast (or Slow) To Go? | 1 | 0 | 1 | 1 | 1 | 1 | 0 | 1 | Participants |
| Forsblom et al. (2019) Perceived goal setting practices across a competitive season | 1 |  | 0 |  |  |  |  |  | Intervention |
| Fortes et al. (2020) Effect of an eight-week imagery training programme on passing decision-making of young volleyball players | 1 | 0 | 1 | 1 | 1 | 1 | 0 | 1 | Participants |
| Foster et al. (2006) The Effect of Removing Superstitious Behavior and Introducing a Pre-Performance Routine on Basketball Free-Throw Performance | 1 | 0 | 1 | 1 | 1 | 1 | 0 | 0 | Participants |
| Fournier et al. (2005) Effects of a Season-Long PST Program on Gymnastic Performance and on Psychological Skill Development | 1 | 0 | 1 | 1 | 1 | 1 | 0 | 1 | Participants |
| Fransen et al. (2017) The power of competence support: The impact of coaches and athlete leaders on intrinsic motivation and performance. | 1 | 0 | 1 | 1 | 1 | 1 | 1 | 1 | Participants |
| Fransen et al. (2016) We will be champions: Leaders' confidence in 'us' inspires team members' team confidence and performance | 1 | 0 | 1 | 1 | 1 | 1 | 1 | 1 | Participants |
| Fu et al. (2021) The effects of support (in)adequacy on self-confidence and performance: Two experimental studies | 1 |  | exp1: 0; exp2: 0 |  |  |  |  |  | exp1: Intervention exp2: Intervention |
| Gagnon-Dolbec et al. (2019) Feedback, sport-confidence and performance of lacrosse skills | 1 | 0 | 1 | 1 | 1 | 1 | 0 | 1 | Participants |
| Gal-Or et al. (1986) Cognitive behavioral strategies and anxiety in elite orienteers | 1 |  | 0 |  |  |  |  |  | Intervention |
| Garcia et al. (2015) Dynamic Stability of Task-Related Thoughts in Trained Runner | 1 | 0 | 1 | 1 | 1 | 1 | 0 | 1 | Participants |
| Garza & Feltz (1998) Effects of selected mental practice on performance, self-efficacy, and competition confidence of figure skaters | 1 | 0 | 1 | 1 | 1 | 0 | 1 | 1 | Participants |
| Getz & Rainey (2001) Flexible short-term goals and basketball shooting performance | 1 | exp1: 0; exp2: 0 | exp1: 1; exp2: 1 | exp1: 1; exp2: 1 | exp1: 1, exp2: 1 | exp1: 1; exp2: 1 | exp1: 0; exp2: 0 | exp1: 0; exp2: 0 | exp1:Participants; exp2: Participants |
| Giannini et al. (1988) The Effects of Mastery, Competitive, and Cooperative Goals on the Performance of Simple and Complex Basketball Skills | 1 | 0 | 1 | 1 | 1 | 1 | 0 | 0 | Participants |
| Goisbault et al. (2022) An integrated mindfulness and acceptance-based program for young elite female basketball players: Exploratory study of how it works and for whom it works best | 1 | 0 | 1 | 1 | 1 | 0 | 1 | 1 | Participants |
| Goudas & Theodorakis (1998) Psychological skills in basketball: Preliminary study for development of a Greek form of the | 1 |  | 0 |  |  |  |  |  | Intervention |
| Goudas et al. (2006) The effects of self-talk on throwing- and jumping-events performance | 1 | exp1: 0; exp2: 0 | exp1: 1; exp2: 1 | exp1: 1; exp2: 1 | exp1: 1; exp2: 1 | exp1: 1; exp2: 1 | exp1: 0; exp2: 0 | exp1: 1; exp2: 1 | exp1: Participants exp2: Participants |
| Gould & Weiss (1981) The effects of model similarity and model talk on self-efficacy and muscular endurance | 1 | 0 | 1 | 1 | 1 | 1 | 0 | 1 | Participants |
| Gravel et al. (1980) Effectiveness of a Cognitive Behavioral Treatment Package for Cross-Country Ski Racers | 1 | 1 | 1 | 1 | 1 | 0 | 0 | 1 | Outcome |
| Gray (2018) Comparing Cueing and Constraints Interventions for Increasing Launch Angle in Baseball Batting | 1 |  | 0 |  |  |  |  |  | Intevention |
| Gray & Allsop (2013) Interactions Between Performance Pressure, Performance Streaks, and Attentional focus | 1 | exp2: 1 | exp1: 0  exp2: 1 | exp2: 1 | exp2: 1 | exp2: 0 | exp2: 0 | exp2: 1 | exp1: Intervention exp2: Outcome |
| Gregg et al. (2004) A Mental Skills Package for Special Olympics Athletes: A Preliminary Study | 1 | 0 | 1 | 1 | 0 | 1 | 0 | 1 | Participants + Design |
| Grobbelaar (2018) Effects of a psychological skills training programme for underserved rugby union players | 1 | 1 | 1 | 1 | 1 | 0 | 0 | 1 | Outcome |
| Grosu et al. (2015) Influence of Mental Training on Focused Attention and Manual Response in Alpine Skiing | 1 | 0 | 1 | 1 | 1 | 0 | 0 | 1 | Participants + Outcome |
| Grosu et al. (2015) Sensory submodalities testing in neurolinguistic programming, part of mental training | 1 |  | 0 |  |  |  |  |  | Intervention |
| Grouios (1992) The effect of mental practice on diving performance | 1 | 0 | 1 | 1 | 1 | 1 | 0 | 0 | Participants |
| Grouios et al. (1997) The effect of a simulated mental practice technique on free throw shooting accuracy of highly skilled basketball players | 1 | 0 | 1 | 1 | 1 | 1 | 0 | 0 | Participants |
| Gunes & Yilmaz (2019) The Effect of Tactical Games Approach in Basketball Teaching on Cognitive, Affective and Psychomotor Achievement Levels of High School Students | 1 |  | 0 |  |  |  |  |  | Intervention |
| Guillot et al. (2010) Does motor imagery enhance stretching and flexibility? | 1 | 1 | 1 | 1 | 1 | 0 | 0 | 1 | Outcome |
| Guillot et al. (2012) Motor imagery and ‘placebo-racket effects’ in tennis serve performance | 1 | 0 | 1 | 1 | 1 | 1 | 1 | 1 | Participants |
| Guillot et al. (2013) Motor imagery and tennis serve performance: the external focus efficacy | 1 | 0 | 1 | 1 | 1 | 1 | 0 | 1 | Participants |
| Guillot et al. (2015) Implementation of Motor Imagery during Specific Aerobic Training Session in Young Tennis Players | 1 | 0 | 1 | 1 | 1 | 1 | 0 | 1 | Participants |
| Haase et al. (2015) A pilot study investigating changes in neural processing after mindfulness training in elite athletes | 1 | 1 | 1 | 1 | 1 | 0 | 0 | 1 | Outcome |
| Hacker (2000) Women's World Cup: Performance enhancement through mental skills training | 1 |  | 0 |  |  |  |  |  | Intervetion |
| Hale & Whitehouse (1998) The effects of imagery-manipulated appraisal on intensity and direction of competitive anxiety | 1 | 0 | 1 | 1 | 1 | 0 | 0 | 1 | Participants + Outcome |
| Hall & Hardy (1991) Ready, aim, fire...relaxation strategies for enhancing pistol marksmanship | 1 | 0 | 1 | 1 | 1 | 1 | 0 | 0 | Participants |
| Hall & Rodgers (1989) Enhancing Coaching Effectiveness in Figure Skating Through a Mental Skills Training Program | 1 | 0 | 1 | 1 | 1 | 0 | 0 | 1 | Participants |
| Hall et al. (1998) Imagery use by athletes: Development of the Sport Imagery Questionnaire | 1 |  | 0 |  |  |  |  |  | Intervention |
| Halperin et al. (2016) The Effects of Attentional Focusing Instructions on Force Production During the Isometric Midthigh Pull | 1 |  | 0 |  |  |  |  |  | Intervention |
| Halperin et al. (2017) The effects of attentional focus instructions on punching velocity and impact forces among trained combat athletes | 1 |  | 0 |  |  |  |  |  | Intervention |
| Halvari (1996) Effects of mental practice on performance are moderated by cognitive anxiety as measured by the | 1 | 0 | 1 | 1 | 1 | 1 | 0 | 1 | Participants |
| Hammoudi-Nassib et al. (2014) Time interval moderates the relationship between psyching-up and actual sprint performance | 1 | 0 | 1 | 1 | 1 | 1 | 0 | 1 | Participants |
| Hanrahan (1995) Psychological skills training for competitive wheelchair and amputee athletes | 1 | 1 | 1 | 1 | 1 | 0 | 0 | 1 | Outcome |
| Hanrahan et al. (1990) Psychological Skills Training for the Blind Athlete: A Pilot Program | 1 | 0 | 1 | 1 | 1 | 0 | 0 | 1 | Participants + Outcome |
| Hanrahan et al. (1995) Use of imagery while performing dance movement. | 1 | 0 | 1 | 1 | 1 | 1 | 0 | 0 | Participants |
| Hanshaw & Sukal (2016) Effect of self-talk and imagery on the response time of trained martial artists | 1 | 0 | 1 | 1 | 1 | 1 | 0 | 0 | Participants |
| Harada et al. (2016) Intervention study using a leaflet entitled ‘three benefits of “go to bed early! Get up early! And intake nutritionally rich breakfast!” a message for athlethes’ to improve the soccer performance of university soccer team | 1 |  | 0 |  |  |  |  |  | Intervention |
| Harari (1969) Level of aspiration and athletic performance | 1 | 0 | 1 | 1 | 1 | 1 | 0 | 0 | Participants |
| Hardy & Callow (1999) Efficacy of external and internal visual imagery perspectives for the enhancement of performance on tasks in which form is important | 1 | exp1: 0; exp2: 0; exp3: 0 | exp1: 1; exp2: 1; exp3: 1 | exp1: 1; exp2: 1; exp3: 1 | exp1: 1; exp2: 1; exp3: 1 | exp1: 1; exp2: 1; exp3: 1 | exp1: 0; exp2: 0; exp3: 1 | exp1: 1; exp2: 1; exp3: 1 | Exp1: Participants; exp2: Participants; Exp3: Participants |
| Hardy & Callow (1999) Efficacy of external and internal visual imagery perspectives for the enhancement of performance on tasks in which form is important | 1 | exp1: 0 exp2: 0 exp3: 0 | exp1: 1  exp2: 1  exp3: 1 | exp1: 1 exp2: 1 exp3: 1 | exp1: 1; exp2:1; exp3: 1 | exp1: 1 exp2: 1 exp3: 1 | exp1: 0 exp2: 0  exp3: 1 | exp1: 1 exp2: 1 exp3: 1 | exp1: Participants exp2: Participants exp3: Participants |
| Hardy & Callow (1999) Efficacy of External and Internal Visual Imagery Perspectives for the Enhancement of Performance on Tasks in Which Form Is Important | 1 | exp1: 0; exp2: 0; exp3: 0 | exp1: 1; exp2: 1; exp3: 1 | exp1: 1; exp2: 1; exp3: 1 | exp1: 1; exp2:1; exp3: 1 | exp1: 1; exp2: 1; exp3: 1 | exp1: 0; exp2: 0; exp3: 1 | exp1: 1; exp2: 1; exp3: 1 | exp1: Participants; exp2: Participants; exp3: Participants |
| Hardy et al. (2015) It's good but it's not right: Instructional self-talk and skilled performance | 1 | 0 | 1 | 1 | 1 | 1 | 0 | 1 | Participants |
| Harrison et al. (2021) The Effectiveness of Virtual Reality on Anxiety and Performance in Female Soccer Players | 1 |  | 0 |  |  |  |  |  | Intervention |
| Harvey et al. (2002) Relationship between self-talk and golf performance | 1 | 0 | 1 | 1 | 1 | 1 | 0 | 1 | Participants |
| Harvey et al. (2002) Relationship between self-talk and golf performance | 1 | 0 | 1 | 1 | 1 | 1 | 0 | 0 | Participants |
| Hase et al. (2019) The influence of self-talk on challenge and threat states and performance | 1 | 0 | 1 | 1 | 1 | 1 | 0 | 1 | Participants |
| Hasegawa et al. (2020) Practice motions performed during preperformance preparation drive the actual motion of golf putting | 1 |  | 0 |  |  |  |  |  | Intervention |
| Hatzigeorgiadis (2004) Self-Talk in the Swimming Pool: The Effects of Self-Talk on Thought Content and Performance on Water-Polo Tasks | 1 | exp1: 0; exp2: 0 | exp1: 1; exp2: 1 | exp1: 1; exp2: 1 | exp1: 1; exp2: 1 | exp1: 1; exp2: 1 | exp1: 0; exp2: 0 | exp1: 1; exp2: 1 | exp1: Participants exp2: Participants |
| Hatzigeorgiadis et al. (2007) The moderating effects of self-talk content on self-talk functions | 1 | 0 | 1 | 1 | 1 | 1 | 0 | 1 | Participants |
| Hatzigeorgiadis et al. (2008) Investigating the functions of self-talk: The effects of motivational self-talk on self-efficacy and performance in young tennis players | 1 | 0 | 1 | 1 | 1 | 1 | 0 | 1 | Participants |
| Hatzigeorgiadis et al. (2009) Mechanisms underlying the self-talk-performance relationship: The effects of motivational self-talk on self-confidence and anxiety | 1 | 0 | 1 | 1 | 1 | 1 | 0 | 1 | Participants |
| Hatzigeorgiadis et al. (2014) Self-talk and competitive sport performance | 1 | 0 | 1 | 1 | 1 | 1 | 0 | 1 | Participants |
| Hatzigeorgiadis et al. (2014) Self-talk and Competitive Sport Performance | 1 | 0 | 1 | 1 | 1 | 1 | 0 | 1 | Participants |
| Hazell et al. (2014) An exploration of pre-performance routines, self-efficacy, anxiety and performance in semo-professional soccer | 1 | 0 | 1 | 1 | 1 | 1 | 0 | 1 | Participants |
| Heydari et al. (2018) The effect of Psychological skills training (goal setting, positive self-talk and Imagery) on self-confidence of adolescent volleyball players. | 1 | 0 | 1 | 1 | 1 | 0 | 0 | 1 | Participants + Outcome |
| Hill & Borden (1995) The effect of attentional cueing scripts on competitive bowling performance | 1 | 0 | 1 | 1 | 1 | 1 | 0 | 1 | Participants |
| Hill et al. (2020) The influence of mindfulness training on running economy and perceived flow under different attentional focus conditions – an intervention study | 1 | 0 | 1 | 1 | 1 | 0 | 0 | 1 | Participants + Outcome |
| Hird et al. (1991) Physical Practice Is Superior to Mental Practice in Enhancing Cognitive and Motor Task Performance | 1 | 0 | 1 | 1 | 1 | 1 | 0 | 0 | Participants |
| Hoffman (1983) An analysis of the relationship between psychical impetus and physiology among competitive athletes | 1 | 0 | 1 | 1 | 1 | 1 | 0 | 1 | Participants |
| Hoffman (1983) Effects of psychological momentum on the physiology and cognition among American athletes | 1 | 0 | 1 | 1 | 1 | 1 | 0 | 1 | Participants |
| Hoja & Jansen (2019) Mindfulness-based intervention for tennis players: a quasi-experimental pilot study | 1 | 0 | 1 | 1 | 1 | 1 | 0 | 1 | Participants |
| Holguin-Ramirez et al. (2020) Effect of Mindfulness on the Stress-Recovery Balance in Professional Soccer Players during the Competitive Season | 1 | 0 | 1 | 1 | 1 | 0 | 0 | 1 | Participants + Outcome |
| Hong et al. (2020) An empirical test of the self-talk dissonance hypothesis: The effects of self-talk overtness and personality on performance | 1 |  | 0 |  |  |  |  |  | Intervention |
| Horcajo et al. (2019) The Effects of Overt Head Movements on Physical Performance After Positive Versus Negative Self-Talk | 1 | 0 | 1 | 1 | 1 | 1 | 0 | 0 | Participants |
| Horcajo et al. (2022) The effects of self-efficacy on physical and cognitive performance: An analysis of meta-certainty | 1 | exp2: 0 | exp1: 0; exp2: 1 | exp2: 1 | exp2: 1 | exp2: 1 | exp2: 0 | exp2: 1 | exp1: Intervention exp2: Participants exp3: Not considered sport related enough in this review |
| Hossner & Ehrlenspiel (2010) Time-referenced effects of an internal vs. external focus of attention on muscular activity and compensatory variability | 1 | exp2: 1 | exp2: 1 | exp2: 1 | exp2: 1 | exp2: 0 | exp2: 0 | exp2: 1 | exp1: Not considered sport related enough in this review  exp2: Outcome |
| Howard & Reardon (1986) Changes in the self concept and athletic performance of weight lifters through a cognitive-hypnotic approach: an empirical study | 1 | 0 | 1 | 1 | 1 | 1 | 0 | 1 | Participants |
| Howe (1991) Imagery and sport performance | 1 |  | 0 |  |  |  |  |  | Intervention |
| Huda et al. (2021) Diverse Predictive Influence of Mood Factors on VMBR and Biofeedback Interventions Enhancing Thirty-Meter Dash Performance | 1 | 0 | 1 | 1 | 1 | 0 | 0 | 1 | Participants + Outcome |
| Huda et al. (2021) Multidimensional Extrapolative Impacts of Mood Factors on VMBR AND Biofeedback Interventions Facilitating Agility Performance | 1 | 0 | 1 | 1 | 1 | 0 | 0 | 1 | Participants + Outcome |
| Hut et al. (2021) The effects of mindfulness training on mindfulness, anxiety, emotion dysregulation, and performance satisfaction among female student-athletes: The moderating role of age | 1 | 1 | 1 | 1 | 1 | 0 | 0 | 1 | Outcome |
| Hüttermann & Memmert (2018) The role of regulatory focus and expectation on creative decision making | 1 | 1 | 1 | 1 | 1 | 0 | 0 | 1 | Outcome |
| Iglesias & Iglesias (2011) Clinical hypnosis with a Little League baseball population: performance enhancement and resolving traumatic experiences | 1 | 0 | 1 | 1 | 0 | 0 | 0 | 0 | Participants + Design + Outcome |
| In de Braek et al. (2019) Working Memory Training in Professional Football Players: A Small-Scale Descriptive Feasibility Study-The Importance of Personality, Psychological Well-Being, and Motivational Factors | 1 |  | 0 |  |  |  |  |  | Intervention |
| Ipinmoroti (2005) Effectiveness of Goal Specificity and Goal Difficulty as Motivational Strategies for Accuracy Performance of College Physical and Health Education | 1 | 0 | 1 | 1 | 1 | 1 | 0 | 0 | Participants |
| Ismail & Ismail (2019) The Effects of Practice in Mind (PIM) Training on Performance Strategies used by Professional University Football Players | 1 | 1 | 1 | 1 | 1 | 0 | 0 | 1 | Outcome |
| Ismail (2016) Effectiveness of "PIM" Training on Putting Performance and Pre-Competitive Anxiety of the Golfers | 1 | 0 | 1 | 1 | 1 | 1 | 0 | 1 | Participants |
| Ivarsson et al. (2015) It Pays to Pay attention: A Mindfulness-Based Program for Injury Prevention with Soccer Players | 1 | 0 | 1 | 1 | 1 | 0 | 0 | 1 | Participants + Outcome |
| Jackson (1980) Mental Preparation Strategies, Cognitions, and Strength Performance | 1 | exp1: 0 exp2: 0 | exp1: 1  exp2: 1 | exp1: 1 exp2: 1 | exp1: 1 exp2: 1 | exp1: 1 exp2: 1 | exp1: 0 exp2: 0 | exp1: 1 exp2: 1 | exp1: Participants exp2: Participants |
| Jaenes et al. (2021) The Effectiveness of the Psychological Intervention in Amateur Male Marathon Runners | 1 | 0 | 1 | 1 | 1 | 0 | 0 | 1 | Participants + Outcome |
| Jalene & Wulf (2014) Brief hypnotic intervention increases throwing accuracy | 1 | 0 | 1 | 1 | 1 | 1 | 0 | 1 | Participants |
| Jeong-Keun et al. (2021) Psychological Skill Training to Improve Golfer's Performance | 1 |  | 0 |  |  |  |  |  | Intervention |
| John et al. (2012) The effect of music therapy and meditation on sports performance in professional shooters. | 1 | 0 | 1 | 1 | 1 | 1 | 0 | 0 | Participants |
| Johnson & Kramer (1961) Effects of stereotyped non-hypnotic, hypnotic, and posthypnotic suggestions upon strength, power, and endurance | 1 | 0 | 1 | 1 | 1 | 0 | 0 | 1 | Participants + Outcome |
| Johnston & McCabe (1993) Cognitive strategies for coping with stress in a simulated golfing task | 1 | 0 | 1 | 1 | 1 | 1 | 1 | 1 | Participants |
| Jones et al. (2016) Deception has no acute or residual effect on cycling time trial performance but negatively effects perceptual responses | 1 | 0 | 1 | 1 | 1 | 1 | 0 | 1 | Participants |
| Jones et al. (2016) Improvements in Cycling Time Trial Performance Are Not Sustained Following the Acute Provision of Challenging and Deceptive Feedback | 1 | 0 | 1 | 1 | 1 | 1 | 0 | 1 | Participants |
| Josefsson et al. (2019) Effects of Mindfulness-Acceptance-Commitment (MAC) on Sport-Specific Dispositional Mindfulness, Emotion Regulation, and Self-Rated Athletic Peroformance in a Multiple-Sport Population: an RCT Study | 1 | 0 | 1 | 1 | 1 | 0 | 1 | 1 | Participants |
| Kanniyan (2015) Competitive state anxiety: Impact of positive self-talk training on junior level football players | 1 | 0 | 1 | 1 | 1 | 0 | 0 | 1 | Participants + Outcome |
| Kassim & Isa (2017) The influence of self-talk technique for male hockey goalkeeper. | 1 |  | 0 |  |  |  |  |  | Intervention |
| Kaufman et al. (2009) Evaluation of Mindful Sport Performance Enhancement (MSPE): A new approach to promote flow in athletes | 1 | 0 | 1 | 1 | 1 | 1 | 1 | 1 | Participants |
| Kearns & Crossman (1992) Effects of a cognitive intervention package on the free-throw performance of varsity basketball players during practice and competition | 1 | 1 | 1 | 1 | 0 | 1 | 0 | 1 | Design |
| Kelsey (1961) Effects of Mental Practice and Physical Practice upon Muscular Endurance | 1 | 0 | 1 | 1 | 1 | 1 | 1 | 0 | Participants |
| Kendall et al. (1990) The effects of an imagery rehearsal, relaxation, and self-talk package on basketball game performance | 1 | 1 | 1 | 1 | 3 | 1 | 0 | 1 | Design |
| Kenitzer & Briddell (1991) Using mental imagery to enhance athletic performance | 1 |  | 0 |  |  |  |  |  | Intervention |
| Kerr & Leith (1993) Stress management and athletic performance | 1 | 0 | 1 | 1 | 1 | 1 | 0 | 1 | Participants |
| Kerr et al. (1997) Effects on archery performance of manipulating metamotivational state and felt arousal | 1 | 0 | 1 | 1 | 1 | 1 | 0 | 1 | Participants |
| Khan et al. (2017) Portable devices for delivering imagery and modeling interventions: effects on netball players' adherence, shooting performance, and self-efficacy | 1 | 0 | 1 | 1 | 1 | 1 | 0 | 1 | Participants |
| Khodaskar (2016) Effect Of Dynamic Muscle Relaxation Training On Competitive Anxiety Of Male Inter-Collegiate Basketball Players | 1 | 1 | 1 | 1 | 1 | 0 | 0 | 1 | Outcome |
| Kim & Tennant (1993) Effects of visualization and Danjeon breathing on target shooting with an air pistol | 1 | 0 | 1 | 1 | 1 | 1 | 0 | 0 | Participants |
| Kingston & Hardy (1997) Effects of different types of goals on processes that support performance | 1 | 0 | 1 | 1 | 1 | 1 | 0 | 1 | Participants |
| Kirchenbaum et al. (1982) Effects of differential self-monitoring and level of mastery on sports performance: Brain power bowling | 1 | 0 | 1 | 1 | 1 | 1 | 0 | 0 | Participants |
| Kirschenbaum et al. (1982) Effects of differential self-monitoring and level of mastery of sports performance: Brain power bowling | 1 | 0 | 1 | 1 | 1 | 1 | 0 | 0 | Participants |
| Kirschenbaum et al. (1998) Smart Golf: Preliminary evaluation of a simple, yet comprehensive, approach to improving and scoring the mental game | 1 | 0 | 1 | 1 | 1 | 1 | 0 | 1 | Participants |
| Klodecka-Roxalska (1989) Autoregulation and the ability to cope with stress in sport activity | 1 |  | 0 |  |  |  |  |  | Intervention |
| Klostermann et al. (2015) Perceptual Training in Beach Volleyball Defence: Different Effects of Gaze-Path Cueing on Gaze and Decision-Making | 1 |  | 0 |  |  |  |  |  | Intervention |
| Klämpfl et al. (2013) Reinvestment--the cause of the yips? | 1 | 0 | 1 | 1 | 1 | 1 | 0 | 1 | Participants |
| Koh et al. (2017) The use of reflection-card by elite youth basketball players, head coach and team manager: effects on players' performance and perceptions of users. | 1 | 0 | 1 | 1 | 1 | 1 | 0 | 1 | Participants |
| Konttinen et al. (2004) The effects of augmented auditory feedback on psychomotor skill learning in precision shooting | 1 |  | 0 |  |  |  |  |  | Intervention |
| Kovacs et al. (2018) Thinking Outside the Block: External Focus of Attention Improves Reaction Times and Movement Preparation Times in Collegiate Track Sprinters | 1 |  | 0 |  |  |  |  |  | Intervention |
| Kozina et al. (2019) The Effectiveness of Autogenic Training in the Preparation of Elite Athletes in Rhythmic Gymnastics | 1 | 0 | 1 | 1 | 1 | 1 | 0 | 1 | Participants |
| Kraeutner et al. (2020) Leveraging the effector independent nature of motor imagery when it is paired with physical practice | 1 | 0 | 1 | 1 | 1 | 1 | 0 | 1 | Participants |
| Kuan et al. (2017) Effects of music on arousal during imagery in elite shooters: A pilot study. | 1 | 1 | 1 | 1 | 1 | 0 | 0 | 1 | Outcome |
| Laaksonen et al. (2011) Evidence of improved shooting precision in biathlon after 10 weeks of combined relaxation and specific shooting training | 1 | 0 | 1 | 1 | 1 | 1 | 0 | 1 | Participants |
| Lambert & Csikszentmihalyi (2020) Facilitating or foiling flow: the role of momentary perceptions of feedback | 1 |  | exp1: 0; exp2: 0 |  |  |  |  |  | exp1: Intervention exp2: Intervention |
| Lambert et al. (1999) Gymnasts in training: The differential effects of self- and coach-set goals as a function of locus of control | 1 | 0 | 1 | 1 | 0 | 0 | 0 | 1 | Participants + Design + Outcome |
| Land & Tenenbaum (2012) An outcome- and process-oriented examination of a golf-specific secondary task strategy to prevent choking under pressure | 1 | 0 | 1 | 1 | 1 | 1 | 0 | 1 | Participants |
| Land et al. (2013) Examination of Visual Information as a Mediator of External Focus Benefits | 1 | 0 | 1 | 1 | 1 | 1 | 0 | 1 | Participants |
| Landau et al. (2001) Mental simulation increases physical performance estimates but not physical performance | 1 | exp1: 0; exp2: 0 | exp1: 1; exp2: 1 | exp1: 1; exp2: 1 | exp1: 1; exp2: 1 | exp1: 1; exp2: 1 | exp1: 0; exp2: 0 | exp1: 1; exp2: 1 | exp1: Participants; exp2: Participants |
| Landin & Mcdonald (1990) Improving the overheads of collegiate tennis players | 1 |  | 0 |  |  |  |  |  | Intervention |
| Lane & Streeter (2003) The effectiveness of goal setting as a strategy to improve basketball shooting performance | 1 | 0 | 1 | 1 | 1 | 1 | 0 | 1 | Participants |
| Lane et al. (2016) How should I regulate my emotions if I want to run faster? | 1 | 0 | 1 | 1 | 1 | 1 | 0 | 1 | Participants |
| Lane et al. (2016) The effects of brief online self-help intervention strategies on emotions and satisfaction with running performance | 1 | 0 | 1 | 1 | 1 | 0 | 1 | 1 | Participants |
| Larsson et al. (1988) A time and cost efficient stress inoculation training program for athletes: A study of junior golfers | 1 | 0 | 1 | 1 | 1 | 1 | 0 | 1 | Participants |
| Latinjak et al. (2010) Studying the effects of self-talk on thought content with male adult tennis players | 1 | 0 | 1 | 1 | 1 | 0 | 0 | 1 | Participants + Outcome |
| Latinjak et al. (2011) Combining self talk and performance feedback: Their effectiveness with adult tennis players | 1 | 0 | 1 | 1 | 1 | 1 | 1 | 1 | Participants |
| Latinjak et al. (2018) Effects of Reflection to Improve Goal-Directed Self-Talk on Endurance Performance. | 1 | 0 | 1 | 1 | 1 | 1 | 0 | 1 | Participants |
| Lautenbach et al. (2015) Nonautomated pre-performance routine in tennis: An intervention study | 1 | 0 | 1 | 1 | 1 | 1 | 0 | 1 | Participants |
| Lautenbach et al. (2019) Give Me Five? Examining the Psychophysiological Effects of High-Fives in Athletes | 1 |  | 0 |  |  |  |  |  | Intervention |
| Lawrence et al. (2020) Lets go surfing now, everybody's learning how; attentional strategies on expert and novice surfing performance under both practice and competition conditions | 1 |  | exp1: 0; exp2: 0 |  |  |  |  |  | exp1: Intervention exp2: Intervention |
| Lebeau et al. (2018) Is failing the key to success? A randomized experiment investigating goal attainment effects on cognitions, emotions, and subsequent performance. | 1 | 0 | 1 | 1 | 1 | 1 | 0 | 1 | Participants |
| Lee (1990) Psyching up for a muscular endurance task: Effects of image content on performance and mood state | 1 | exp1: 0 exp2: 0 | exp1: 1 exp2: 1 | exp1: 1 exp2: 1 | exp1: 1 exp2: 1 | exp1: 1 exp2: 1 | exp1: 0 exp2: 0 | exp1: 0 exp2: 1 | exp1: Participants exp2: Participants |
| Lee & Hewitt (1987) Using visual imagery in a flotation tank to improve gymnastic performance and reduce physical symptoms | 1 | 0 | 1 | 1 | 1 | 1 | 0 | 1 | Participants |
| Lee et al. (2015) Developing and Instructing Pre-Performance Routines for Tenpin Bowling Competitions (1) | 1 | 1 | 1 | 1 | 0 | 1 | 0 | 1 | Design |
| Leitzelar et al. (2020) Regulatory Fit: Impact on Anxiety, Arousal, and Performance in College-Level Soccer Players | 1 | 0 | 1 | 1 | 1 | 1 | 0 | 1 | Participants |
| Lejeune et al. (1994) Mental rehearsal in table tennis performance | 1 | 0 | 1 | 1 | 1 | 1 | 0 | 1 | Participants |
| Letafatkar et al. (2020) Training athletes with an external attentional focus enhances athletic performance during countermovement jump | 1 | 0 | 1 | 1 | 1 | 1 | 0 | 1 | Participants |
| Lever et al. (2020) A Combined Sleep Hygiene and Mindfulness Intervention to Improve Sleep and Well-Being During High-Performance Youth Tennis Tournaments | 1 | 0 | 1 | 1 | 1 | 1 | 1 | 1 | Participants |
| Li et al. (2021) What Makes an Elite Shooter and Archer? The Critical Role of Interoceptive Attention | 1 | 1 | 1 | 1 | 1 | 0 | 0 | 1 | Outcome |
| Lidor et al. (2013) The effect of attention allocation instructions on self-paced task performance under quiet and distracted conditions | 1 | 0 | 1 | 1 | 1 | 1 | 0 | 1 | Participants |
| Lin et al. (2021) Influence of Imagery Training on Adjusting the Pressure of Fin Swimmers, Improving Sports Performance and Stabilizing Psychological Quality | 1 |  | 0 |  |  |  |  |  | Intervention |
| Lindsay et al. (2020) Effects of personalised motor imagery on the development of a complex weightlifting movement | 1 | 0 | 1 | 1 | 1 | 1 | 0 | 1 | Participants |
| Liu et al. (2021) Examining the Effects of Brief Mindfulness Training on Athletes' Flow: The Mediating Role of Resilience | 1 | 1 | 1 | 1 | 1 | 0 | 0 | 1 | Outcome |
| Lobmeyer & Wasserman (1986) Preliminaries to free throw shooting: Superstitious behavior? | 1 |  | 0 |  |  |  |  |  | Intervention |
| Lohasz & Leith (1997) The effect of three mental preparation strategies on the performance of a complex response time task | 1 | 1 | 1 | 1 | 1 | 0 | 0 | 1 | Outcome |
| Lohse (2012) The influence of attention on learning and performance: pre-movement time and accuracy in an isometric force production task | 1 | 0 | 1 | 1 | 1 | 1 | 0 | 1 | Participants |
| Lohse et al. (2010) How changing the focus of attention affects performance, kinematics, and electromyography in dart throwing | 1 | 0 | 1 | 1 | 1 | 1 | 0 | 1 | Participants |
| LoSarah et al. (2021) Effects of attentional focus cues on lower extremity kinematics during inside of the foot soccer trap among expert soccer players | 1 |  | 0 |  |  |  |  |  | Intervention |
| Lotfi et al. (2020) Effect of positive and negative dimensions of mental imagery and self-talk on learning of soccer kicking skill | 1 |  | 0 |  |  |  |  |  | Intervention |
| Lowe (1989) The sport psychology program of the USA Womens National Volleyball Team | 1 |  | 0 |  |  |  |  |  | Intervention |
| Lundgren et al. (2020) Acceptance and commitment training to promote psychological flexibility in ice hockey performance: A controlled group feasibility study | 1 | 1 | 1 | 1 | 1 | 0 | 0 | 1 | Outcome |
| Lundgren et al. (2021) Acceptance and Commitment Training for Ice Hockey Players: A Randomized Controlled Trial | 1 | 0 | 1 | 1 | 1 | 1 | 1 | 1 | Participants |
| Lutz et al. (2001) Procedural variables and skill level influences on pre-performance mental practice efficacy | 1 | exp1: 0; exp2: 0 | exp1: 1 | exp1: 1; exp2: 1 | exp1: 1; exp2: 1 | exp1: 1; exp2: 1 | exp1: 0; exp2: 0 | exp1: 1; exp2: 1 | exp1: Participants; exp2: Participants |
| MacDonald & Minahan (2018) Mindfulness training attenuates the increase in salivary cortisol concentration associated with competition in highly trained wheelchair-basketball players | 1 | 1 | 1 | 1 | 1 | 0 | 0 | 1 | Outcome |
| MacDonald et al. (2018) An evaluation of the effects of mindfulness training from the perspectives of wheelchair basketball players | 1 | 1 | 1 | 1 | 1 | 0 | 0 | 0 | Outcome |
| Machac & Machacova (1985) Some methodological aspects of using relaxation-activation intervention in training for an "important contest" | 1 |  | 0 |  |  |  |  |  | Intervention |
| Makaruk et al. (2013) Acute effects of attentional focus on shot put performance in elite athletes | 1 | 0 | 1 | 1 | 1 | 1 | 0 | 0 | Participants |
| Makaruk et al. (2019) The effects of combining focus of attention and autonomy support on shot accuracy in the penalty kick | 1 | 0 | 1 | 1 | 1 | 1 | 0 | 1 | Participants |
| Makaruk et al. (2020) Optimizing the penalty kick under external focus of attention and autonomy support instructions | 1 | 0 | 1 | 1 | 1 | 1 | 0 | 0 | Participants |
| Males et al. (2018) Application of an innovative performance demand model with canoe slalom athletes and their coach | 1 | 0 | 1 | 1 | 1 | 0 | 0 | 0 | Participants + Design + Outcome |
| Mallett & Hanrahan (1997) Race modeling: An effective cognitive strategy for the 100 m sprinter | 1 |  | 0 |  |  |  |  |  | Intervention |
| Malouff & Murphy (2006) Effects of Self-Instructions on Sport Performance | 1 | 0 | 1 | 1 | 1 | 1 | 1 | 0 | Participants |
| Malouff et al. (2008) Effects of pre-competition positive imagery and self-instructions on accuracy of serving in tennis | 1 | 0 | 1 | 1 | 1 | 1 | 0 | 1 | Participants |
| Marshall & Gibson (2017) The Effect of an Imagery Training Intervention on Self-confidence, Anxiety and Performance in Acrobatic Gymnastics - A Pilot Study | 1 | 0 | 1 | 1 | 1 | 1 | 0 | 1 | Participants |
| Marshall et al. (2016) The Effects of Self-Talk Cues on the Putting Performance of Golfers Susceptible to Detrimental Putting Performances Under High Pressure Settings | 1 | 0 | 1 | 1 | 1 | 1 | 0 | 1 | Participants |
| Martin & Toogood (1997) Cognitive and behavioral components of a seasonal psychological skills training program for competitive figure skaters | 1 | 0 | 1 | 1 | 1 | 0 | 1 | 1 | Participants |
| Masciana et al (2001) Effects of cognitive strategies on dart throwing performance | 1 | 0 | 1 | 1 | 1 | 1 | 1 | 1 | Participants |
| Maurer & Munzert (2013) Influence of attentional focus on skilled motor performance: performance decrement under unfamiliar focus conditions | 1 | exp1: 0 exp2: 0 | exp1: 1  exp2: 1 | exp1: 1  exp2: 1 | exp1: 1 exp2: 1 | exp1: 1 exp2: 1 | exp1: 0 exp2: 0 | exp1: 1 exp2: 1 | exp1: Participants exp2: Participants |
| Maynard et al. (1998) Stress management in sport: A comparison of unimodal and multimodal interventions | 1 | 0 | 1 | 1 | 1 | 0 | 0 | 1 | Participants + Outcome |
| McCormick et al. (2018) Effects of a Motivational Self-Talk Intervention for Endurance Athletes Completing an Ultramarathon | 1 | 0 | 1 | 1 | 1 | 1 | 0 | 1 | Participants |
| McKenzie & Howe (1991) The effect of imagery on tackling performance in rugby | 1 | 0 | 1 | 1 | 1 | 1 | 0 | 1 | Participants |
| McKenzie & Rushall (1974) Effects of self-recording on attendance and performance in a competitive swimming training environment | 1 | exp1: 0; exp2: 0 | exp1: 1; exp2: 1 | exp1: 0; exp2: 0 | exp1: 3; exp2: 3 | exp1: 0; exp2: 0 | exp1: 0; exp2: 0 | exp1: 1; exp2: 1 | Participants + Not performance enhancement + Design + Outcome |
| McNeil et al. (2019) Imagery training for reactive agility: Performance improvements for decision time but not overall reactive agility | 1 | 0 | 1 | 1 | 1 | 1 | 0 | 1 | Participants |
| McNeill et al. (2021) Self-modelled versus skilled-peer modelled AO plus MI effects on skilled sensorimotor performance: A stage 2 registered report | 1 | 0 | 1 | 1 | 1 | 1 | 0 | 1 | Participants |
| McNeill et al. (2020) Kinaesthetic imagery ability moderates the effect of an AO+MI intervention on golf putt performance: A pilot study | 1 | 0 | 1 | 1 | 1 | 1 | 0 | 1 | Participants |
| Mead et al. (2000) Positive and negative stimuli in relation to tennis players' reaction time | 1 |  | 0 |  |  |  |  |  | Intervention |
| Meggs & Chen (2019) Competitive Performance Effects of Psychological Skill Training for Youth Swimmers | 1 | 0 | 1 | 1 | 1 | 1 | 0 | 1 | Participants |
| Mehrsafar et al. (2019) The effects of mindfulness training on competition-induced anxiety and salivary stress markers in elite Wushu athletes: A pilot study | 1 | 1 | 1 | 1 | 1 | 0 | 0 | 1 | Outcome |
| Mehrsafar et al. (2019) The effects of mindfulness training on competition-induced anxiety and salivary stress markers in elite Wushu athletes: A pilot study | 1 | 1 | 1 | 1 | 1 | 0 | 0 | 1 | Outcome |
| Meijen et al. (2022) "Short and Sweet": A Randomized Controlled Initial Investigation of Brief Online Psychological Interventions With Endurance Athletes | 1 | 0 | 1 | 1 | 1 | 0 | 1 | 1 | Participants |
| Meixner & Herbert (2021) Does Attentional Focus Influence Psychophysiological Responses to an Acute Bout of Exercise? Evidence From an Experimental Study Using a Repeated-Measures Design | 1 | 0 | 1 | 1 | 1 | 0 | 0 | 1 | Participants + Outcome |
| Mellalieu et al. (2006) The effects of goal setting on rugby performance | 1 | 1 | 1 | 1 | 0 | 1 | 0 | 1 | Design |
| Memmert (2007) Can creativity be improved by an attention-broadening training program? An exploratory study focusing on team sport | 1 |  | 0 |  |  |  |  |  | Intervention |
| Memmert et al. (2020) The role of different directions of attention on the extent of implicit perception in soccer penalty kicking | 1 |  | 0 |  |  |  |  |  | Intervention |
| Memmert & Orliczek (2013) Decide like Lionel Messi! The impact of regulatory focus on divergent thinking in sports | 1 | 0 | 1 | 1 | 1 | 0 | 0 | 1 | Participants + Outcome |
| Mertens et al. (2018) An experiment on the impact of coaches' and athlete leaders' competence support on athletes' motivation and performance | 1 |  | 0 |  |  |  |  |  | Intervention |
| Mertens et al. (2020) Leading together towards a stronger 'us': An experimental test of the effectiveness of the 5R Shared Leadership Program (5RS) in basketball teams | 1 |  | 0 |  |  |  |  |  | Intervention |
| Mesagno et al. (2019) Primed to perform: Comparing different pre-performance routine interventions to improve accuracy in closed, self-paced motor tasks | 1 | exp2: 0 | exp2: 1 | exp2: 1 | exp2: 1 | exp2: 1 | exp2: 0 | exp2: 1 | exp1: Not related to sport psychology exp2: Participants |
| Mesagno & Mullane-Grant (2010) A comparison of different pre-performance routines as possible choking interventions | 1 | 0 | 1 | 1 | 1 | 1 | 0 | 1 | Participants |
| Mesagno et al. (2015) Examining the accuracy and in-game performance effects between pre- and post-performance routines: A mixed methods study | 1 | 0 | 1 | 1 | 1 | 1 | 0 | 1 | Participants |
| Miller (1993) Efficacy strength and performance in competitive swimmers of different skill levels | 1 |  | 0 |  |  |  |  |  | Intervention |
| Miller & Donohue (2003) The development and controlled evaluation of athletic mental preparation strategies in high school distance runners | 1 | 0 | 1 | 1 | 1 | 1 | 0 | 1 | Participants |
| Miller & McAuley (1987) Effects of a goal-setting training program on basketball free-throw self-efficacy and performance | 1 | 0 | 1 | 1 | 1 | 1 | 1 | 1 | Participants |
| Mills et al. (2005) The effect of a 10-week training regimen on lumbo-pelvic stability and athletic performance in female athletes: A randomized-controlled trial | 1 |  | 0 |  |  |  |  |  | Intervention |
| Mîndrescu & Popescu (2017) The Evaluation Of Sensorial Perception Dominated Through Training In Sports Performances | 1 | 0 | 1 | 1 | 1 | 0 | 0 | 1 | Participants + Outcome |
| Miserandino (1998) Attributional retraining as a method of improving athletic performance | 1 | 0 | 1 | 1 | 1 | 1 | 0 | 1 | Participants |
| Mize-Climer et al. (2021) Behavioral interventions to improve the performance of collegiate volleyball | 1 | 1 | 1 | 1 | 0 | 1 | 0 | 1 | Design |
| Moen et al. (2015) The effects from mindfulness training on Norwegian junior elite athletes in sport | 1 | 0 | 1 | 1 | 1 | 0 | 1 | 1 | Participants |
| Moen et al. (2016) The effects of attention training techniques on stress and performance in sports | 1 | 0 | 1 | 1 | 1 | 0 | 1 | 1 | Participants |
| Moen et al. (2018) The effects of Perceptual-Cognitive training on Subjective Performance in Elite Athletes | 1 |  | 0 |  |  |  |  |  | Intervention |
| Moghadam et al. (2013) Impact assessment of mindfulness techniques education on anxiety and sports performance in Badminton players Isfahan | 1 | 1 | 1 | 1 | 1 | 0 | 0 | 1 | Outcome |
| Mohd Zahir et al. (2016) Enhanced motor ability, coordination and psychobiological competence in predicting high soccer performance | 1 | 1 | 1 | 1 | 1 | 0 | 0 | 1 | Outcome |
| Moore et al. (2019) Increasing Collegiate Strength and Conditioning Coaches' Communication of Training Performance and Process Goals With Athletes | 1 |  | 0 |  |  |  |  |  | Intervention |
| Morais & Gomes (2019) Pre-service routines, mental toughness and performance enhancement of young tennis athletes | 1 | 0 | 1 | 1 | 1 | 1 | 0 | 1 | Participants |
| Moreno et al. (2016) An Intervention Based on Video Feedback and Questioning to Improve Tactical Knowledge in Expert Female Volleyball Players | 1 |  | 0 |  |  |  |  |  | Intervention |
| Morgan et al. (1983) Facilitation of physical performance by means of a cognitive strategy | 1 | 0 | 1 | 1 | 1 | 1 | 0 | 1 | Participants |
| Mosewich et al. (2013) Applying Self-Compassion in Sport: An Intervention with Women Athletes | 1 | 1 | 1 | 1 | 1 | 0 | 0 | 1 | Outcome |
| Mullen & Hardy (2010) Conscious processing and the process goal paradox | 1 |  | exp1: 0; exp2: 0; exp3: 0 |  |  |  |  |  | exp1: Intervention; exp2: Intervention; exp3: Intervention |
| Murgia et al. (2014) Using perceptual home-training to improve anticipation skills of soccer goalkeepers | 1 |  | 0 |  |  |  |  |  | Intervention |
| Murphy & Woolfolk (1987) The effects of cognitive interventions on competitive anxiety and performance on a fine motor skill accuracy task | 1 | 0 | 1 | 1 | 1 | 1 | 0 | 1 | Participants |
| Müller et al. (2021) The relationship of motive disposition and situational incentives to individual differences in choking under pressure | 1 |  | 0 |  |  |  |  |  | Intervention |
| Navarro et al. (2012) The effects of high pressure on the point of no return in simulated penalty kicks | 1 |  | 0 |  |  |  |  |  | Intervention |
| Nien et al. (2020) Mindfulness Training Enhances Endurance Performance and Executive Functions in Athletes: An Event-Related Potential Study | 1 | 0 | 1 | 1 | 1 | 1 | 0 | 1 | Participants |
| Nougier et al. (1990) Covert orienting of attention and motor preparation processes as a factor of succes in fencing (Orientation de l'attention et processus de preparation motrice en tant que facteurs de succes en escrime) | 1 |  | exp1: 0; exp2: 0 |  |  |  |  |  | exp1: Intervention exp2: Intervention |
| Núñez et al. (2010) Effects of providing advance cues during a soccer penalty kick on the kicker's rate of success | 1 |  | 0 |  |  |  |  |  | Intervention |
| O & Munroe-Chandler (2008) The effects of image speed on the performance of a soccer task | 1 | 0 | 1 | 1 | 1 | 1 | 0 | 1 | Participants |
| Ojaghi et al. (2013) The effect of mindfulness techniques training on anxiety and sport performance among table tennis players. | 1 |  | 0 |  |  |  |  |  | Intervention |
| Olmedilla et al. (2019) Psychological Intervention Program to Control Stress in Youth Soccer Players | 1 | 0 | 1 | 1 | 1 | 0 | 0 | 1 | Participants + Outcome |
| Olsson et al. (2008) Internal imagery training in active high jumpers | 1 | 0 | 1 | 1 | 1 | 1 | 0 | 0 | Participants |
| Onestak (1997) The effect of visuo-motor behavior rehearsal (VMBR) and videotaped modeling (VM) on the free-throw performance of intercollegiate athletes | 1 | 0 | 1 | 1 | 1 | 1 | 0 | 1 | Participants |
| Orbach et al. (1997) Changing attributions with an attribution training technique related to basketball dribbling | 1 | 0 | 1 | 1 | 1 | 1 | 0 | 1 | Participants |
| Ortega & Wang (2018) Effectiveness of an integrated mental skills and biofeedback training program on sport shooters | 1 |  | 0 |  |  |  |  |  | Intervention |
| Ottoboni et al. (2014) Just do it: Embodied experiences improve Taekwondo athletes sport performance | 1 | 0 | 1 | 1 | 1 | 1 | 0 | 0 | Participants |
| Oxhandler (2017) Mindfulness Intervention With a U.S. Womens NCAA Division I Basketball Team: Impact on Stress, Athletic Coping Skills and Perceptions of Intervention | 1 | 1 | 1 | 1 | 1 | 0 | 0 | 1 | Outcome |
| Panchuk et al. (2018) Exploring the Effectiveness of Immersive Video for Training Decision-Making Capability in Elite, Youth Basketball Players | 1 |  | 0 |  |  |  |  |  | Intervention |
| Papanikolaou et al. (2012) The effects of a psychological skills training program on the cohesion of a men’s soccer team | 1 | 1 | 1 | 1 | 1 | 0 | 0 | 1 | Outcome |
| Parker et al. (2016) Enhancing the academic success of competitive student athletes using a motivation treatment intervention (Attributional Retraining) | 1 | 1 | 1 | 0 | 1 | 0 | 0 | 1 | Not performance enhancement + Outcome |
| Patsiaouras (2008) Person-centered support and athletes' motivation for performance | 1 | 0 | 1 | 1 | 1 | 0 | 0 | 1 | Participants + Outcome |
| Pelusco et al. (2005) A comparison of mental strategies during athletic skills performance | 1 | 0 | 1 | 1 | 1 | 1 | 0 | 1 | Participants |
| Perkins-Ceccato et al. (2003). Effects of focus of attention depend on golfers' skill | 1 | 0 | 1 | 1 | 1 | 1 | 0 | 0 | Participants |
| Perry et al. (2017) Efficacy of a Brief Mindfulness Intervention to Prevent Athletic Task Performance Deterioration: A Randomized Controlled Trial | 1 | 0 | 1 | 1 | 1 | 1 | 0 | 1 | Participants |
| Pettit & Karageorghis (2020) Effects of video, priming, and music on motivation and self-efficacy in American football players | 1 | 0 | 1 | 1 | 1 | 0 | 0 | 1 | Participants + Outcome |
| Phipps & Morehouse (1969) Effects of mental practice on the acquisition of motor skills of varied difficulty | 1 | 0 | 1 | 1 | 1 | 1 | 0 | 0 | Participants |
| Pierce & Burton (1998) Scoring the perfect 10: Investigating the impact of goal-setting styles on a goal-setting program for female gymnasts | 1 | 0 | 1 | 1 | 1 | 1 | 0 | 1 | Participants |
| Pierce et al. (1993) Effects of Progressive Relaxation on Maximal Muscle Strength and Power | 1 | 1 | 1 | 1 | 1 | 0 | 0 | 1 | Outcome |
| Pineau et al. (2019) From losing record to championship season: A case study of mindful sport performance enhancement | 1 | 1 | 1 | 1 | 1 | 0 | 0 | 1 | Outcome |
| Pizzera et al. (2017) Long-term effects of acoustic reafference training (ART) | 1 |  | 0 |  |  |  |  |  | Intervention |
| Pocock et al. (2010) Precision teaching and fluency: The effects of charting and goal-setting on skaters' performance | 1 | exp3: 0 | exp1: 0; exp2: 0; exp3: 1 | exp3: 1 | exp3: 0 | exp3: 1 | exp3: 0 | exp3: 0 | exp1: Intervention; exp2: Intervention; exp3: Participants + Design |
| Pocock et al. (2019) Using an imagery intervention to train visual exploratory activity in elite academy football players | 1 | 0 | 1 | 1 | 0 | 1 | 1 | 1 | Participants + Design |
| Porter & Sims (2013) Altering focus of attention influences elite athletes sprinting performance | 1 |  | 0 |  |  |  |  |  | Intervention |
| Porter et al. (2010) Standing long-jump performance is enhanced when using an externalfocus of attention | 1 |  | 0 |  |  |  |  |  | Intervention |
| Porter et al. (2012) Increasing the distance of an external focus of attention enhances standing long jump performance | 1 |  | 0 |  |  |  |  |  | Intervention |
| Porter et al. (2013) Instructing Skilled Athletes to Focus Their Attention Externally at Greater Distances Enhances Jumping Performance | 1 |  | 0 |  |  |  |  |  | Intervention |
| Porter et al. (2015) Adopting an external focus of attention improves sprinting performance in low-skilled sprinters | 1 |  | 0 |  |  |  |  |  | Intervention |
| Predebon & Docker (1992) Free-throw shooting performance as a function of preshot routines | 1 | 0 | 1 | 1 | 1 | 1 | 0 | 0 | Participants |
| Pusenjak et al. (2015) Can biofeedback training of psychophysiological responses enhance athletes' sport performance? A practitioner's perspective | 1 |  | 0 |  |  |  |  |  | Intervention |
| Quevedo et al. (1999) Experimental study of visual training effects in shooting initiation | 1 | 0 | 1 | 1 | 1 | 1 | 0 | 1 | Participants |
| Quinton et al. (2018) Imagery meaning and content in golf: Effects on performance, anxiety, and confidence | 1 | 0 | 1 | 1 | 1 | 1 | 0 | 1 | Participants |
| Raab et al. (2005) Improving the 'how' and 'what' decisions of elite table tennis players | 1 |  | 0 |  |  |  |  |  | Intervention |
| Raj et al. (2021) Association between Hamstring Flexibility and Sprint Speed after 8 Weeks of Yoga in Male Rugby Players | 1 |  | 0 |  |  |  |  |  | Intervention |
| Raju et al. (1994) Comparison of effects of yoga & physical exercise in athletes | 1 | 1 | 1 | 1 | 1 | 0 | 0 | 1 | Outcome |
| Ramsey et al. (2008) Exploring a modified conceptualization of imagery direction and golf putting performance | 1 | 0 | 1 | 1 | 1 | 1 | 0 | 1 | Participants |
| Ramsey et al. (2010) Examining the emotion aspect of PETTLEP-based imagery with penalty taking in soccer | 1 | 0 | 1 | 1 | 1 | 1 | 1 | 1 | Participants |
| Rao et al. (2021) Impact of yoga in facilitating muscular functioning among asymptomatic male cricket players: Longitudinal randomized controlled study | 1 |  | 0 |  |  |  |  |  | Intervention |
| Rathschlag & Memmert (2015) Self-Generated Emotions and Their Influence on Sprint Performance: An Investigation of Happiness and Anxiety | 1 | exp1: 0; exp2: 0 | exp1: 1; exp2: 1 | exp1: 1; exp2: 1 | exp1: 1; exp2: 1 | exp1: 1; exp2: 1 | exp1: 0; exp2: 0 | exp1: 1; exp2: 1 | exp1: Participants exp2: Participants |
| Razon et al. (2014). Effects of imagery on effort perception and cycling endurance. Journal of Imagery Research in Sport and Physical Activity | 1 | 0 | 1 | 1 | 1 | 1 | 0 | 1 | Participants |
| Rebner et al. (2021) Talking Your Way to Record Times: The Effects of Instructional and Motivational Self-Talk on 10km Time-Trial Running Performance | 1 | 0 | 1 | 1 | 1 | 1 | 0 | 1 | Participants |
| Rhodes et al. (2018) Enhancing Grit Through Functional Imagery Training in Professional Soccer | 1 | 1 | 1 | 1 | 1 | 0 | 0 | 1 | Outcome |
| Rijken et al. (2016) Increasing Performance of Professional Soccer Players and Elite Track and Field Athletes with Peak Performance Training and Biofeedback: A Pilot Study | 1 |  | 0 |  |  |  |  |  | Intervention |
| Ring et al. (2019) Effects of antisocial behaviour on opponent's anger, attention, and performance | 1 | 0 | 1 | 1 | 1 | 1 | 0 | 1 | Participants |
| Robin et al. (2007) Effects of motor imagery training on service return accuracy in tennis: The role of imagery ability | 1 | 0 | 1 | 1 | 1 | 1 | 0 | 0 | Participants |
| Robin et al. (2019) Free throw performance in non-expert basketball players: The effect of dynamic motor imagery combined with action observation | 1 | 0 | 1 | 1 | 1 | 1 | 0 | 1 | Participants |
| Rodgers et al. (1991) The effect of an imagery training program on imagery ability, imagery use, and figure skating performance | 1 | 0 | 1 | 1 | 1 | 1 | 0 | 1 | Participants |
| Rogerson & Hrycaiko (1998) Enhancing competitive performance of ice hockey goaltenders using centering and self-talk | 1 | 0 | 1 | 1 | 0 | 1 | 0 | 1 | Participants + Design |
| Romano-Smith et al. (2018) Simultaneous and alternate action observation and motor imagery combinations improve aiming performance | 1 | 0 | 1 | 1 | 1 | 1 | 0 | 1 | Participants |
| Romano Smith et al. (2019) The effect of action observation and motor imagery combinations on upper limb kinematics and EMG during dart-throwing | 1 | 0 | 1 | 1 | 1 | 1 | 0 | 1 | Participants |
| Romeas et al. (2016) 3D-Multiple Object Tracking training task improves passing decision-making accuracy in soccer players | 1 |  | 0 |  |  |  |  |  | Intervention |
| Romeas et al. (2019) Combining 3D-MOT with sport decision-making for perceptual-cognitive training in virtual reality | 1 |  | exp1: 0; exp2: 0 |  |  |  |  |  | exp1: Intervention exp2: Intervention |
| Rooks et al. (2017) "We Are Talking About Practice": the Influence of Mindfulness vs. Relaxation Training on Athletes' Attention and Well-Being over High-Demand Intervals | 1 | 1 | 1 | 1 | 1 | 0 | 0 | 1 | Outcome |
| Ruiz et al. (2021) Self-Regulation in High-Level Ice Hockey Players: An Application of the MuSt Theory | 1 | 0 | 1 | 1 | 1 | 0 | 1 | 1 | Participants |
| Röthlin et al. (2020) Differential and shared effects of psychological skills training and mindfulness training on performance-relevant psychological factors in sport: a randomized controlled trial | 1 | 0 | 1 | 1 | 1 | 0 | 0 | 1 | Outcome |
| Rusciano et al. (2017) Neuroplus biofeedback improves attention, resilience, and injury prevention in elite soccer players | 1 |  | 0 |  |  |  |  |  | Intervention |
| Rushall & Shewchuk (1989) Effects of thought content instructions on swimming performance | 1 | 0 | 1 | 1 | 1 | 1 | 0 | 1 | Participants |
| Rushall et al. (1988) Effects of three types of thought content instructions on skiing performance | 1 | 0 | 1 | 1 | 1 | 1 | 0 | 1 | Participants |
| Rushall et al. (1998) The role of imagery in physical performance | 1 |  | 0 |  |  |  |  |  | Intervention |
| Rymal & Ste-Marie (2017) Imagery Ability Moderates the Effectiveness of Video Self Modeling on Gymnastics Performance | 1 | 0 | 1 | 1 | 1 | 1 | 0 | 1 | Participants |
| Saemi et al. (2017) The interaction of external/internal and relevant/irrelevant attentional focus on skilled performance: the mediation role of visual information | 1 | 0 | 1 | 1 | 1 | 1 | 0 | 0 | Participants |
| Salama (2013) Development of psychological skills for success in vault table | 1 | 0 | 1 | 1 | 1 | 0 | 1 | 1 | Participants |
| Savoy (1997) Two individualized mental training programs for a team sport | 1 | 1 | 1 | 1 | 0 | 1 | 0 | 1 | Design |
| Schomer (1987) Mental strategy training programme for marathon runners. | 1 | 0 | 1 | 1 | 0 | 0 | 0 | 0 | Participants + Design + Outcome |
| Schuler & Langens (2007) Psychological Crisis in a Matathon and the Buffering Effects of Self-Verbalizations | 1 | exp2: 0 | exp1: 0; exp2: 1 | exp2: 1 | exp2: 1 | exp2: 1 | exp2: 0 | exp2: 1 | exp1: Intervention  exp2: Participants |
| Schuler et al. (2017) Affiliation-related goal instructions The secret of sporting success for affiliation motivated individuals | 1 | 0 | 1 | 1 | 1 | 0 | 0 | 1 | Participants + Outcome |
| Schutts et al. (2017) Does focus of attention improve snatch lift kinematics? | 1 |  | 0 |  |  |  |  |  | Intervention |
| Schücker et al. (2009) The effect of attentional focus on running economy | 1 | 0 | 1 | 1 | 1 | 0 | 0 | 1 | Participants + Outcome |
| Schücker et al. (2013) On the Optimal Focus of Attention for Efficient Running at High Intensity | 1 | 0 | 1 | 1 | 1 | 0 | 0 | 1 | Participants + Outcome |
| Schücker et al. (2015) Don’t Think about Your Movements: Effects of Attentional Instructions on Rowing Performance | 1 | 0 | 1 | 1 | 1 | 0 | 0 | 1 | Participants + Outcome |
| Schücker & Parrington (2019) Thinking about your running movement makes you less efficient: Attentional focus effects on running economy and kinematics | 1 | 0 | 1 | 1 | 1 | 0 | 0 | 1 | Participants + Outcome |
| Scott (1984) Hypnotherapy training for basketball: An experimental approach | 1 | 1 | 1 | 1 | 1 | 0 | 0 | 1 | Outcome |
| Scott-Hamilton et al. (2016) Effects of a mindfulness intervention on sports-anxiety, pessimism, and flow in competitive cyclists. | 1 | 0 | 1 | 1 | 1 | 0 | 0 | 1 | Participants + Outcome |
| Seabourne et al. (1984) Effect of individualized practice and training of visuo-motor behavior rehearsal in enhancing karate performance. / Effet de la pratique et de l ' entrainement individuel d ' un exercice de conduite visuo-motrice sur l ' amelioration de la performance en karate | 1 | 0 | 1 | 1 | 1 | 0 | 1 | 1 | Participants |
| Sesum & Kajtna (2018) The use of audiovisual stimulation in learning gymnastic elements | 1 |  | 0 |  |  |  |  |  | Intervention |
| Sewell (1996) Attention-focusing instructions and training times in competitive youth swimmers | 1 | 0 | 1 | 1 | 1 | 1 | 0 | 1 | Participants |
| Shaabani et al. (2020) Does a brief mindfulness intervention counteract the detrimental effects of ego depletion in basketball free throw under pressure? | 1 | 0 | 1 | 1 | 1 | 1 | 0 | 1 | Participants |
| Shackell & Standing (2007) Mind Over Matter: Mental Training Increases Physical Strengh | 1 | 1 | 1 | 1 | 1 | 0 | 0 | 1 | Outcome |
| Sheard & Golby (2006) Effect of a Psychological Skills Training Program on Swimming Performance and Positive Psychological Development | 1 | 0 | 1 | 1 | 1 | 1 | 1 | 1 | Participants |
| Shepelenko (2017) Integral technologies of psycho-physical training of athletes in sports aerobics | 1 |  | 0 |  |  |  |  |  | Intervention |
| Sherwood et al. (2014) Judging joint angles and movement outcome: Shifting the focus of attention in dart-throwing | 1 | exp1: 0  exp2: 0 | exp1: 1  exp2: 1 | exp1: 1  exp2: 1. | exp1: 1 exp2: 1 | exp1: 1 exp2: 1 | exp1: 0 exp2: 0 | exp1: 1 exp2: 1 | exp1: Participants  exp2: Participants |
| Shick (1970) Effects of mental practice on selected volleyball skills for college women | 1 | exp1: 0 exp2: 0 exp3: 0 | exp1: 1 exp2: 1 exp3: 1 | exp1: 1 exp2: 1 exp3: 1 | exp1: 1 exp2: 1 exp3: 1 | exp1: 1 exp2: 1 exp3: 1 | exp1: 0 exp2: 0 exp3: 0 | exp1: 0 exp2: 0 exp3: 1 | exp1: Participants exp2: Participants exp3: Participants |
| Short et al. (2002) The effect of imagery function and imagery direction on self-efficacy and performance on a golf-putting task | 1 | 0 | 1 | 1 | 1 | 1 | 0 | 1 | Participants |
| Sidaway & Trzaska (2005) Can mental practice increase ankle dorsiflexor torque? | 1 | 0 | 1 | 1 | 1 | 1 | 0 | 0 | Participants |
| Simek et al. (1994) Contracting and chaining to improve the performance of a college golf team: improvement and deterioration | 1 | 1 | 0 | 1 | 1 | 1 | 0 | 0 | Intervention |
| Simonsmeier et al. (2018) The Effects of Motor Imagery Training on Performance and Mental Representation of 7-to 15-Year-Old Gymnasts of Different Levels of Expertise | 1 | 0 | 1 | 1 | 1 | 1 | 0 | 1 | Participants |
| Singer (1986) Sports performance: A five-step mental approach | 1 |  | 0 |  |  |  |  |  | Intervention |
| Singer et al. (1994) Training mental quickness in beginning/intermediate tennis players | 1 | 0 | 1 | 1 | 1 | 1 | 1 | 0 | Participants |
| Singer et al. (2001) The influence of a process versus an outcome orientation on tennis performance and knowledge | 1 | 0 | 1 | 1 | 1 | 1 | 0 | 1 | Participants |
| Slimani et al. (2017) Effects of mental training on muscular force, hormonal and physiological changes in kickboxers | 1 | 1 | 1 | 1 | 1 | 0 | 0 | 1 | Outcome |
| Smeeton et al. (2013) Can imagery facilitate improvements in anticipation behavior? | 1 | 0 | 1 | 1 | 1 | 0 | 0 | 1 | Participants + Outcome |
| Smith & Ward (2006) Behavioral interventions to improve performance in collegiate football | 1 | 1 | 1 | 1 | 0 | 1 | 0 | 1 | Design |
| Solberg et al. (1995) Meditation: a modulator of the immune response to typical stress? A brief report | 1 | 0 | 1 | 1 | 1 | 0 | 0 | 1 | Participants + Outcome |
| Solberg et al. (2000) Stress reactivity to and recovery from a standardised exercise bout: a study of 31 runners practising relaxation techniques | 1 | 0 | 1 | 1 | 1 | 0 | 0 | 1 | Participants + Outcome |
| Sommer et al. (2018) Timing Training in Female Soccer Players: Effects on Skilled Movement Performance and Brain Responses | 1 |  | 0 |  |  |  |  |  | Intervention |
| Son et al. (2011) "I am" versus "we are": effects of distinctive variants of self-talk on efficacy beliefs and motor performance | 1 | 0 | 1 | 1 | 1 | 1 | 0 | 1 | Participants |
| Sopa & Pomohaci (2020) Discovering the anxiety level of a basketball team using the SCAT questionnaire | 1 |  | 0 |  |  |  |  |  | Intervention |
| Sorbie et al. (2019) Effect of a 6-week yoga intervention on swing mechanics during the golf swing: a feasibility study | 1 |  | 0 |  |  |  |  |  | Intervention |
| Soto et al. (2020) Well-Being and Throwing Speed of Women Handball Players Affected by Feedback | 1 |  | 0 |  |  |  |  |  | Intervention |
| Spindler et al. (2019) Motivational-general arousal imagery does not improve decision-making performance in elite endurance cyclist | 1 | 1 | 1 | 1 | 1 | 0 | 0 | 1 | Outcome |
| Spink & Longhurst (1986) Cognitive strategies and swimming performances: an exploratory study | 1 | 0 | 1 | 1 | 1 | 1 | 0 | 0 | Participants |
| Stebbins (1968) A comparison of the effects of physical and mental practice in learning a motor skill | 1 | 0 | 1 | 1 | 1 | 1 | 0 | 0 | Participants |
| Stenzel et al. (2021) Developing and Implementing an App-Based Blended Psychological Skills Training: A Case Study | 1 | 0 | 1 | 1 | 1 | 0 | 0 | 1 | Participants + Outcome |
| Stewart & Hall (2017) The Effects of Cognitive General Imagery Training on Decision-Making Abilities in Curling: A Single-Subject Multiple Baseline Approach | 1 | 1 | 1 | 1 | 0 | 1 | 0 | 1 | Design |
| Stine et al. (2019) Effects of Feedback Type and Personality on 2,000-m Ergometer Performance in Female Varsity Collegiate Rowers | 1 |  | 0 |  |  |  |  |  | Intervention |
| Stoate & Wulf (2011) Does the Attentional Focus Adopted by Swimmers Affect Their Performance? | 1 | 0 | 1 | 1 | 1 | 1 | 0 | 0 | Participants |
| Stocker et al. (2019) Self-control strength and mindfulness in physical exercise performance: Does a short mindfulness induction compensate for the detrimental ego depletion effect? | 1 | 0 | 1 | 1 | 1 | 0 | 0 | 1 | Participants + Outcome |
| Stovba et al. (2018) Effects of stable and variable indicators on sport performance of skilled female ski racers | 1 |  | 0 |  |  |  |  |  | Intervention |
| Straub (1989) The effect of three different methods of mental training on dart throwing performance | 1 | 0 | 1 | 1 | 1 | 1 | 0 | 1 | Participants |
| Suedfeld & Bruno (1990) Flotation REST and imagery in the improvement of athletic performance | 1 | 0 | 1 | 1 | 1 | 1 | 0 | 1 | Participants |
| Suedfeld et al. (1993) Enhancing Perceptual-Motor Accuracy Through Flotation REST | 1 | 0 | 1 | 1 | 1 | 1 | 0 | 0 | Participants |
| Sungkowo et al. (2018) The Role of Training of Goal Setting And Muscle Relaxation to Self Confidence of Swimmer | 1 | 0 | 1 | 1 | 1 | 1 | 0 | 1 | Participants |
| Tammen (1996) Elite Middle and long distance runners associative/dissociative coping | 1 |  | 0 |  |  |  |  |  | Intervention |
| Tan et al. (2016) The perception of elite athletes' guided self-reflection and performance in archery | 1 | 1 | 1 | 1 | 0 | 1 | 0 | 1 | Design |
| Taylor (1998) Training to use an imagery strategy for PK-RNG games | 1 | exp1: 0; exp2: 0 | exp1: 1; exp2: 1 | exp1: 1; exp2: 1 | exp1: 1; exp2: 1 | 0 | 0 | 1 | exp1: Participants + Outcome; exp2: Participants + Outcome |
| Taylor & Shaw (2002) The effects of outcome imagery on golf-putting performance | 1 | 0 | 1 | 1 | 1 | 1 | 0 | 1 | Participants |
| Tenenbaum (1999) The effect of goal difficulty and goal orientation on running performance in young female athletes | 1 | 0 | 1 | 1 | 1 | 1 | 0 | 1 | Participants |
| Theodorakis et al. (1998) Combined effects of goal setting and performance feedback on performance and physiological response on a maximum effort task | 1 | 0 | 1 | 1 | 1 | 1 | 0 | 1 | Participants |
| Theodorakis et al. (2000) The effects of motivational versus instructional self-talk on improving motor performance | 1 | exp1: 0; exp2: 0; exp3: 0; exp4: 0 | exp1: 1; exp2: 1; exp3:1; exp4: 1 | exp1: 1; exp2: 1; exp3: 1; exp4: 1 | exp1: 1; exp2: 1; exp3: 1; exp4: 1 | exp1: 1; exp2: 1; exp3: 0 exp4: 0 | exp1: 0; exp2: 0; exp3: 0; exp4: 0 | exp1: 1; exp2: 1; exp3: 1; exp4: 1 | exp1: Participants; exp2: Participants; exp3: Participants + Outcome; exp4: Participants + Outcome |
| Theodorakis et al. (2001) Self-talk in a basketball-shooting task | 1 | 0 | 1 | 1 | 1 | 1 | 0 | 0 | Participants |
| Thompson et al. (2011) One year follow-up of Mindful Sport Performance Enhancement (MSPE) with archers, golfers, and runners | 1 |  |  |  |  |  |  |  | Follow-up study to other excluded studies: De Petrillo et al. (2009) & Kaufman et al. (2009) |
| Thompson et al. (2021) Pleasant Emotions Widen Thought-Action Repertoires, Develop Long-Term Resources, and Improve Reaction Time Performance: A Multistudy Examination of the Broaden-and-Build Theory Among Athletes | 1 | exp2: 0 | exp1: 0; exp2: 1 | exp2: 1 | exp2: 1 | exp2: 0 | exp2: 0 | exp2: 1 | exp1: Intervention exp2: Participants + Outcome |
| Thow et al. (2012) Comparison of modes of feedback on glide performance in swimming | 1 |  | 0 |  |  |  |  |  | Intervention |
| Tibor Latinjak et al. (2011) Comparing the effects of different self-instructions on thought content and tennis performance | 1 |  | 0 |  |  |  |  |  | Intervention |
| Todorov et al. (1997) Augmented feedback presented in a virtual environment accelerates learning of a difficult motor task | 1 |  | 0 |  |  |  |  |  | Intervention |
| Tony et al. (1978) The content and effect of “Psyching-Up” strategies in weight lifters | 1 | 0 | 1 | 1 | 1 | 1 | 0 | 1 | Participants |
| Tsetseli et al. (2016) The effect of internal and external focus of attention on game performance in tennis | 1 |  | 0 |  |  |  |  |  | Intervention |
| Tu & Rothstein (1979) Improvement of jogging performance through application of personality specific motivational techniques | 1 | 0 | 1 | 1 | 1 | 1 | 0 | 0 | Participants |
| Tuomilehto et al. (2017) Sleep of professional athletes: Underexploited potential to improve health and performance | 1 | 1 | 1 | 1 | 1 | 0 | 0 | 1 | Outcome |
| Tynes & McFatter (1987) The efficacy of "psyching" strategies on a weight-lifting task | 1 | 0 | 1 | 1 | 1 | 1 | 0 | 0 | Participants |
| Tzetzis et al. (2008) The effect of different corrective feedback methods on the outcome and self confidence of young athletes | 1 |  | 0 |  |  |  |  |  | Intervention |
| Ugurlu et al. (2021) Examination of the Effects of Autogenic Training on the Reaction Time Performance of the National Badminton Athletes | 1 | 1 | 1 | 1 | 1 | 0 | 0 | 1 | Outcome |
| Van Gyn et al. (1990) Imagery as a method of enhancing transfer from training to performance | 1 | 0 | 1 | 1 | 1 | 1 | 0 | 0 | Participants |
| Van Raalte et al. (2018) Self-Talk in a SCUBA Diving Contex. | 1 | 0 | 1 | 0 | 1 | 0 | 0 | 1 | Participants + Not performance enhancement + Outcome |
| Veskovic et al. (2019) Effects of a Psychological Skill Training Program on Anxiety Levels In Top Karate Athletes | 1 | 1 | 1 | 1 | 1 | 0 | 0 | 1 | Outcome |
| Vidic & Cherup (2022) Take me into the ball game: An examination of a brief psychological skills training and mindfulness-based intervention with baseball players | 1 | 1 | 1 | 1 | 1 | 0 | 0 | 1 | Outcome |
| Vidic et al. (2018) Mindfulness Meditation Intervention with Male Collegiate Soccer Players: Effect on Stress and Various Aspects of Life | 1 | 1 | 1 | 1 | 1 | 0 | 0 | 1 | Outcome |
| Vidic et al. (2017) Mindfulness Intervention With a US Women's NCAA Division I Basketball Team: Impact on Stress, Athletic Coping Skills and Perceptions of Intervention | 1 | 1 | 1 | 1 | 1 | 0 | 0 | 1 | Outcome |
| Vidic (2021) Sharpening the Mental Edge in Ice-Hockey: Impact of a Season-Long Psychological Skills Training and Mindfulness Intervention on Athletic Coping Skills, Resilience, Stress and Mindfulness | 1 | 1 | 1 | 1 | 1 | 0 | 0 | 1 | Outcome |
| Vitali et al. (2019) Action Monitoring Through External or Internal Focus of Attention Does Not Impair Endurance Performance | 1 | 0 | 1 | 1 | 1 | 1 | 0 | 1 | Participants |
| Vodi-íar et al. (2012) Effectiveness of athletes' pre-competition mental preparation. | 1 | 1 | 1 | 1 | 1 | 0 | 0 | 1 | Outcome |
| Voight (2005) Integrating Mental-Skill Training into Everyday Coaching |  |  | 0 |  |  |  |  |  | Intervention |
| Wakefield & Smith (2009) Impact of differing frequencies of PETTLEP imagery on netball shooting performance | 1 | 0 | 1 | 1 | 1 | 1 | 0 | 1 | Participants |
| Waldron et al. (2015) Augmenting performance feedback does not affect 4 km cycling time-trials in the heat | 1 | 0 | 1 | 1 | 1 | 1 | 0 | 1 | Participants |
| Wali-Menzli et al. (2019) Role of the Mental Representation In Enhancing Motor Learning And Performing Gymnastic Element | 1 | 0 | 1 | 1 | 1 | 1 | 0 | 0 | Participants |
| Wallace et al. (2017) Effects of Motivational Self-Talk on Endurance and Cognitive Performance in the Heat | 1 | 0 | 1 | 1 | 1 | 1 | 0 | 1 | Participants |
| Wallace et al. (2017) Effects of Motivational Self-Talk on Endurance and Cognitive Performance in the Heat | 1 | 0 | 1 | 1 | 1 | 1 | 0 | 1 | Participants |
| Wang et al. (2017) Effects of applying virtual reality to adventure athletic education on students' self-efficacy and team cohesiveness | 1 |  | 0 |  |  |  |  |  | Intervention |
| Wang et al. (2021) On Mindfulness Training for Promoting Mental Toughness of Female College Students in Endurance Exercise | 1 | 0 | 1 | 1 | 1 | 0 | 0 | 1 | Participants + Outcome |
| Weber (2021) Pychological Performance During Running Exercise: Effect of Olfactory Imagery On Distance | 1 | 0 | 1 | 1 | 1 | 1 | 0 | 0 | Participants |
| Weinberg (1986) Relationship between self-efficacy and cognitive strategies in enhancing endurance performance. | 1 | 0 | 1 | 1 | 1 | 1 | 0 | 1 | Participants |
| Weinberg et al. (1981) Effects of Visuo-motor Behavior Rehearsal, Relaxation, and Imagery on Karate Performance | 1 | 0 | 1 | 1 | 1 | 0 | 1 | 1 | Participants |
| Weinberg et al. (1983) Mental preparation strategies and performance: is a combination of techniques better than a single technique? | 1 | 0 | 1 | 1 | 1 | 1 | 0 | 0 | Participants |
| Weinberg et al. (1984) Effect of association, dissociation and positive self-talk strategies on endurance performance | 1 | exp1: 0 exp2: 0 | 1 | 1 | 1 | exp1: 1 exp2: 1 | 0 | 1 | exp1: Participants  exp2: Participants |
| Weinberg et al. (1985) The effects of specific vs nonspecific mental preparation strategies on strength and endurance performance | 1 | 0 | 1 | 1 | 1 | 1 | 0 | 1 | Participants |
| Weinberg et al. (2019) Writing down goals: Does it actually improve performance? | 1 | 0 | 1 | 1 | 1 | 1 | 0 | 1 | Participants |
| Weinberg et al. (1980) Influence of cognitive strategies on tennis serves of players of high and low ability | 1 | 0 | 1 | 1 | 1 | 1 | 0 | 1 | Participants |
| Weston et al. (2011) The impact of a performance profiling intervention on athletes' intrinsic motivation | 1 | 1 | 1 | 1 | 1 | 0 | 0 | 1 | Outcome |
| Whelan et al. (1990) Arousal interventions for athletic performance: Influence of mental preparation and competitive experience | 1 | 0 | 1 | 1 | 1 | 0 | 0 | 1 | Participants + Outcome |
| White & Hardy (1998) An In-Depth Analysis of the Uses of Imagery by High-Level Slalom Canoeists and Artistic Gymnasts | 1 |  | 0 |  |  |  |  |  | Intervention |
| Wikman et al. (2014) Effects of goal setting on fear of failure in young elite athletes | 1 | 0 | 1 | 1 | 1 | 0 | 0 | 1 | Participants + Outcome |
| Williams et al. (2010) The use of imagery to manipulate challenge and threat appraisal States in athletes | 1 | 0 | 1 | 0 | 1 | 0 | 0 | 1 | Participants + Not performance enhancement + Outcome |
| Williamson (1982) The Effects of two Stress Management Training Programs on Cardiorespiratory Efficiency | 1 |  | 0 |  |  |  |  |  | Intervention |
| Winfrey & Weeks (1993) Effects of self-modeling on self-efficacy and balance beam performance | 1 |  | 0 |  |  |  |  |  | Intervention |
| Winkelman et al. (2017) Experience level influences the effect of attentional focus on sprint performance | 1 |  | 0 |  |  |  |  |  | Intervention |
| Wolch et al. (2021) The effects of a brief mindfulness intervention on basketball free-throw shooting performance under pressure | 1 | 0 | 1 | 1 | 1 | 1 | 0 | 1 | Participants |
| Wolframm & Micklewright (2011) The effect of a mental training program on state anxiety and competitive dressage performance | 1 | 0 | 1 | 1 | 1 | 1 | 0 | 1 | Participants |
| Wood & Wilson (2012) Quiet-eye training, perceived control and performing under pressure | 1 |  | 0 |  |  |  |  |  | Intervention |
| Wood et al. (2018) Examining the effects of rational emotive behavior therapy on performance outcomes in elite paralympic athletes | 1 | 1 | 1 | 1 | 0 | 1 | 0 | 1 | Design |
| Wood et al. (2018) Exploring the Effects of a Single Rational Emotive Behavior Therapy Workshop in Elite Blind Soccer Players | 1 | 0 | 1 | 1 | 1 | 0 | 1 | 1 | Participants |
| Woodman et al. (2010) Self-confidence and performance: A little self-doubt helps | 1 | 0 | 1 | 1 | 1 | 1 | 0 | 1 | Participants |
| Woolfolk et al. (1985) Effects of mental rehearsal of task motor activity and mental depiction of task outcome on motor skill performance | 1 | 0 | 1 | 1 | 1 | 1 | 0 | 1 | Participants |
| Woolfolk et al. (1985) The effects of positive and negative imagery on motor skill performance | 1 | 0 | 1 | 1 | 1 | 1 | 0 | 0 | Participants |
| Wright & O’Halloran (2013) Perceived success, auditory feedback, and mental imagery: what best predicts improved efficacy and motor performance? | 1 | 0 | 1 | 1 | 1 | 1 | 0 | 1 | Participants |
| Wright & Smith (2009) The effect of PETTLEP imagery on strength performance | 1 | 0 | 1 | 1 | 1 | 1 | 0 | 1 | Participants |
| Wright et al. (2015) Action observation and imagery training improve the ease with which athletes can generate imagery | 1 | 0 | 1 | 1 | 1 | 0 | 0 | 1 | Participants + Outcome |
| Wright et al. (2016) Enhancing Self-Efficacy and Performance: An Experimental Comparison of Psychological Techniques | 1 | 0 | 1 | 1 | 1 | 1 | 0 | 1 | Participants |
| Wrisberg & Anshel (1997) The use of positively-worded performance reminderns to reduce warm-up decrement in the field hockey penalty shot | 1 | 0 | 1 | 1 | 1 | 1 | 1 | 0 | Participants |
| Wulf (2008) Attentional Focus Effects in Balance Acrobats | 1 | 0 | 1 | 1 | 1 | 1 | 0 | 0 | Participants |
| Wulf & Dufek (2009) Increased jump height with external focus due to enhanced lower extremity joint kinetics | 1 | 0 | 1 | 1 | 1 | 1 | 0 | 1 | Participants |
| Wulf et al. (2002) Enhancing the Learning of Sport Skills Through External-Focus Feedback | 1 |  | exp1: 0  exp2: 0 |  |  |  |  |  | exp1: Intervention exp2: Intervention |
| Yamada et al. (2021) The Effects of Using Imagery to Elicit an External Focus of Attention | 1 | 0 | 1 | 1 | 1 | 1 | 0 | 0 | Participants |
| Yau et al. (2021) The Use of Mindfulness Acceptance Commitment (MAC) Approach For Malaysian Elite Triathletes | 1 | 0 | 1 | 1 | 0 | 0 | 0 | 1 | Participants + Design + Outcome |
| Yeemin et al. (2020) The effects of post activation potentiation warm-up and pre-shot routine programs on driving performance in amateur golfers | 1 |  | 0 |  |  |  |  |  | Intervention |
| Zarghami et al. (2012) External focus of attention enhances discus throwing performance | 1 |  | 0 |  |  |  |  |  | Intervention |
| Zestcott et al. (2016) He Dies, He Scores: Evidence That Reminders of Death Motivate Improved Performance in Basketball | 1 | exp1: 0; exp2: 0 | exp: 1; exp2: 1 | exp: 1; exp: 1 | exp1: 1; exp2: 1 | exp1: 1; exp2: 1 | exp1: 0; exp2: 0 | exp1: 1; exp2: 1 | exp1: Participants  exp2: Participants |
| Zhou et al. (2020) The effects of visual training on sports skill in volleyball players | 1 |  | exp1: 0; exp2: 0; exp3: 0; exp4: 0 |  |  |  |  |  | exp1: Intervention exp2: Intervention exp3: Intervention exp4: Intervention |
| Zhu et al. (2020) Acute Effects of Brief Mindfulness Intervention Coupled with Carbohydrate Ingestion to Re-Energize Soccer Players: A Randomized Crossover Trial | 1 | 0 | 1 | 1 | 1 | 0 | 0 | 1 | Participants + Outcome |

*Note.* 1 = inclusion according to criteria; 0 = exclusion according to criteria; ^a^ Cardinal criterion = no further eligibility criteria were assessed if excluded
