# Supplementary material for: Effects of Psychological Interventions to Enhance Athletic Performance: A Systematic Review and Meta-Analysis
Source: Sports Med. 2023 Oct 9;54(2):347–73. doi: 10.1007/s40279-023-01931-z (PMC10933186; doi:10.1007/s40279-023-01931-z)
Supplement: Supplementary file 3 — Supplementary file3 (DOCX 41 KB) [file 40279_2023_1931_MOESM3_ESM.docx]

**Supplementary information. Online Resource 3.**

*Article:* Effects of psychological interventions to enhance athletic performance: A systematic review and meta-analysis

*Journal:* Sports Medicine

*Authors:* Gustaf Reinebo, Sven Alfonsson, Markus Jansson-Fröjmark, Alexander Rozental, Tobias Lundgren

*Corresponding author:* Gustaf Reinebo, email [gustaf.reinebo@ki.se](mailto:gustaf.reinebo@ki.se) , Centre for Psychiatry Research, Department of Clinical Neuroscience, Karolinska Institutet, & Stockholm Health Care Services, Region Stockholm, Norra stationsgatan 69, SE-113 64, Stockholm, Sweden

**Risk of bias assessment of systematic review studies**

| Study | Item1 | Item2 | Item3 | Item4a | Item4b | Item5a | Item5b | Item6 | Item7a | Item7b | Item8 | Item9 | Risk of bias score |
| --- | --- | --- | --- | --- | --- | --- | --- | --- | --- | --- | --- | --- | --- |
| Abdoli et al. [1] | 0 | 0 | -1 | 0 | 0 | 0 | 0 | 0 | -1 | -1 | -1 | 0 | -4 |
| Ahmadzadeh et al. [2] | 0 | -1 | -1 | 0 | -1 | 0 | 0 | 0 | -1 | -1 | -1 | 0 | -6 |
| Anderson et al. [3] | -2 | -1 | -1 | -1 | -1 | 0 | -1 | 0 | -1 | -1 | -1 | -1 | -11 |
| Anshel [4] | -2 | -1 | -1 | -1 | 0 | 0 | 0 | -1 | -1 | -1 | -1 | -1 | -10 |
| Ashford and Jackson [5] Experiment 1 | 0 | -1 | -1 | 0 | -1 | 0 | 0 | 0 | -1 | -1 | -1 | 0 | -6 |
| Ashford and Jackson [5] Experiment 2 | 0 | -1 | -1 | 0 | -1 | 0 | 0 | 0 | -1 | -1 | -1 | 0 | -6 |
| Bakker and Kayser [6] | 0 | -1 | -1 | 0 | 0 | 0 | -1 | -1 | -1 | 0 | -1 | -1 | -7 |
| Barker et al. [7] | 0 | 0 | -1 | 0 | -1 | 0 | 0 | -1 | 0 | 0 | -1 | 0 | -4 |
| Blair et al. [8] | 0 | 0 | -1 | 0 | 0 | 0 | 0 | -1 | -1 | -1 | -1 | -1 | -6 |
| Boutcher and Crews [9] | 0 | 0 | -1 | 0 | 0 | 0 | 0 | -1 | -1 | -1 | -1 | -1 | -6 |
| Burton [10] | 0 | -1 | -1 | 0 | -1 | 0 | 0 | -1 | -1 | 0 | -1 | -1 | -7 |
| Caudill et al. [11] Experiment 1 | 0 | -1 | -1 | 0 | 0 | 0 | 0 | 0 | 0 | 0 | -1 | -1 | -4 |
| Caudill et al. [11] Experiment 2 | 0 | -1 | -1 | 0 | 0 | 0 | 0 | 0 | 0 | 0 | -1 | -1 | -4 |
| Connolly and Janelle [12] Experiment 1 | 0 | 0 | -1 | 0 | 0 | 0 | 0 | 0 | -1 | 0 | -1 | -1 | -4 |
| Connolly and Janelle [12] Experiment 2 | 0 | -1 | -1 | 0 | 0 | 0 | 0 | 0 | -1 | 0 | -1 | -1 | -5 |
| Couvillion and Fairbrother [13] | 0 | -1 | -1 | 0 | 0 | 0 | 0 | -1 | 0 | 0 | -1 | -1 | -5 |
| Dahm and Rieger [14] | 0 | -1 | -1 | 0 | 0 | 0 | 0 | 0 | -1 | 0 | -1 | 0 | -4 |
| Daneshfar et al. [15] | 0 | 0 | -1 | 0 | 0 | 0 | 0 | 0 | -1 | -1 | -1 | 0 | -4 |
| Daw and Burton [16] | 0 | -1 | -1 | 0 | -1 | 0 | 0 | -1 | -1 | -1 | 0 | -1 | -7 |
| de Matos et al. [17] | 0 | 0 | -1 | 0 | 0 | 0 | 0 | 0 | -1 | 0 | 0 | 0 | -2 |
| Donohue et al. [18] | 0 | 0 | -1 | 0 | 0 | 0 | 0 | 0 | 0 | 0 | -1 | -1 | -3 |
| Gray and Fernandez [19] | -2 | -1 | -1 | 0 | -1 | 0 | 0 | 0 | 0 | 0 | -1 | -1 | -7 |
| Gross et al. [20] | 0 | 0 | -1 | 0 | 0 | 0 | -1 | 0 | -1 | 0 | -1 | 0 | -4 |
| Gröpel [21] | -2 | -1 | -1 | 0 | -1 | 0 | 0 | 0 | 0 | 0 | -1 | 0 | -6 |
| Guillot et al. [22] | 0 | 0 | -1 | -1 | 0 | 0 | 0 | -1 | -1 | -1 | -1 | 0 | -6 |
| Guillot et al. [23] | 0 | -1 | -1 | 0 | 0 | 0 | -1 | -1 | -1 | -1 | -1 | -1 | -8 |
| Hall and Erffmeyer [24] | 0 | 0 | -1 | 0 | -1 | 0 | 0 | 0 | -1 | -1 | -1 | -1 | -6 |
| Halperin et al. [25] | 0 | 0 | -1 | 0 | 0 | 0 | 0 | -1 | -1 | -1 | -1 | 0 | -5 |
| Hammoudi-Nassib et al. [26] | 0 | 0 | -1 | 0 | -1 | 0 | 0 | 0 | -1 | -1 | 0 | 0 | -4 |
| Holm et al. [27] | 0 | 0 | -1 | 0 | 0 | 0 | 0 | -1 | -1 | -1 | -1 | -1 | -6 |
| Horton and Shelton [28] | -2 | -1 | -1 | -1 | -1 | 0 | 0 | 0 | -1 | -1 | -1 | -1 | -10 |
| Hut et al. [29] | 0 | 0 | -1 | 0 | -1 | 0 | 0 | -1 | -1 | 0 | -1 | 0 | -5 |
| Ille et al. [30] | 0 | -1 | -1 | 0 | 0 | 0 | 0 | -1 | -1 | -1 | -1 | 0 | -6 |
| Kacperski and Hall [31] Experiment 1 | 0 | -1 | -1 | 0 | -1 | 0 | 0 | 0 | 0 | 0 | -1 | 0 | -4 |
| Kacperski and Hall [31] Experiment 2 | 0 | -1 | -1 | 0 | 0 | 0 | 0 | 0 | 0 | 0 | -1 | 0 | -3 |
| Kanthack et al. [32] Experiment 1 | 0 | -1 | -1 | 0 | 0 | 0 | 0 | -1 | -1 | -1 | -1 | -1 | -7 |
| Kanthack et al. [32] Experiment 2 | 0 | -1 | -1 | 0 | 0 | 0 | 0 | -1 | -1 | -1 | -1 | -1 | -7 |
| Klatt and Noël [33] Experiment 1 | 0 | -1 | -1 | 0 | 0 | 0 | 0 | 0 | 0 | 0 | -1 | 0 | -3 |
| Klatt and Noël [33] Experiment 2 | 0 | -1 | -1 | 0 | 0 | 0 | 0 | 0 | 0 | 0 | -1 | 0 | -3 |
| Kutzner et al. [34] | 0 | 0 | -1 | 0 | -1 | 0 | 0 | 0 | 0 | 0 | -1 | -1 | -4 |
| Lamirand and Rainey [35] | 0 | -1 | -1 | 0 | -1 | 0 | 0 | 0 | -1 | -1 | -1 | -1 | -7 |
| Liang et al. [36] | 0 | -1 | -1 | 0 | -1 | 0 | 0 | 0 | 0 | 0 | -1 | 0 | -4 |
| Madden and McGown [37] | 0 | 0 | -1 | 0 | -1 | 0 | 0 | -1 | -1 | -1 | -1 | -1 | -7 |
| Maynard, Hemmings and Warwick-Evans [38] | 0 | -1 | -1 | 0 | -1 | -1 | -1 | 0 | -1 | 0 | -1 | -1 | -8 |
| Maynard, Smith and Warwick-Evans [39] | 0 | -1 | -1 | 0 | -1 | 0 | -1 | 0 | -1 | -1 | -1 | 0 | -7 |
| McAleney et al. [40] | 0 | 0 | -1 | 0 | -1 | 0 | 0 | -1 | -1 | -1 | -1 | -1 | -7 |
| Milley and Ouellette [41] | 0 | 0 | -1 | 0 | 0 | 0 | 0 | -1 | 0 | 0 | -1 | 0 | -3 |
| Minkler et al. [42] | -2 | -1 | -1 | 0 | 0 | 0 | 0 | 0 | 0 | 0 | -1 | 0 | -5 |
| Mumford and Hall [43] | 0 | 0 | -1 | -1 | -1 | 0 | -1 | 0 | -1 | -1 | -1 | -1 | -8 |
| Neumann and Hohnke [44] | 0 | 0 | -1 | 0 | -1 | 0 | 0 | 0 | -1 | -1 | -1 | 0 | -5 |
| Noel [45] | 0 | 0 | -1 | 0 | -1 | 0 | -1 | -1 | -1 | -1 | -1 | -1 | -8 |
| Padgett and Hill [46] Experiment 2 | 0 | 0 | -1 | 0 | 0 | 0 | 0 | 0 | 0 | 0 | -1 | -1 | -3 |
| Palao et al. [47] | -2 | -1 | -1 | 0 | -1 | 0 | 0 | -1 | -1 | -1 | -1 | -1 | -10 |
| Papaioannou et al. [48] | 0 | -1 | -1 | 0 | -1 | 0 | 0 | 0 | -1 | -1 | -1 | -1 | -7 |
| Plessner et al. [49] | 0 | 0 | -1 | 0 | -1 | 0 | 0 | 0 | -1 | 0 | -1 | 0 | -4 |
| Reeves et al. [50] | 0 | 0 | -1 | 0 | -1 | 0 | 0 | -1 | -1 | -1 | -1 | 0 | -6 |
| Savoy and Beitel [51] | -2 | -1 | -1 | 0 | 0 | 0 | 0 | 0 | -1 | -1 | -1 | -1 | -8 |
| Schorer et al. [52] | 0 | 0 | -1 | 0 | 0 | 0 | 0 | 0 | -1 | 0 | 0 | -1 | -3 |
| Schreiber [53] | 0 | 0 | -1 | -1 | 0 | 0 | 0 | 0 | -1 | 0 | -1 | -1 | -5 |
| Scott et al. [54] | -2 | -1 | -1 | 0 | 0 | 0 | -1 | -1 | 0 | 0 | -1 | -1 | -8 |
| Shahi et al. [55] | 0 | 0 | -1 | -1 | -1 | 0 | 0 | 0 | -1 | -1 | -1 | -1 | -7 |
| Shoenfelt [56] | 0 | -1 | -1 | -1 | -1 | 0 | 0 | 0 | -1 | -1 | -1 | -1 | -8 |
| Shoenfelt and Griffith [57] | -2 | -1 | -1 | 0 | -1 | 0 | -1 | -1 | 0 | 0 | -1 | -1 | -9 |
| Simões et al. [58] | -2 | -1 | -1 | 0 | -1 | 0 | 0 | 0 | 0 | 0 | -1 | -1 | -7 |
| Smith and Holmes [59] | 0 | 0 | -1 | 0 | 0 | 0 | 0 | 0 | -1 | -1 | -1 | 0 | -4 |
| Smith et al. [60] | 0 | 0 | -1 | 0 | 0 | 0 | 0 | 0 | -1 | -1 | -1 | 0 | -4 |
| Smith et al. [61] | 0 | 0 | -1 | 0 | 0 | 0 | 0 | 0 | 0 | 0 | -1 | 0 | -2 |
| Spindler et al. [62] | 0 | 0 | -1 | 0 | 0 | 0 | 0 | -1 | 0 | 0 | -1 | 0 | -3 |
| Stamou et al. [63] | 0 | -1 | -1 | 0 | 0 | 0 | 0 | 0 | -1 | -1 | -1 | -1 | -6 |
| Takeuchi et al. [64] Experiment 2 | 0 | -1 | -1 | 0 | -1 | 0 | 0 | 0 | 0 | 0 | -1 | 0 | -4 |
| Vast et al. [65] | 0 | -1 | -1 | 0 | 0 | 0 | 0 | 0 | -1 | -1 | -1 | 0 | -5 |
| Velentzas et al. [66] | 0 | -1 | -1 | 0 | -1 | 0 | 0 | -1 | -1 | -1 | -1 | 0 | -7 |
| Wagaman et al. [67] | 0 | 0 | -1 | 0 | -1 | 0 | 0 | -1 | -1 | -1 | -1 | -1 | -7 |
| Weinberg et al. [68] | 0 | -1 | -1 | 0 | -1 | 0 | 0 | 0 | -1 | -1 | -1 | 0 | -6 |
| Weinberg et al. [69] | 0 | -1 | -1 | 0 | 0 | 0 | 0 | 0 | -1 | -1 | -1 | -1 | -6 |
| Wergin et al. [70] | 0 | 0 | -1 | 0 | -1 | 0 | 0 | -1 | 0 | 0 | -1 | 0 | -4 |
| Westlund Stewart and Hall [71] | -2 | -1 | -1 | 0 | 0 | 0 | 0 | -1 | -1 | -1 | -1 | 0 | -8 |
| Whdan [72] | -2 | -1 | -1 | -1 | -1 | 0 | 0 | -1 | -1 | -1 | -1 | -1 | -11 |
| Winter and Collins [73] | 0 | -1 | -1 | 0 | 0 | 0 | 0 | 0 | -1 | -1 | -1 | 0 | -5 |
| Wojcikiewicz and Orlick [74] | 0 | 0 | -1 | 0 | -1 | 0 | 0 | -1 | -1 | 0 | -1 | -1 | -6 |
| Wolanin and Schwanhausser [75] | 0 | -1 | -1 | 0 | 0 | 0 | -1 | -1 | -1 | -1 | -1 | -1 | -8 |
| Wollman et al. [76] Experiment 1 | 0 | 0 | -1 | -1 | -1 | 0 | 0 | -1 | -1 | -1 | -1 | -1 | -8 |
| Wollman et al. [76] Experiment 2 | 0 | -1 | -1 | -1 | -1 | 0 | 0 | 0 | -1 | -1 | -1 | -1 | -8 |
| Woolsey et al. [77] | -2 | -1 | -1 | 0 | -1 | 0 | 0 | -1 | 0 | 0 | -1 | -1 | -8 |
| Wu et al. [78] | -2 | -1 | -1 | 0 | -1 | 0 | 0 | 0 | 0 | 0 | -1 | 0 | -6 |
| Zervas and Kakkos [79] | 0 | -1 | -1 | 0 | 0 | 0 | 0 | 0 | 0 | 0 | -1 | -1 | -4 |

*Note.* Item1: Is there a control group? (yes = 0, no = -2); Item2: Is randomization conducted? (yes = 0, no = -1); Item3: Study protocol registered a priori? (yes = 0, no = -1); Item4a: Is the intervention protocol and procedure clearly described? (yes = 0, no = -1); Item4b: Is intervention adherence investigated or a manipulation check conducted? (yes = 0, no = -1); Item5a: An adequate primary outcome measure with acceptable psychometric qualities? (yes = 0, no = -1, if other measure than a psychometric instrument is adequately used as primary outcome = 0); Item5b: Blinded outcome assessors? (yes = 0, no = -1, if not applicable to design = 0); Item6: Clearly defined pre-specified primary outcome OR adjusted p values for multiple testing? (yes = 0, no = -1); Item7a: Intention-to-treat analysis? (yes = 0, no = -1); Item7b: Reporting drop-outs? (yes = 0, no = -1); Item8: Conducted interim analysis to detect deterioration OR investigated side-effects? (yes = 0, no = -1); Item9: Are effect sizes calculated OR a measure of clinical significance used for the primary outcome? (yes = 0, no = -1); Green = Yes/Criterion fulfilled; Red = No/Criterion not fulfilled

**References**

1. Abdoli B, Hardy J, Riyahi JF, Farsi A. A closer look at how self-talk influences skilled basketball performance. The Sport Psychologist. 2018;32(1):9-15. <https://doi.org/10.1123/tsp.2016-0162>

2. Ahmadzadeh S, Badami R, Aghaei A. The Effectiveness of Neuro-Linguistic Programming (NLP) on Shooters’ Mental Skills and Shooting Performance. Iran J Psychiatry Behav Sci. 2019;13(3):e84124. <https://doi.org/10.5812/ijpbs.84124>

3. Anderson DC, Crowell CR, Doman M, Howard GS. Performance posting, goal setting, and activity-contingent praise as applied to a university hockey team. Journal of Applied Psychology. 1988;73(1):87–95. <https://doi.org/10.1037/0021-9010.73.1.87>

4. Anshel MH. Toward validation of a model for coping with acute stress in sport. Int J Sport Psychol. 1990;21(1):58-83.

5. Ashford KJ, Jackson RC. Priming as a means of preventing skill failure under pressure. Journal of Sport and Exercise Psychology. 2010;32(4):518-36. <https://doi.org/10.1123/jsep.32.4.518>

6. Bakker FC, Kayser CS. Effect of a self-help mental training programme. Int J Sport Psychol. 1994;25(2):158-75.

7. Barker J, Jones M, Greenlees I. Assessing the immediate and maintained effects of hypnosis on self-efficacy and soccer wall-volley performance. Journal of Sport and Exercise Psychology. 2010;32(2):243–52. <https://doi.org/10.1123/jsep.32.2.243>

8. Blair A, Hall C, Leyshon G. Imagery effects on the performance of skilled and novice soccer players. Journal of Sports Sciences. 1993;11(2):95-101. <https://doi.org/10.1080/02640419308729971>

9. Boutcher SH, Crews DJ. The effect of a preshot attentional routine on a well-learned skill. Int J Sport Psychol. 1987;18(1):30-9.

10. Burton D. Winning isn’t everything: Examining the impact of performance goals on collegiate swimmers’ cognitions and performance. The Sport Psychologist. 1989;3(2):105–32. <https://doi.org/10.1123/tsp.3.2.105>

11. Caudill D, Weinberg R, Jackson A. Psyching-up and track athletes: A preliminary investigation. Journal of Sport Psychology. 1983;5(2):231-5. <https://doi.org/10.1123/jsp.5.2.231>

12. Connolly CT, Janelle CM. Attentional strategies in rowing: Performance, perceived exertion, and gender considerations. J Appl Sport Psychol. 2003;15(3):195-212. <https://doi.org/10.1080/10413200305387>

13. Couvillion KF, Fairbrother JT. Expert and novice performers respond differently to attentional focus cues for speed jump roping. Frontiers in Psychology. 2018;9:2370. <https://doi.org/10.3389/fpsyg.2018.02370>

14. Dahm SF, Rieger M. Is imagery better than reality? Performance in imagined dart throwing. Human Movement Science. 2019;66:38-52. <https://doi.org/10.1016/j.humov.2019.03.005>

15. Daneshfar A, Petersen CJ, Gahreman DE. The effect of 4 weeks motor imagery training on simulated bmx race performance. Int J Sport Exerc Psychol. 2022;20(2):644–60. <https://doi.org/10.1080/1612197X.2020.1869801>

16. Daw J, Burton D. Evaluation of a comprehensive psychological skills training program for collegiate tennis players. The Sport Psychologist. 1994;8(1):37-57. <https://doi.org/10.1123/tsp.8.1.37>

17. de Matos LF, Bertollo M, Stefanello JMF, Pires FO, da Silva CK, Nakamura FY, et al. Motivational self-talk improves time-trial swimming endurance performance in amateur triathletes. Int J Sport Exerc Psychol. 2021;19(3):446–59. <https://doi.org/10.1080/1612197X.2020.1717576>

18. Donohue B, Barnhart R, Covassin T, Carpin K, Korb E. The development and initial evaluation of two promising mental preparatory methods in a sample of female cross country runners. Journal of Sport Behavior. 2001;24(1):19-30.

19. Gray SW, Fernandez SJ. Effects of Visuo-Motor Behavior Rehearsal with videotaped modeling on basketball shooting performance. Psychology: A Journal of Human Behavior. 1989;26(4):41-7.

20. Gross M, Moore ZE, Gardner FL, Wolanin AT, Pess R, Marks DR. An empirical examination comparing the Mindfulness-Acceptance-Commitment approach and Psychological Skills Training for the mental health and sport performance of female student athletes. Int J Sport Exerc Psychol. 2018;16(4):431-51. <https://doi.org/10.1080/1612197x.2016.1250802>

21. Gröpel P. Self-focused attention and motor skill failure: The moderating role of action orientation. Sport, Exercise, and Performance Psychology. 2016;5(3):206-17. <https://doi.org/10.1037/spy0000059>

22. Guillot A, Moschberger K, Collet C. Coupling movement with imagery as a new perspective for motor imagery practice. Behavioral and Brain Functions. 2013;9, 8. <https://doi.org/10.1186/1744-9081-9-8>

23. Guillot A, Nadrowska E, Collet C. Using motor imagery to learn tactical movements in basketball. Journal of Sport Behavior. 2009;32(2):189-206.

24. Hall EG, Erffmeyer ES. The effect of Visuo-Motor Behavior Rehearsal with videotaped modeling on free throw accuracy of intercollegiate female basketball players. Journal of Sport Psychology. 1983;5(3):343-6. <https://doi.org/10.1123/jsp.5.3.343>

25. Halperin I, Chapman DW, Thompson KG, Abbiss C. False-performance feedback does not affect punching forces and pacing of elite boxers. Journal of Sport Sciences. 2019;37(1):59-66. <https://doi.org/10.1080/02640414.2018.1482526>

26. Hammoudi-Nassib S, Nassib S, Chtara M, Briki W, Chaouachi A, Tod D, et al. Effects of psyching-up on sprint performance. Journal of Strength & Conditioning Research. 2017;31(8):2066-74. <https://doi.org/10.1519/JSC.0000000000000373>

27. Holm JE, Beckwith BE, Ehde DM, Tinius TP. Cognitive-behavioral interventions for improving performance in competitive athletes: A controlled treatment outcome study. Int J Sport Psychol. 1996;27(4):463-75.

28. Horton AM, Jr., Shelton JK. The rational wrestler—A pilot study. Percept Mot Skills. 1978;46:882. <https://doi.org/10.2466/pms.1978.46.3.882>

29. Hut M, Minkler TO, Glass CR, Weppner CH, Thomas HM, Flannery CB. A randomized controlled study of mindful sport performance enhancement and psychological skills training with collegiate track and field athletes. J Appl Sport Psychol. 2021:1-23. <https://doi.org/10.1080/10413200.2021.1989521>

30. Ille A, Selin I, Do M-C, Thon B. Attentional focus effects on sprint start performance as a function of skill level. Journal of Sport Sciences. 2013;31(15):1705-12. <https://doi.org/10.1080/02640414.2013.797097>

31. Kacperski C, Hall C. Do construal levels affect athletes' imagery and performance outcomes? It depends on the task! J Appl Sport Psychol. 2017;29(2):181-98. <https://doi.org/10.1080/10413200.2016.1220992>

32. Kanthack TFD, Guillot A, Altimari LR, Nunez Nagy S, Collet C, Di Rienzo F. Selective efficacy of static and dynamic imagery in different states of physical fatigue. PLoS One. 2016;11(3):e0149654. <https://doi.org/10.1371/journal.pone.0149654>

33. Klatt S, Noël B. Regulatory focus in sport revisited: Does the exact wording of instructions really matter? Sport, Exercise, and Performance Psychology. 2020;9(4):532–42. <https://doi.org/10.1037/spy0000195>

34. Kutzner FLW, Förderer S, Plessner H. Regulatory fit improves putting in top golfers. Sport, Exercise, and Performance Psychology. 2013;2(2):130-7. <https://doi.org/10.1037/a0030733>

35. Lamirand M, Rainey D. Mental imagery, relaxation, and accuracy of basketball foul shooting. Percept Mot Skills. 1994;78:1229-30. <https://doi.org/10.2466/pms.1994.78.3c.1229>

36. Liang D, Chen S, Zhang W, Xu K, Li Y, Li D, et al. Investigation of a Progressive Relaxation Training Intervention on Precompetition Anxiety and Sports Performance Among Collegiate Student Athletes. Frontiers in Psychology. 2021;11(617541). <https://doi.org/10.3389/fpsyg.2020.617541>

37. Madden G, McGown C. The effect of hemisphericity, imagery, and relaxation on volleyball performance. Journal of Human Movement Studies. 1988;14:197-204.

38. Maynard IW, Hemmings B, Warwick-Evans L. The effects of a somatic intervention strategy on competitive state anxiety and performance in semiprofessional soccer players. The Sport Psychologist. 1995;9(1):51-64. <https://doi.org/10.1123/tsp.9.1.51>

39. Maynard IW, Smith MJ, Warwick-Evans L. The effects of a cognitive intervention strategy on competitive state anxiety and performance in semiprofessional soccer players. Journal of Sport and Exercise Psychology. 1995;17(4):428-46. <https://doi.org/10.1123/jsep.17.4.428>

40. McAleney PJ, Barabasz A, Barabasz M. Effects of flotation restricted environmental stimulation on intercollegiate tennis performance. Percept Mot Skills. 1990;71(3):1023-8. <https://doi.org/10.2466/PMS.71.7.1023-1028>

41. Milley KR, Ouellette GP. Putting Attention on the Spot in Coaching: Shifting to an External Focus of Attention With Imagery Techniques to Improve Basketball Free-Throw Shooting Performance. Frontiers in Psychology. 2021;12(645676). <https://doi.org/10.3389/fpsyg.2021.645676>

42. Minkler TO, Glass CR, Hut M. Mindfulness training for a college team: Feasibility, acceptability, and effectiveness from within an athletic department. J Appl Sport Psychol. 2021;33(6):609-26. <https://doi.org/10.1080/10413200.2020.1739169>

43. Mumford B, Hall C. The effects of internal and external imagery on performing figures in figure skating. Canadian Journal of Applied Sport Sciences. 1985;10(4):171-7.

44. Neumann DL, Hohnke E. Practice using performance goals enhances basketball free throw accuracy when tested under competition in elite players. J Hum Sport Exerc. 2018;13(2):296-304.

45. Noel RC. The effect of Visuo-Motor Behavior Rehearsal on tennis performance. Journal of Sport Psychology. 1980;2(3):221-6. <https://doi.org/10.1123/jsp.2.3.221>

46. Padgett VR, Hill AK. Maximizing athletic performance in endurance events: A comparison of cognitive strategies. Journal of Applied Social Psychology. 1989;19(4):331-40. <https://doi.org/10.1111/j.1559-1816.1989.tb00058.x>

47. Palao JM, García-de-Alcaraz A, Hernández-Hernández E, Ortega E. A case study of applying collective technical-tactical performance goals in elite men’s volleyball team. International Journal of Applied Sports Sciences. 2016;28(2):68-78.

48. Papaioannou A, Theodorakis Y, Ballon F, Auwelle YV. Combined effect of goal setting and self-talk in performance of a soccer-shooting task. Percept Mot Skills. 2004;98(1):89-99. <https://doi.org/10.2466/pms.98.1.89-99>

49. Plessner H, Unkelbach C, Memmert D, Baltes A, Kolb A. Regulatory fit as a determinant of sport performance: How to succeed in a soccer penalty-shooting. Psychol Sport Exerc. 2009;10(1):108-15. <https://doi.org/10.1016/j.psychsport.2008.02.001>

50. Reeves JL, Tenenbaum G, Lidor R. Choking in front of the goal: The effects of self‐consciousness training. Int J Sport Exerc Psychol. 2007;5(3):240-54. <https://doi.org/10.1080/1612197X.2007.9671834>

51. Savoy C, Beitel P. Mental imagery for basketball. Int J Sport Psychol. 1996;27:454-62.

52. Schorer J, Jaitner T, Wollny R, Fath F, Baker J. Influence of varying focus of attention conditions on dart throwing performance in experts and novices. Experimental Brain Research. 2012;217:287–97. <https://doi.org/10.1007/s00221-011-2992-5>

53. Schreiber EH. Using hypnosis to improve performance of college basketball players. Percept Mot Skills. 1991;72(2):536-8. <https://doi.org/10.2466/PMS.72.2.536-538>

54. Scott VB, Jr., Robare RD, Raines DB, Konwinski SJM, Chanin JA, Tolley RS. Emotive writing moderates the relationship between mood awareness and athletic performance in collegiate tennis players. North American Journal of Psychology. 2003;5(2):311-24.

55. Shahi MRS, Ezabadi RS, Abootalebi N, Moshiri P. The Effects of the Self-Talk Types and Task Complexity on the Accuracy of Forehand Topspin of Advanced Players. International Journal of Sports Science and Physical Education. 2020;5(2):16-20. <https://doi.org/10.11648/j.ijsspe.20200502.12>

56. Shoenfelt EL. Goal setting and feedback as a posttraining strategy to increase the transfer of training. Percept Mot Skills. 1996;83(1):176-8. <https://doi.org/10.2466/pms.1996.83.1.176>

57. Shoenfelt EL, Griffith AU. Evaluation of a mental skills program for serving for an intercollegiate volleyball team. Percept Mot Skills. 2008;107(1):293-306. <https://doi.org/10.2466/pms.107.1.293-306>

58. Simões P, Vasconcelos-Raposo J, Silva A, Fernandes HM. Effects of a process-oriented goal setting model on swimmer’s performance. Journal of Human Kinetics. 2012;32:65–76. <https://doi.org/10.2478/v10078-012-0024-6>

59. Smith D, Holmes P. The effect of imagery modality on golf putting performance. Journal of Sport and Exercise Psychology. 2004;26(3):385-95. <https://doi.org/10.1123/jsep.26.3.385>

60. Smith D, Wright C, Allsopp A, Westhead H. It’s all in the mind: PETTLEP-based imagery and sports performance. J Appl Sport Psychol. 2007;19(1):80-92. <https://doi.org/10.1080/10413200600944132>

61. Smith D, Wright CJ, Cantwell C. Beating the bunker: The effect of PETTLEP imagery on golf bunker shot performance. Research Quarterly for Exercise & Sport. 2008;79(3):385-91. <https://doi.org/10.1080/02701367.2008.10599502>

62. Spindler DJ, Allen MS, Vella SA, Swann C. Motivational-general arousal imagery does not improve decision-making performance in elite endurance cyclists. Cognition and Emotion. 2019;33(5). <https://doi.org/10.1080/02699931.2018.1529656>

63. Stamou E, Theodorakis Y, Kokaridas D, Perkos S, Kessanopoulou M. The effect of self-talk on the penalty execution in goalball. British Journal of Visual Impairment. 2007;25(3):233–47. <https://doi.org/10.1177/0264619607079800>

64. Takeuchi T, Ikudome S, Unenaka S, Ishii Y, Mori S, Mann DL, et al. The inhibition of motor contagion induced by action observation. PLoS One. 2018;13(10):e0205725. <https://doi.org/10.1371/journal.pone.0205725>

65. Vast R, Young R, Thomas PR. Emotion and automaticity: Impact of positive and negative emotions on novice and experienced performance of a sensorimotor skill. Int J Sport Exerc Psychol. 2011;9(3):227-37. <https://doi.org/10.1080/1612197X.2011.614848>

66. Velentzas K, Heinen T, Schack T. Routine integration strategies and their effects on volleyball serve performance and players’ movement mental representation. J Appl Sport Psychol. 2011;23(2):209-22. <https://doi.org/10.1080/10413200.2010.546826>

67. Wagaman JD, Barabasz AF, Barabasz M. Flotation REST and imagery in the improvement of collegiate basketball performance. Percept Mot Skills. 1991;72(1):119-22. <https://doi.org/10.2466/pms.1991.72.1.119>

68. Weinberg R, Miller A, Horn T. The influence of a self-talk intervention on collegiate cross-country runners. Int J Sport Exerc Psychol. 2012;10(2):123-34. <https://doi.org/10.1080/1612197X.2012.645135>

69. Weinberg R, Stitcher T, Richardson P. Effects of a seasonal goal-setting program on lacrosse performance. The Sport Psychologist. 1994;8(2):166-75. <https://doi.org/10.1123/tsp.8.2.166>

70. Wergin VV, Beckmann J, Gröpel P, Mesagno C. Investigating cumulative effects of pre-performance routine interventions in beach volleyball serving. PLoS One. 2020;15(1):e0228012. <https://doi.org/10.1371/journal.pone.0228012>

71. Westlund Stewart N, Hall C. The effects of cognitive general imagery use on decision accuracy and speed in curling. The Sport Psychologist. 2016;30(4):305-13. <https://doi.org/10.1123/tsp.2016-0001>

72. Whdan N. Effects of relaxation training on muscle tension and the performance level of 50m front crawl swimming. Science, Movement and Health. 2014;14(1):143-8.

73. Winter S, Collins D. Does priming really put the gloss on performance? Journal of Sport and Exercise Psychology. 2013;35(3):299-307. <https://doi.org/10.1123/jsep.35.3.299>

74. Wojcikiewicz A, Orlick T. The effects of post-hypnotic suggestion and relaxation with suggestion on competitive fencing anxiety and performance. Int J Sport Psychol. 1987;18(4):303-13.

75. Wolanin AT, Schwanhausser LA. Psychological functioning as a moderator of the MAC approach to performance enhancement. Journal of Clinical Sport Psychology. 2010;4(4):312-22. <https://doi.org/10.1123/jcsp.4.4.312>

76. Wollman N, Hill J, Lipsitz T. Effects of imagery on track and bowling performance in naturalistic settings. Percept Mot Skills. 1985;60(3):986. <https://doi.org/10.2466/pms.1985.60.3.986>

77. Woolsey TD, Woolsey CL, Strohmeyer S, Walker S, Otto W, Cheshier BC, et al. The Effect of Advanced Imagery Training on Shot Putter Performance. Journal of Contemporary Athletics. 2020;14(4):271-9.

78. Wu T-Y, Nien J-T, Kuan G, Wu C-H, Chang Y-C, Chen H-C, et al. The Effects of Mindfulness-Based Intervention on Shooting Performance and Cognitive Functions in Archers. Frontiers in Psychology. 2021;12(661961). <https://doi.org/10.3389/fpsyg.2021.661961>

79. Zervas Y, Kakkos V. Visuomotor behavior rehearsal in archery shooting performance. Percept Mot Skills. 1991;73(3, Pt 2):1183-90. <https://doi.org/10.2466/PMS.73.8.1183-1190>
