# Supplementary material for: Effects of Psychological Interventions to Enhance Athletic Performance: A Systematic Review and Meta-Analysis
Source: Sports Med. 2023 Oct 9;54(2):347–73. doi: 10.1007/s40279-023-01931-z (PMC10933186; doi:10.1007/s40279-023-01931-z)
Supplement: Supplementary file 4 — Supplementary file4 (DOCX 50 KB) [file 40279_2023_1931_MOESM4_ESM.docx]

**Supplementary information. Online Resource 4.**

*Article:* Effects of psychological interventions to enhance athletic performance: A systematic review and meta-analysis

*Journal:* Sports Medicine

*Authors:* Gustaf Reinebo, Sven Alfonsson, Markus Jansson-Fröjmark, Alexander Rozental, Tobias Lundgren

*Corresponding author:* Gustaf Reinebo, email [gustaf.reinebo@ki.se](mailto:gustaf.reinebo@ki.se) , Centre for Psychiatry Research, Department of Clinical Neuroscience, Karolinska Institutet, & Stockholm Health Care Services, Region Stockholm, Norra stationsgatan 69, SE-113 64, Stockholm, Sweden

**Study information of studies in the systematic review**

| **Study** | **Sport and sample** | **Level of performance** | **Psychological intervention** | **Intervention duration** | **Design** | **Performance outcome** | **Other outcomes** |
| --- | --- | --- | --- | --- | --- | --- | --- |
| Abdoli et al. [1] | Basketball; Male; N = 20; Mean age 23,5 | National | Self-talk (motivational vs. instructional) | Instructions prior to performance | RCT (only active group comparisons) | Basketball free throw performance (O) | Movement coordination variability |
| Ahmadzadeh et al. [2] | Shooting (10m air rifle); Male; N = 24; Mean age 24 | National | Neuro-lingustic programming (NLP) | Eight 2,5 hr sessions (total time 20 hr) | Controlled trial | Air rifle shooting scores (under both ordinary and pressure conditions) (O) | Ottawa Mental Skill Assessment Tool-3 (OMSAT-3) |
| Anderson et al. [3] | Ice hockey; Male; N = 22; Age range 18-22 | University | Goal-setting, Feedback, and Praise (in different intervention phases) | The three intervention phases were administered over the course of a season for two consecutive seasons | Within-group time series design with three different experimental phases (ABCD) | Hit-rate (O); Penalty time (O); Wins-losses (O) | None |
| Anshel [4] | Tennis; Female; N = 12; Mean age 19,8 | University | COPE program (deep breathing relaxation, attribution training, VMBR, assertiveness training, thought stopping, task simulation and flooding) | Six 1 hr sessions twice a week during three weeks (total time 6 hr), with homework assignments | Uncontrolled trial (participants were randomized and exposed to three different amounts of stress pre-intervention, although all participants received the same intervention) | Tennis stroke accuracy (O) | The Multiple Affect Adjective Checklist |
| Ashford and Jackson [5] Experiment 1 | Field hockey; Male & Female; N = 34; Mean age 22,05 | University | Attentional (internal/skill focus) vs. Priming | Instructions prior to performance | Crossover design with three counterbalanced conditions (two experimental, one control) | Completion time in a dribbling task (O); Lateral displacement in a dribbling task (O) | The cognitive and somatic subscales from the revised Competitive State Anxiety Inventory (CSAI-2R); Perceived pressure ratings (low & high pressure conditions were used) |
| Ashford and Jackson [5] Experiment 2 | Field hockey; Male & Female; N = 30; Mean age 21,5 | University | Attentional (internal/skill focus) vs. Priming (varied between participants as either positive, negative prime or neutral) | Instructions prior to performance | Crossover design with three counterbalanced conditions (two experimental, one control) | Completion time in a dribbling task (O); Lateral displacement in a dribbling task (O) | The cognitive and somatic subscales from the revised Competitive State Anxiety Inventory (CSAI-2R); Perceived pressure ratings (low & high pressure conditions were used) |
| Bakker and Kayser [6] | Field hockey; Female; N = 30; Mean age 21,7 | Regional | Relaxation, concentration & imagery | Three sessions of approximately 2 hr over seven weeks (total time 6 hr), participants were also instructed to listen to exercise audio tapes for about 10 min/day | Controlled trial (experimental vs. placebo control vs. control) | Field hockey penalty stroke performance (O) | Competetive State Anxiety Inventory-II (CSAI-II); Credibility questionnaire; Heart rate while listening to intervention or placebo audio tapes |
| Barker et al. [7] | Soccer; Male & Female; N = 59; Mean age 21,34 | University | Hypnosis | Three 45 min group sessions, the last session was conducted between post and follow-up (total time 2hr 15 min) | RCT | A soccer wall-volley task (O) | Soccer Wall-Wolley Self-Efficacy Questionnaire (SWVSEQ); Perceived Value of Training Form; Credibility questionnaire |
| Blair et al. [8] | Soccer; Female; N = 22 ^a^; Age nr ^a^ | University | Imagery | Bi-weekly sessions of 15 min during six weeks (total time 3 hr) | RCT | Time (O) and accuracy (O) of completing a soccer task | Post-evaluation including credibility questions and other experiences of the received training |
| Boutcher and Crews [9] | Golf; Male & Female; N = 12; Mean age 20,34 | University | Pre-performance routine (attentional focus on a series of specific cues, e.g., behavioral and verbal cues) | Routine practice 20 min sessions, four times/week during six weeks (total time 8 hr) | RCT | Number of holed golf putts (O); Distance from hole (O) | Putter grounding time; Credibility check |
| Burton [10] | Swimming; Male & Female; N = 65; Mean age 20,25 | University | Goal-setting | A season long program: 2 hr group orientation session followed by another five group sessions, and individual weekly sessions (number of individual sessions range 2-11, M = 5,4) | Controlled trial | Performance scores based on race times (O) | Sport Competition Anxiety Test (SCAT); Sport Confidence Inventory (SCI); Achievement Orientation Questionnaire (AOQ); Competitive State Anxiety Inventory-2 (CSAI-2); Self-ratings regarding several aspects of the competitive experience |
| Caudill et al. [11] Experiment 1 | Track (100-yard dash; 60-yard hurdles); Male & Female; N = 16; Age nr | University | Self-determined psyching-up strategy | 1 min prior to race | Counterbalanced crossover design with one experimental and one control condition (three races in each condition) | Sprint times (O) | Type of used psyching-up strategy: preparatory arousal (7 %), imagery (16 %), self-efficacy statements (25 %), attentional focus (16 %), relaxation/distraction (25 %), religious beliefs (11 %) (50 % of participants used more than one strategy) |
| Caudill et al. [11] Experiment 2 | Track (sprint and hurdles); Male & Female; N = 16; Age nr | University | Self-determined psyching-up strategy | 1 min prior to race | Counterbalanced crossover design with one experimental, one attention-placebo-control and one control condition (two races in each condition) | Sprint times (O) | Type of used psyching-up strategy: preparatory arousal (15 %), imagery (10 %), self-efficacy statements (20 %), attentional focus (25 %), relaxation/distraction (20 %), religious beliefs (10 %) (40 % of participants used more than one strategy) |
| Connolly and Janelle [12] Experiment 1 | Rowing; Female; N = 9; Mean age 19,9 | University | Attentional focus intervention (associative vs. dissociative) | Instruction/protocol during performance | Randomized counterbalanced crossover design with two experimental conditions | Meters rowed on a rowing machine (O) | The Borg rating of perceived exertion scale; Heart rate |
| Connolly and Janelle [12] Experiment 2 | Rowing; Male & Female; N = 24; Mean age 19,95 | University | Attentional focus intervention (internal association; internal dissociation; external association; external dissociation) | Instruction/protocol during performance | Crosssover design with baseline followed by four counterbalanced experimental conditions | Meters rowed on a rowing machine (O) | The Borg rating of perceived exertion scale; Heart rate |
| Couvillion and Fairbrother [13] | Jump roping; Gender nr; N = 15 ^a^; Age nr ^a^ | National, International | Attentional (internal upper body; external upper body; internal lower body; external lower body) | Instructions prior to performance | Latin square crossover design with baseline followed by four counterbalanced experimental conditions | Number of jumps (O); Number of errors (O) | Credibility questions |
| Dahm and Rieger [14] | Dart; Male; N = 20 ^a^; Mean age 29,9 | Regional | Imagery | Instructions prior to performance | Crossover counterbalanced design with one experimental and one control condition | Dart throwing performance (O) | Comparisons between imagined and actual performance (which is the main focus of the study) |
| Daneshfar et al. [15] | BMX; Male & Female; N = 13; Mean age 19,2 | National | Imagery (PETTLEP) | Imagery practice twice a day every second day for four weeks (total time approx 320 min) | Randomized crossover design | BMX racing time (O) | Vertical jump height; power output; Heart rate; Rate of perceived exertion; Movement imagery questionnaire (MIQ-3) |
| Daw and Burton [16] | Tennis; Male & Female; N = 26; Age range 18-23 | University | PST program (goal-setting, imagery, arousal regulation) | Approximately 1 year and active phases were during fall and spring seasons (planning phases during spring, fall season and spring season), with twenty-seven group sessions in total for both fall and spring seasons and mean number of individual sessions were 2,46 (fall season) and 4,75 (spring season). | Controlled trial | Double-fault percentage (O); first-serve percentage (O); unforced-error percentage (O), "aggressive margin" which is a summarized measure (O); self-rated performance (S) | Trait-Sport Confidence Inventory (TSCI); State Sport-Confidence Inventory (SSCI); Sport Competition Anxiety Test (SCAT); Mental Readiness Form (MRF); Pre- and Post match questionnaires |
| de Matos et al. [17] | Triathlon; Male & Female; N = 27; Mean age 32,7 | Regional, national and international | Self-talk (motivational)^a^ | Instructions prior to performance | RCT^a^ | Swimming time performance (O) | Rating of perceived effort (Borg scale); Pacing; Brunel Mood Scale (BRUMS); Outcome expectancy; Credibility and negative side effect question |
| Donohue et al. [18] | Cross country running; Female; N = 6; Age range 18-21 | University | Motivational statements vs. Questions about thoughts and bodily sensations/feelings | Instructions 5 min prior to perfromance | Randomized latin square crosssover design with baseline followed by two ^a^ counterbalanced experimental conditions | Running times (O) | Credibility questions |
| Gray and Fernandez [19] | Basketball; Female; N = 6; Age range 18-21 | University | VMBR with video-modeling | Five sessions of 30 min (total time 2,5 hr) | Uncontrolled trial | Basketball shooting performance (consecutive free throws, non-consecutive free throws, and outside shots) (O) | None |
| Gross et al. [20] | Basketball; Female; N = 22; Age nr | University | MAC vs. PST program (relaxation, stress management, positive thought control, self-regulation, mental rehearsal, concentration, energy control) | For both interventions: Seven 1h sessions over six weeks (total time 7 hr) | RCT (only active group comparisons) | Coach-rated performance (Sport Performance Questionnaire, SPQ) (S) | Psychological flexibility (AAQ-II); Psychiatric symptoms and distress (CCAPS-62); Emotions regulation (DERS); Mindful awareness and attention (MAAS) |
| Gröpel [21] | Basketball; Male & Female; N = 62; Mean age 25,02 | Regional | Attentional (internal self-focus) | Instructions prior to performance | Uncontrolled trial | Basketball free throw performance (O) | None |
| Guillot et al. [22] | High jump; Male & Female; N = 12; Mean age 20,42 | National | Imagery (motor imagery: dynamic vs. motionless) | Information session regarding imagery one week prior to experiment, then instructions/script prior to performance | Randomized controlled crossover design (two experimental conditions) | High jump performance (O); Expert rating of performance technique (S) | Motor imagery time and actual time; Imagery vividness |
| Guillot et al. [23] | Basketball; Female; N = 10; Mean age 22,7 | National | Imagery (motor) | Imagery practice 10-12 min twice a week during six weeks (total time approximately 144 min) | Pre-post crossover design (imagery + physical training vs. physical training only vs. no-practice-control) | Coach rated performance (S); Self-rated performance (S) | Imagery duration |
| Hall and Erffmeyer [24] | Basketball; Female; N = 10; Age nr | University | VMBR + video-modeling vs. Progressive relaxation + Imagery | VMBR + video-modeling: Two-week training period, first week - five days of 30 min of progressive relaxation and visual imagery, second week - five days of 20 min progressive relaxation and visual imagery followed by another 20 min of video modeling and imagery (total time 5 hr 50 min); Progressive relaxation + Imagery: Two-week training period, first week - five days of 30 min of progressive relaxation and visual imagery, second week - five days of 40 min progressive relaxation and visual imagery (total time 5 hr 50 min) | RCT (only active group comparisons) | Free throw performance (O) | Qualitative data from an imagery questionnaire |
| Halperin et al. [25] | Boxing; Male; N = 15; Mean age 21 | National | False performance feedback (false-positive vs. false-negative vs. false-neutral) | Information received prior to performance | Randomized crossover design with baseline followed by three counterbalanced experimental conditions | Punching force (O); Pacing (force over time) (O) | Borg rating of perceived exertion (RPE) |
| Hammoudi-Nassib et al. [26] | Sprint; Male; N = 16; Mean age 20,6 | Regional | Imagery vs. Preparatory arousal vs. Attention placebo (control) vs. Distraction (control) | 30 s duration and administered prior to performance | Randomized counterbalanced crossover design with two experimental and two control conditions | Sprint times (O) | None |
| Holm et al. [27] | Swimming; Male & Female; N = 36 ^a^; Age nr | University | Stress management course based on cognitive and behavioral interventions | Seven 2 hr weekly sessions + home work assignments | RCT | Swimming times (O) | Psychological skills; Anxiety; Academic performance |
| Horton and Shelton [28] | Wresling; Male; N = 4; Age nr | University | Cognitive and behavioral techniques | Two group sessions | Uncontrolled trial | Wrestling performance (records of dual meets) (O) | None |
| Hut et al. [29] | Track and field; Male & Female; N = 30; Mean age 19,52 | University | Mindfulness Sport Performance Enhancement (MSPE) vs. PST (relaxation, self-talk, imagery) | Six 1h sessions over six weeks (both interventions) | RCT (only active group comparisons) | Self rating of overall performance (S) | Short Dispositional Flow Scale (SDFS-2); Core Dispositional Flow Scale-2 (CDFS-2); Depression Anxiety Stress Scales (DASS-21); Sport Anxiety Scale-2 (SAS-2); Philadelphia Mindfulness Scale (PHLMS); Mindfulness Inventory for Sports (MIS); Difficulties in Emotion Regulation Scale-Short Form (DERS-SF); Brief Experiential Avoidance Questionnaire (BEAQ); Sport Rating Form (SRF); Program Evaluation Questionnaire (PEQ) including credibility questions |
| Ille et al. [30] | Sprint; Male; N = 8 ^a^; Age nr ^a^ | Regional to International | Attentional (external focus) ^a^ | Instructions prior to performance | Crossover design with baseline followed by counterbalanced experimental conditions ^a^ | Total sprint time 10m (O) (also divided into three performance phases: reaction time; block clearance time; running time) | None |
| Kacperski and Hall [31] Experiment 1 | Table tennis & Track and field (throwers and long jumpers); Male & Female; N = 30; Mean age 21,2 | University | Imagery (with high construal level vs. low construal level) | Imagery once prior to performance | Crossover design with baseline followed by two counterbalanced experimental conditions | Table tennis - wins and losses (O); Throw and long jump - failure or successful execution of throw/long jump (O); Self reporting of perceived success (S) | Imagery experience; Credibility question |
| Kacperski and Hall [31] Experiment 2 | Soccer & Badminton; Male & Female; N = 32; Mean age 19,2 | University | Construal levels manipulated through verbal frames (desirability vs. feasibility) | Verbal instructions/questions prior to performance | Counterbalanced crossover design with two experimental conditions | Badminton - wins and losses (O); Soccer - penalty kicks (O); Reported perceived success (S) | Credibility question |
| Kanthack et al. [32] Experiment 1 | Basketball; Male; N = 6; Mean age 18,4 | State | Imagery (static motor imagery vs. dynamic motor imagery) | Two static imagery sessions/week of 5 min for four weeks as a familiarization prior to the experiment, then five motor imagery trials prior to performance (for both conditions) | Counterbalanced crossover design with two experimental and one control condition | Basketball free-throw performance (O) | Heart rate; Exertion perception (OMNI); Imagery vividness and duration |
| Kanthack et al. [32] Experiment 2 | Basketball; Male; N = 6; Mean age 18,4 | State | Imagery (static motor imagery vs. dynamic motor imagery) | Two static imagery sessions/week of 5 min for four weeks as a familiarization prior to the experiment, then five motor imagery trials prior to performance (for both conditions), also the participants had the imagery experience from experiment 1 | Counterbalanced crossover design with two experimental and one control condition (all conditions were performed under physical exhaustion as compared to experiment 1) | Basketball free-throw performance (O) | Heart rate; Exertion perception (OMNI); Imagery vividness and duration |
| Klatt and Noël [33] Experiment 1 | Soccer; Male; N = 40; Mean age 23,28 | Regional | Regulatory focus instructions (promotion vs. prevention) | Instructions prior to performance | Controlled trial (only active comparisons) | Soccer penalty kicks (O) | Chronic regulatory focus orientation (German version of the Lockwood Scale) |
| Klatt and Noël [33] Experiment 2 | Beach volleyball; Male & Female; N = 40; Mean age 21,98 | National | Regulatory focus instructions (promotion vs. prevention) | Instructions prior to performance | Controlled trial (only active comparisons) | Beach volleyball serve accuracy (O) | Chronic regulatory focus orientation (German version of the Lockwood Scale) |
| Kutzner et al. [34] | Golf; Male & Female; N = 30; Mean age 21,63 | National | Regulatory focus instructions (promotion vs. prevention) | Instructions prior to performance | RCT (only active group comparisons) | Golf putting performance (O) | Chronic regulatory focus orientation (adapted version of the Lockwood Scale) |
| Lamirand and Rainey [35] | Basketball; Female; N = 18; Mean age 19,2 | University | Imagery vs. Relaxation (diaphragmatic breathing) | For both interventions: 5 min on audiotape per session, and a total of four sessions over 3 weeks (total time 20 min) | Controlled trial | Free throw performance (O) | None |
| Liang et al. [36] | Track and field; Male & Female; N = 24; Mean age 20,91 | University | Progressive relaxation | 30 min sessions twice a week during 1 month | Controlled trial | Reaching best personal record or not (O) | Competitive State Anxiety Inventory-2 (CSAI-2); State-Trait Anxiety Inventory (STAI); Eysenck Personality Questionnaire - Revised Short Scale for Chinese (EPQ-RSC) |
| Madden and McGown [37] | Volleyball; Female; N = 17; Age nr | University | VMBR | Twenty sessions of 25 min over ten weeks (total time 8 hr 20 min) | RCT | Volleyball forearm pass test (O); Serve receive efficiency and on target statistics in games (O) | None |
| Maynard, Hemmings and Warwick-Evans [38] | Soccer; Male; N = 22; Mean age 24,3 | County | Relaxation (applied relaxation) | Eight weekly group sessions + relaxation practice twice a day as homework assignment | Controlled trial | Peformance rating by experts (S) | Competitive State Anxiety Inventory-2 (CSAI-2) |
| Maynard, Smith and Warwick-Evans [39] | Soccer; Male; N = 24; Mean age 23,13 | County | Positive thought control (PTC) | Twelve weekly sessions: first session was a group introduction session, then weekly individual sessions of 20 min (approximately 4 hr in total) | Controlled trial | Expert ratings of players decision making and physical skills in soccer (S) | Competitive State Anxiety Inventory-2 (CSAI-2), and also a scale used in conjunction to CSAI-2 that investigated whether symptoms were facilitative or debilitative to performance or not |
| McAleney et al. [40] | Tennis; Male & Female; N = 20; Age range 18-22 | University | Imagery with flotation REST vs. Imagery | Six sessions in three weeks, 50 min/session (imagery message was 8 min; total imagery time 56 min; total intervention time 5 hr) | RCT (only active group comparisons) | Tennis performance (three measures: serve performance; key shots; points won/lost) (O) | None |
| Milley and Ouellette [41] | Basketball; Male & Female; N = 25; Mean age 19,92 | University | Imagery (PETTLEP, external focus orientation)^a^ | Imagery prior to performance and reminders of attention of focus during performance | Randomized counterbalanced crossover design^a^ | Basketball free throw performance (O) | Flow State Scale-2 (FSS-2); Movement Imagery Questionnaire (MIQ) |
| Minkler et al. [42] | Lacrosse; Female; N = 30; Mean age 19,63 | University | Mindfulness Sport Performance Enhancement (MSPE) | Six weekly 60-75 min sessions | Uncontrolled trial | Coach rating of overall performance (S); Self rating of overall performance (S) | Short Dispositional Flow Scale-2 (S DFS-2); Core Dispositional Flow Scale-2 (C DFS-2); Sport Anxiety Scale-2 (SAS-2); Philadelphia Mindfulness Scale (PHLMS); Mindfulness Inventory for Sport (MIS); Difficulties in Emotion Regulation Scale-Short Form (DERS-SF); Sport Rating Form (SRF); Coach’s Rating Form (CRF); Correlations between change in psychological measures and objective athletic performance; Program Evaluation Questionnaire (PEQ) including credibility questions |
| Mumford and Hall [43] | Figure skating; Male & Female; N = 16 ^a^; Age nr ^a^ | Sectional or above | Imagery (three imagery conditions are mixed in the included subsample: internal kinestethic, internal visual and external visual) | Four sessions of 1 hr (total time 4 hr) | Uncontrolled trial ^a^ | Figure skating task (O) | Confidence of performance rating; Movement imagery question (MIQ); Credibility questions |
| Neumann and Hohnke [44] | Basketball; Male; N = 30; Mean age 24,67 | State, National | Goal-setting | Instructions prior to performance | RCT | Basketball free throw performance (O) | Competitive State Anxiety Inventory-2 (CSAI-2); Borg’s Ratings of Perceived Exertion Scale |
| Noel [45] | Tennis; Male; N = 6 ^a^; Age nr ^a^ | County | VMBR | One initial group session 10 days prior to the tournament followed by self-practice with audiotapes focusing on both relaxation and visualization exercises leading up to the tournament (total time approximately 6 hr) | RCT | Ratio of winning to error shots (O); Accuracy of first and second serve (S); Self-reporting of tennis serve performance (S) | Self-report of relaxation during the match; Credibility question |
| Padgett and Hill [46] Experiment 2 | Running; Male; N = 12; Age nr | University | Attentional (dissociation vs. external focus) | Instructions prior to performance | Randomized counterbalanced crossover design with two experimental and one control condition | Running time (O); Estimate of running time (S) | Self-reported effort |
| Palao et al. [47] | Volleyball; Male; N = 14; Mean age 23,3 | National | Goal-setting program for a team | The intervention lasted for the second part of the season (11 games). A poster with goals and to what extent they were acheived was put up in the locker room. An introductory meeting was held and also weekly updates on current objectives. | Uncontrolled trial | Wins/losses (O); points (O); receptions (O); attacks (O); counter-attacks (O); serves (O); blocks (O); digs (O) | Qualitative interviews about players, coaches and the psychologists experiences of the intervention |
| Papaioannou et al. [48] | Soccer; Male; N = 41; Mean age 23,8 | Regional, National | Goal-setting vs. Self-talk vs. Goal-setting + Self-talk | For all conditions: short introduction at the first experimental trial and then repetition at the following experimental trials (duration nr) | Controlled trial (three experimental conditions and one non-active control) | Soccer shooting accuracy (O) | None |
| Plessner et al. [49] | Soccer, Male; N = 20; Mean age 28,05 | Regional | Regulatory focus instructions (promotion vs. prevention) | Instructions prior to performance | RCT (only active group comparisons) | Soccer penalty performance (O) | Chronic regulatory focus orientation (adapted version of the Lockwood Scale); Coach-rated penalty shooting ability (rated prior to performance) |
| Reeves et al. [50] | Soccer; Female; N = 18 ^a^; Age range 18-22 | University | Attentional (internal self-focus vs. external extraneous focus) ^b^ | Instructions prior to performance | RCT (two experimental conditions and one control) | Perceived performance (S)* | Psychological Grid (questions regarding arousal, confidence etc. when performing); Perceived pressure |
| Savoy and Beitel [51] | Basketball; Female; N = 10; Mean age 19,3 | University | Imagery | Intervention phases conisted of 9 + 9 + 5 games, during these phases athletes were instructed to image 20 successful foul shots at night during 20 of 30 days and keep logs of it | Reversed within-group design (A1B1A2B2A3B3) | The free throw percentage for the team (O) | None |
| Schorer et al. [52] | Dart; Male; N = 6 ^a^; Mean age 33,2 | National | Attentional (internal1 vs. internal2 vs. external) | Instructions prior to performance | Randomized crossover design with baseline followed by three counterbalanced experimental conditions | Dart throwing accuracy (O) | Dart throwing kinematics; Motivation to complete the experiment; Comprehensibility of task instructions; Credibility/hindrance rating and ranking of each condition |
| Schreiber [53] | Basketball; Male & Female; N = 28; Mean age 20 | University | Hypnosis | Eight sessions in total during four weeks, 2 sessions/week - one individual (20 min) and one in group (45 min), total time approximately 4 hr and 20 min, also players were asked to complete a writing assignment at home writing about their perceptual experiences from each game | RCT | Basketball scoring points (O) | None |
| Scott et al. [54] | Tennis; Male & Female; N = 13; Mean age 21,27 | University | Emotive writing intervention | Three consecutive days of 15 min writing sessions (total time approximately 45 min) | Interrupted group time series design | Tennis match performance (wnner-to-unforced-error ratio, first serve percentage, forced error rate) (O) | Measure Instrument for Tennis Performance (MITP); Scott McIntosh Rumination Inventory |
| Shahi et al. [55] | Table tennis; Gender nr; N = 30; Mean age nr | Regional, National | Self-talk (motivational)^a^ | Three weekly sessions for two weeks + Instructions prior to performance | RCT (two experimental conditions and one control) | Table tennis stroke accuracy (O) | None |
| Shoenfelt [56] | Baketball; Female; N = 12; Age nr | University | Goal-setting with performance feedback | nr | Controlled trial | Free throw performance (O); Field goal performance (O) | None |
| Shoenfelt and Griffith [57] | Volleyball; Female; N = 11; Mean age 20 | University | PST program (relaxation, imagery, attentional focus, goal setting, behavioral modeling) | Eight sessions of 1 hr over a two-week period (total time 8 hr) | Uncontrolled trial | Volleyball serve performance (O) | Mental skills utilization and correlation with serve performance; Serve-specific self-efficacy and correlation with serve performance; Correlation between imagery utilization and serve performance |
| Simões et al. [58] | Swimming; Male & Female; N = 9; Mean age 17,44 | National, International | Goal-setting | 30 min weekly sessions during a season (total of 36 sessions, total time 18 hr) | Within-group time series design | Swimming times (O) | None |
| Smith and Holmes [59] | Golf; Male; N = 40; Mean age 25,3 | County | Imagery (three imagery conditions and control: written script vs. video vs. audio vs. control) | Imaging 15 successful putts/day for six weeks | RCT (three experimental conditions and one control) | Golf putting performance (O) | Vividness of Movement Imagery Questionnaire (Imagery ability) |
| Smith et al. [60] | Field hockey; Male & Female; N = 48; Mean age 20,37 | University | Imagery (three imagery conditions and control: PETTLEPP vs. clothing imagery vs. traditional imagery vs. control) | Daily imagery sessions for 6 weeks and each imagery sessions lasted around 5 min (10 imagined penalty flicks), total time approximately 3,5 hr | RCT (three experimental conditions and one control) | Field hockey penalty stroke performance (O) | Movement Imagery Queationnaire-Revised (Imagery ability) |
| Smith et al. [61] | Golf; Male; N = 34; Age nr | County, International | Imagery (two imagery conditions and two control conditions: PETTLEP vs. PETTLEP + physical practice vs. physical practice vs. control, reading golf literature) | Two sessions/week for 6 weeks. Each session was to either imagine 15 bunker shots (imagery group), practice 15 bunker shots (physical practice group) etc., with all conditions taking the same amount of time. | RCT (two experimental conditions and two control) | Golf bunker shots (O) | Movement Imagery Queationnaire-Revised (Imagery ability); Credibility questions |
| Spindler et al. [62] | Cycling; Male & Female; N = 54; Mean age 24,07 | National | Positive imagery (PETTLEP) vs. Negative imagery (PETTLEP) | During performance (for both experimental conditions) | RCT (only active group comparisons) | Physiological duress (Functional threshold power test) (O) | Stroop task; The Sport Imagery Ability Questionnaire (SIAQ); Sport Emotion Questionnaire (SEQ) |
| Stamou et al. [63] | Goalball; Female; N = 6; Mean age 33 | International | Self-talk (motivational) ^a^ | A meeting prior to the experimental trial introducing the self-talk concept and learned the self-talk sequence, and later reminded prior to performance | Crossover design ^a^ | Goalball penalty shots (O) | Credibility questions |
| Takeuchi et al. [64] Experiment 2 | Hammer; Male & Female; N = 6; Mean age 20,8 | National | Self-talk for attentional focus (internally directed vs. externally directed) | Instructions during video model observations prior to performance | Counterbalanced crossover design with two experimental conditions | Hammer throw aiming performance (O) | None |
| Vast et al. [65] | Basketball; Male & Female; N = 40 ^a^; Mean age 23,4 | State, National or International | Word semantic task | Exposure during the experimental condition | Crossover design with baseline followed by three counterbalanced experimental conditions | Free throw performance (O); Self-rating of the free throw perfomance (S) | Valence of feelings; Word semantic performance |
| Velentzas et al. [66] | Volleyball; Female; N = 30; Mean age 23,63 | Regional | Imagery (combined with a preceding 5 min breath control and relaxation exercise) | Seven-week training program with 2 sessions/week | Controlled trial (two experimental conditions and one control) | Volleyball serve performance (O) | Vividness of Movement Imagery Questionnaire (VMIQ); Players mental representation profiles (Structure Dimensional Analysis-Motoric, SDA-M); Movement kinematics (hand and ball velocities) |
| Wagaman et al. [67] | Basketball; Male; N = 22; Mean age nr | University | Imagery with flotation REST vs. Imagery | For both interventions: 6 sessions over a five week period, each session approximately 30 min (total time 3 hr) | RCT (only active group comparisons) | Basketball performance (The PERF score) (O); The Performance Evalution Questionnaire (blinded coach rating) (S) | The Performance Questionnaire (Credibility questionnaire) |
| Weinberg et al. [68] | Running; Male & Female; N = 54 ^a^; Age nr ^a^ | University | Self-talk (motivational self-set vs. motivational assigned vs. combined motivational/instructional self-set vs. combined motivational/instructional assigned) ^a^ | Individually adapted audio file approximately 3 min in length (two full cycles of 12 statements) and listened to once 5 min prior to performance | Controlled trial | Running times (O) | Credibility questions |
| Weinberg et al. [69] | Lacrosse; Male; N = 24; Age range 18-21 | University | Goal-setting | An individual meeting in the beginning of season to set goals, then manipulation checks pre-season, mid-season and post-season (the manipulation checks may have served as a reminder for the participants) | Controlled trial | Lacrosse performance (assists (O); offensive ground balls (O); defensive ground balls (O); defensive clears (O)) | None |
| Wergin et al. [70] | Beach volleyball; Male & Female; N = 54; Mean age 27,72 | University, National | Pre-Performance Routine (PPR) vs. PPR + dynamic handgrip | Prior to performance | RCT (two experimental conditions and two control) | Beach volleyball serve accuracy (O) | Mental Readiness Form-3 (MRF-3) |
| Westlund Stewart and Hall [71] | Curling; Male & Female; N = 11; Mean age 21,09 | University | Imagery | Six-week intervention, each session 10-15 min (number of total sessions nr) | Uncontrolled trial | Curling strategy ability and decision making (S); Response time in curling decision making (O) (a computer-based strategy assessment tool were used for both outcomes) | The Movement Imagery Questionnaire-Revised (MIQ-R); The Sport Imagery Questionnaire (SIQ) |
| Whdan [72] | Swimming; Female; N = 6; Mean age 20,32 | University | Relaxation | Three times/week during 8 weeks | Uncontrolled trial | Performance of 50 m crawl (O) | Grid Concentration Test (attention focus); Tension degree |
| Winter and Collins [73] | Field hockey; Male & Female; N = 18; Mean age 29,61 | County to International | Imagery (PETTLEPP) vs. Priming vs. Attentional (skill focus/internal) | Instructions/exposure prior to performance | A quasi-randomized latin square crossover design with baseline followed by three counterbalanced experimental conditions | Dribbling performance: performance time (O) and lateral displacement (O) | Debrief questions after each experimental conditions |
| Wojcikiewicz and Orlick [74] | Fencing; Male & Female; N = 42; Age nr | National | Hypnosis vs. Relaxation vs. Control | For both interventions: familirization session one week before the competition. Intervention took place at the competition site and hypnosis or relaxation was induced 30-60 min before the competition prior to warm up (total time approximately 20-25 min including familirization session) | RCT (two experimental conditions and one control) | Fencing performance: hits scored and received (O), bouts won and lost (O), final placing (O) | Competition/performance related anxiety; Estimated level of task difficulty |
| Wolanin and Schwanhausser [75] | Field hockey & Volleyball; Female; N = 20; Age nr | University | MAC in group (two intervention groups separated by sport type) | Seven weekly sessions of approximately 40 min (total time 4 hr and 40 min) | Controlled trial | Coach-rated performance (S) | The Metacognitions Questionnaire (MCQ); The Generalized Anxiety Disorder Scale (GADS) |
| Wollman et al. [76] Experiment 1 | Cross-country running; Male; N = 14; Age nr | University | Imagery + Relaxation vs. Relaxation | nr | RCT (only active group comparisons) | Running time (O); Coaches rating of running technique (S) | Runners confidence; Working towards goals; Coaches rating of "desire" |
| Wollman et al. [76] Experiment 2 | Bowling; Gender nr; N = 13; Age nr | Regional | Relaxation + Imagery + Attentional focusing + Positive mental attitude | A lecture and listened to a tape | Controlled trial | Bowling scores (O) | None |
| Woolsey et al. [77] | Shot put; Male & Female; N = 10; Age range 19-23 | University | Imagery (PETTLEP) | 5 weekly sessions for 3 weeks + prior to performance | Uncontrolled trial | Shot put throw distance (O); Peak force (O); Release velocity (O) | Release angle; Release height |
| Wu et al. [78] | Archery; Male & Female; N = 23; Mean age 20,64 | University | Mindfulness-Based Peak Performance (MBPP) program | Eight 60 min sessions over 4 weeks (twice per week) + 20 min daily home practice + 15 min mindfulness practice prior to regular training | Uncontrolled trial | Archery shooting performance (O) | Stroop task; Chinese Mindful Attention Awareness Scale (CMAAS); Chinese Mindfulness Inventory in Sport (CMIS); Chinese Multidimensional Rumination Questionnaire (CMRQ) |
| Zervas and Kakkos [79] | Archery; Male & Female; N = 18; Mean age 25 | Regional, National, International | VMBR | Two sessions/week during 4 weeks (total time 9,5 hr), and participants were also asked to practice daily at home | Controlled trial | Archery shooting scores (O) | Competitive State Anxiety Inventory-2 (CSAI-2); Self-report of tension/relaxation; Self-report of feelings |

*Note.* Study information of meta-analytic studies are shown separately in Table 2. nr = not reported; MAC = Mindfulness Acceptance Commitment approach; PST = Psychological skills training; REST = Flotation Restricted Environmental Stimulation; VMBR = Visuo-motor behavior rehearsal; RCT = Randomized controlled trial.

^a^ additional study characteristics exist that did not meet eligibility criteria. ^b^ conditions had other names in the article but are renamed to make the intervention content and comparison between studies clearer.

**References**

1. Abdoli B, Hardy J, Riyahi JF, Farsi A. A closer look at how self-talk influences skilled basketball performance. The Sport Psychologist. 2018;32(1):9-15. <https://doi.org/10.1123/tsp.2016-0162>

2. Ahmadzadeh S, Badami R, Aghaei A. The Effectiveness of Neuro-Linguistic Programming (NLP) on Shooters’ Mental Skills and Shooting Performance. Iran J Psychiatry Behav Sci. 2019;13(3):e84124. <https://doi.org/10.5812/ijpbs.84124>

3. Anderson DC, Crowell CR, Doman M, Howard GS. Performance posting, goal setting, and activity-contingent praise as applied to a university hockey team. Journal of Applied Psychology. 1988;73(1):87–95. <https://doi.org/10.1037/0021-9010.73.1.87>

4. Anshel MH. Toward validation of a model for coping with acute stress in sport. Int J Sport Psychol. 1990;21(1):58-83.

5. Ashford KJ, Jackson RC. Priming as a means of preventing skill failure under pressure. Journal of Sport and Exercise Psychology. 2010;32(4):518-36. <https://doi.org/10.1123/jsep.32.4.518>

6. Bakker FC, Kayser CS. Effect of a self-help mental training programme. Int J Sport Psychol. 1994;25(2):158-75.

7. Barker J, Jones M, Greenlees I. Assessing the immediate and maintained effects of hypnosis on self-efficacy and soccer wall-volley performance. Journal of Sport and Exercise Psychology. 2010;32(2):243–52. <https://doi.org/10.1123/jsep.32.2.243>

8. Blair A, Hall C, Leyshon G. Imagery effects on the performance of skilled and novice soccer players. Journal of Sports Sciences. 1993;11(2):95-101. <https://doi.org/10.1080/02640419308729971>

9. Boutcher SH, Crews DJ. The effect of a preshot attentional routine on a well-learned skill. Int J Sport Psychol. 1987;18(1):30-9.

10. Burton D. Winning isn’t everything: Examining the impact of performance goals on collegiate swimmers’ cognitions and performance. The Sport Psychologist. 1989;3(2):105–32. <https://doi.org/10.1123/tsp.3.2.105>

11. Caudill D, Weinberg R, Jackson A. Psyching-up and track athletes: A preliminary investigation. Journal of Sport Psychology. 1983;5(2):231-5. <https://doi.org/10.1123/jsp.5.2.231>

12. Connolly CT, Janelle CM. Attentional strategies in rowing: Performance, perceived exertion, and gender considerations. J Appl Sport Psychol. 2003;15(3):195-212. <https://doi.org/10.1080/10413200305387>

13. Couvillion KF, Fairbrother JT. Expert and novice performers respond differently to attentional focus cues for speed jump roping. Frontiers in Psychology. 2018;9:2370. <https://doi.org/10.3389/fpsyg.2018.02370>

14. Dahm SF, Rieger M. Is imagery better than reality? Performance in imagined dart throwing. Human Movement Science. 2019;66:38-52. <https://doi.org/10.1016/j.humov.2019.03.005>

15. Daneshfar A, Petersen CJ, Gahreman DE. The effect of 4 weeks motor imagery training on simulated bmx race performance. Int J Sport Exerc Psychol. 2022;20(2):644–60. <https://doi.org/10.1080/1612197X.2020.1869801>

16. Daw J, Burton D. Evaluation of a comprehensive psychological skills training program for collegiate tennis players. The Sport Psychologist. 1994;8(1):37-57. <https://doi.org/10.1123/tsp.8.1.37>

17. de Matos LF, Bertollo M, Stefanello JMF, Pires FO, da Silva CK, Nakamura FY, et al. Motivational self-talk improves time-trial swimming endurance performance in amateur triathletes. Int J Sport Exerc Psychol. 2021;19(3):446–59. <https://doi.org/10.1080/1612197X.2020.1717576>

18. Donohue B, Barnhart R, Covassin T, Carpin K, Korb E. The development and initial evaluation of two promising mental preparatory methods in a sample of female cross country runners. Journal of Sport Behavior. 2001;24(1):19-30.

19. Gray SW, Fernandez SJ. Effects of Visuo-Motor Behavior Rehearsal with videotaped modeling on basketball shooting performance. Psychology: A Journal of Human Behavior. 1989;26(4):41-7.

20. Gross M, Moore ZE, Gardner FL, Wolanin AT, Pess R, Marks DR. An empirical examination comparing the Mindfulness-Acceptance-Commitment approach and Psychological Skills Training for the mental health and sport performance of female student athletes. Int J Sport Exerc Psychol. 2018;16(4):431-51. <https://doi.org/10.1080/1612197x.2016.1250802>

21. Gröpel P. Self-focused attention and motor skill failure: The moderating role of action orientation. Sport, Exercise, and Performance Psychology. 2016;5(3):206-17. <https://doi.org/10.1037/spy0000059>

22. Guillot A, Moschberger K, Collet C. Coupling movement with imagery as a new perspective for motor imagery practice. Behavioral and Brain Functions. 2013;9, 8. <https://doi.org/10.1186/1744-9081-9-8>

23. Guillot A, Nadrowska E, Collet C. Using motor imagery to learn tactical movements in basketball. Journal of Sport Behavior. 2009;32(2):189-206.

24. Hall EG, Erffmeyer ES. The effect of Visuo-Motor Behavior Rehearsal with videotaped modeling on free throw accuracy of intercollegiate female basketball players. Journal of Sport Psychology. 1983;5(3):343-6. <https://doi.org/10.1123/jsp.5.3.343>

25. Halperin I, Chapman DW, Thompson KG, Abbiss C. False-performance feedback does not affect punching forces and pacing of elite boxers. Journal of Sport Sciences. 2019;37(1):59-66. <https://doi.org/10.1080/02640414.2018.1482526>

26. Hammoudi-Nassib S, Nassib S, Chtara M, Briki W, Chaouachi A, Tod D, et al. Effects of psyching-up on sprint performance. Journal of Strength & Conditioning Research. 2017;31(8):2066-74. <https://doi.org/10.1519/JSC.0000000000000373>

27. Holm JE, Beckwith BE, Ehde DM, Tinius TP. Cognitive-behavioral interventions for improving performance in competitive athletes: A controlled treatment outcome study. Int J Sport Psychol. 1996;27(4):463-75.

28. Horton AM, Jr., Shelton JK. The rational wrestler—A pilot study. Percept Mot Skills. 1978;46:882. <https://doi.org/10.2466/pms.1978.46.3.882>

29. Hut M, Minkler TO, Glass CR, Weppner CH, Thomas HM, Flannery CB. A randomized controlled study of mindful sport performance enhancement and psychological skills training with collegiate track and field athletes. J Appl Sport Psychol. 2021:1-23. <https://doi.org/10.1080/10413200.2021.1989521>

30. Ille A, Selin I, Do M-C, Thon B. Attentional focus effects on sprint start performance as a function of skill level. Journal of Sport Sciences. 2013;31(15):1705-12. <https://doi.org/10.1080/02640414.2013.797097>

31. Kacperski C, Hall C. Do construal levels affect athletes' imagery and performance outcomes? It depends on the task! J Appl Sport Psychol. 2017;29(2):181-98. <https://doi.org/10.1080/10413200.2016.1220992>

32. Kanthack TFD, Guillot A, Altimari LR, Nunez Nagy S, Collet C, Di Rienzo F. Selective efficacy of static and dynamic imagery in different states of physical fatigue. PLoS One. 2016;11(3):e0149654. <https://doi.org/10.1371/journal.pone.0149654>

33. Klatt S, Noël B. Regulatory focus in sport revisited: Does the exact wording of instructions really matter? Sport, Exercise, and Performance Psychology. 2020;9(4):532–42. <https://doi.org/10.1037/spy0000195>

34. Kutzner FLW, Förderer S, Plessner H. Regulatory fit improves putting in top golfers. Sport, Exercise, and Performance Psychology. 2013;2(2):130-7. <https://doi.org/10.1037/a0030733>

35. Lamirand M, Rainey D. Mental imagery, relaxation, and accuracy of basketball foul shooting. Percept Mot Skills. 1994;78:1229-30. <https://doi.org/10.2466/pms.1994.78.3c.1229>

36. Liang D, Chen S, Zhang W, Xu K, Li Y, Li D, et al. Investigation of a Progressive Relaxation Training Intervention on Precompetition Anxiety and Sports Performance Among Collegiate Student Athletes. Frontiers in Psychology. 2021;11(617541). <https://doi.org/10.3389/fpsyg.2020.617541>

37. Madden G, McGown C. The effect of hemisphericity, imagery, and relaxation on volleyball performance. Journal of Human Movement Studies. 1988;14:197-204.

38. Maynard IW, Hemmings B, Warwick-Evans L. The effects of a somatic intervention strategy on competitive state anxiety and performance in semiprofessional soccer players. The Sport Psychologist. 1995;9(1):51-64. <https://doi.org/10.1123/tsp.9.1.51>

39. Maynard IW, Smith MJ, Warwick-Evans L. The effects of a cognitive intervention strategy on competitive state anxiety and performance in semiprofessional soccer players. Journal of Sport and Exercise Psychology. 1995;17(4):428-46. <https://doi.org/10.1123/jsep.17.4.428>

40. McAleney PJ, Barabasz A, Barabasz M. Effects of flotation restricted environmental stimulation on intercollegiate tennis performance. Percept Mot Skills. 1990;71(3):1023-8. <https://doi.org/10.2466/PMS.71.7.1023-1028>

41. Milley KR, Ouellette GP. Putting Attention on the Spot in Coaching: Shifting to an External Focus of Attention With Imagery Techniques to Improve Basketball Free-Throw Shooting Performance. Frontiers in Psychology. 2021;12(645676). <https://doi.org/10.3389/fpsyg.2021.645676>

42. Minkler TO, Glass CR, Hut M. Mindfulness training for a college team: Feasibility, acceptability, and effectiveness from within an athletic department. J Appl Sport Psychol. 2021;33(6):609-26. <https://doi.org/10.1080/10413200.2020.1739169>

43. Mumford B, Hall C. The effects of internal and external imagery on performing figures in figure skating. Canadian Journal of Applied Sport Sciences. 1985;10(4):171-7.

44. Neumann DL, Hohnke E. Practice using performance goals enhances basketball free throw accuracy when tested under competition in elite players. J Hum Sport Exerc. 2018;13(2):296-304.

45. Noel RC. The effect of Visuo-Motor Behavior Rehearsal on tennis performance. Journal of Sport Psychology. 1980;2(3):221-6. <https://doi.org/10.1123/jsp.2.3.221>

46. Padgett VR, Hill AK. Maximizing athletic performance in endurance events: A comparison of cognitive strategies. Journal of Applied Social Psychology. 1989;19(4):331-40. <https://doi.org/10.1111/j.1559-1816.1989.tb00058.x>

47. Palao JM, García-de-Alcaraz A, Hernández-Hernández E, Ortega E. A case study of applying collective technical-tactical performance goals in elite men’s volleyball team. International Journal of Applied Sports Sciences. 2016;28(2):68-78.

48. Papaioannou A, Theodorakis Y, Ballon F, Auwelle YV. Combined effect of goal setting and self-talk in performance of a soccer-shooting task. Percept Mot Skills. 2004;98(1):89-99. <https://doi.org/10.2466/pms.98.1.89-99>

49. Plessner H, Unkelbach C, Memmert D, Baltes A, Kolb A. Regulatory fit as a determinant of sport performance: How to succeed in a soccer penalty-shooting. Psychol Sport Exerc. 2009;10(1):108-15. <https://doi.org/10.1016/j.psychsport.2008.02.001>

50. Reeves JL, Tenenbaum G, Lidor R. Choking in front of the goal: The effects of self‐consciousness training. Int J Sport Exerc Psychol. 2007;5(3):240-54. <https://doi.org/10.1080/1612197X.2007.9671834>

51. Savoy C, Beitel P. Mental imagery for basketball. Int J Sport Psychol. 1996;27:454-62.

52. Schorer J, Jaitner T, Wollny R, Fath F, Baker J. Influence of varying focus of attention conditions on dart throwing performance in experts and novices. Experimental Brain Research. 2012;217:287–97. <https://doi.org/10.1007/s00221-011-2992-5>

53. Schreiber EH. Using hypnosis to improve performance of college basketball players. Percept Mot Skills. 1991;72(2):536-8. <https://doi.org/10.2466/PMS.72.2.536-538>

54. Scott VB, Jr., Robare RD, Raines DB, Konwinski SJM, Chanin JA, Tolley RS. Emotive writing moderates the relationship between mood awareness and athletic performance in collegiate tennis players. North American Journal of Psychology. 2003;5(2):311-24.

55. Shahi MRS, Ezabadi RS, Abootalebi N, Moshiri P. The Effects of the Self-Talk Types and Task Complexity on the Accuracy of Forehand Topspin of Advanced Players. International Journal of Sports Science and Physical Education. 2020;5(2):16-20. <https://doi.org/10.11648/j.ijsspe.20200502.12>

56. Shoenfelt EL. Goal setting and feedback as a posttraining strategy to increase the transfer of training. Percept Mot Skills. 1996;83(1):176-8. <https://doi.org/10.2466/pms.1996.83.1.176>

57. Shoenfelt EL, Griffith AU. Evaluation of a mental skills program for serving for an intercollegiate volleyball team. Percept Mot Skills. 2008;107(1):293-306. <https://doi.org/10.2466/pms.107.1.293-306>

58. Simões P, Vasconcelos-Raposo J, Silva A, Fernandes HM. Effects of a process-oriented goal setting model on swimmer’s performance. Journal of Human Kinetics. 2012;32:65–76. <https://doi.org/10.2478/v10078-012-0024-6>

59. Smith D, Holmes P. The effect of imagery modality on golf putting performance. Journal of Sport and Exercise Psychology. 2004;26(3):385-95. <https://doi.org/10.1123/jsep.26.3.385>

60. Smith D, Wright C, Allsopp A, Westhead H. It’s all in the mind: PETTLEP-based imagery and sports performance. J Appl Sport Psychol. 2007;19(1):80-92. <https://doi.org/10.1080/10413200600944132>

61. Smith D, Wright CJ, Cantwell C. Beating the bunker: The effect of PETTLEP imagery on golf bunker shot performance. Research Quarterly for Exercise & Sport. 2008;79(3):385-91. <https://doi.org/10.1080/02701367.2008.10599502>

62. Spindler DJ, Allen MS, Vella SA, Swann C. Motivational-general arousal imagery does not improve decision-making performance in elite endurance cyclists. Cognition and Emotion. 2019;33(5). <https://doi.org/10.1080/02699931.2018.1529656>

63. Stamou E, Theodorakis Y, Kokaridas D, Perkos S, Kessanopoulou M. The effect of self-talk on the penalty execution in goalball. British Journal of Visual Impairment. 2007;25(3):233–47. <https://doi.org/10.1177/0264619607079800>

64. Takeuchi T, Ikudome S, Unenaka S, Ishii Y, Mori S, Mann DL, et al. The inhibition of motor contagion induced by action observation. PLoS One. 2018;13(10):e0205725. <https://doi.org/10.1371/journal.pone.0205725>

65. Vast R, Young R, Thomas PR. Emotion and automaticity: Impact of positive and negative emotions on novice and experienced performance of a sensorimotor skill. Int J Sport Exerc Psychol. 2011;9(3):227-37. <https://doi.org/10.1080/1612197X.2011.614848>

66. Velentzas K, Heinen T, Schack T. Routine integration strategies and their effects on volleyball serve performance and players’ movement mental representation. J Appl Sport Psychol. 2011;23(2):209-22. <https://doi.org/10.1080/10413200.2010.546826>

67. Wagaman JD, Barabasz AF, Barabasz M. Flotation REST and imagery in the improvement of collegiate basketball performance. Percept Mot Skills. 1991;72(1):119-22. <https://doi.org/10.2466/pms.1991.72.1.119>

68. Weinberg R, Miller A, Horn T. The influence of a self-talk intervention on collegiate cross-country runners. Int J Sport Exerc Psychol. 2012;10(2):123-34. <https://doi.org/10.1080/1612197X.2012.645135>

69. Weinberg R, Stitcher T, Richardson P. Effects of a seasonal goal-setting program on lacrosse performance. The Sport Psychologist. 1994;8(2):166-75. <https://doi.org/10.1123/tsp.8.2.166>

70. Wergin VV, Beckmann J, Gröpel P, Mesagno C. Investigating cumulative effects of pre-performance routine interventions in beach volleyball serving. PLoS One. 2020;15(1):e0228012. <https://doi.org/10.1371/journal.pone.0228012>

71. Westlund Stewart N, Hall C. The effects of cognitive general imagery use on decision accuracy and speed in curling. The Sport Psychologist. 2016;30(4):305-13. <https://doi.org/10.1123/tsp.2016-0001>

72. Whdan N. Effects of relaxation training on muscle tension and the performance level of 50m front crawl swimming. Science, Movement and Health. 2014;14(1):143-8.

73. Winter S, Collins D. Does priming really put the gloss on performance? Journal of Sport and Exercise Psychology. 2013;35(3):299-307. <https://doi.org/10.1123/jsep.35.3.299>

74. Wojcikiewicz A, Orlick T. The effects of post-hypnotic suggestion and relaxation with suggestion on competitive fencing anxiety and performance. Int J Sport Psychol. 1987;18(4):303-13.

75. Wolanin AT, Schwanhausser LA. Psychological functioning as a moderator of the MAC approach to performance enhancement. Journal of Clinical Sport Psychology. 2010;4(4):312-22. <https://doi.org/10.1123/jcsp.4.4.312>

76. Wollman N, Hill J, Lipsitz T. Effects of imagery on track and bowling performance in naturalistic settings. Percept Mot Skills. 1985;60(3):986. <https://doi.org/10.2466/pms.1985.60.3.986>

77. Woolsey TD, Woolsey CL, Strohmeyer S, Walker S, Otto W, Cheshier BC, et al. The Effect of Advanced Imagery Training on Shot Putter Performance. Journal of Contemporary Athletics. 2020;14(4):271-9.

78. Wu T-Y, Nien J-T, Kuan G, Wu C-H, Chang Y-C, Chen H-C, et al. The Effects of Mindfulness-Based Intervention on Shooting Performance and Cognitive Functions in Archers. Frontiers in Psychology. 2021;12(661961). <https://doi.org/10.3389/fpsyg.2021.661961>

79. Zervas Y, Kakkos V. Visuomotor behavior rehearsal in archery shooting performance. Percept Mot Skills. 1991;73(3, Pt 2):1183-90. <https://doi.org/10.2466/PMS.73.8.1183-1190>
